# New Anti-angiogenic Leading Structure Discovered in the Fruit of *Cimicifuga yunnanensis*

**DOI:** 10.1038/srep09026

**Published:** 2015-03-12

**Authors:** Yin Nian, Jing Yang, Tong-Yang Liu, Ying Luo, Ji-Hong Zhang, Ming-Hua Qiu

**Affiliations:** 1 State Key Laboratory of Phytochemistry and Plant Resources in West China, Kunming Institute of Botany, Chinese Academy of Sciences, Kunming 650201, P. R. China; 2 Laboratory of Molecular Genetics of Aging & Tumor, Faculty of Medicine, Kunming University of Science & Technology, Kunming. 650500, P. R. China

## Abstract

Cimyunnins A–C (1–3), characterized with an unusual fused cyclopentenone ring G, together with cimyunnin D (4), possessing a highly rearranged *γ*-lactone ring F, were characterized from the fruit of *Cimicifuga yunnanensis*. Their structures were elucidated by spectroscopic analysis, X-ray diffraction, and density functional theory calculations. In addition, cimyunnin A exhibited comparable anti-angiogenic activities to those of sunitinib, a clinically-used first-line angiogenesis inhibitor, in the *in vitro* and *ex vivo* studies.

Pathological angiogenesis is a pivotal process for a broad range of diseases, including cancer, rheumatoid arthritis, age-related macular degeneration, diabetic retinopathy, psoriasis and atherosclerosis^1^,^2^. Thus, anti-angiogenesis has been identified as an attractive target in modern drug discovery. Notably, a number of anti-angiogenic agents, such as sunitinib and pegaptanib, have been approved by FDA for the treatment of cancer and age-related macular degeneration, respectively^1^.

Plants of *Cimicifuga* genus are famous herb medicines worldwide^3^. Up until now, more than 300 cycloartane triterpenoids (CTs) have been isolated (among them, more than 100 compounds were discovered by our research group^3^,^4^,^5^,^6^,^7^,^8^,^9^,^10^,^11^,^12^,^13^,^14^,^15^,^16^,^17^) from the genus^3^,^4^,^5^,^6^,^7^,^8^,^9^,^10^,^11^,^12^,^13^,^14^,^15^,^16^,^17^,^18^,^19^,^20^,^21^. These secondary metabolites showed divers bioactivities, such as cytotoxicity^3^,^4^,^5^,^6^,^7^,^8^,^9^,^10^,^11^,^12^,^13^,^14^,^15^,^16^, antiosteoporotic^22^, anti-AIDS^23^, anti-Alzheimer^24^, and immunosuppression^25^. To date, however, anti-angiogenic knowledge of CTs from *Cimicifuga* spp is mainly not yet involved.

*C. yunnanensis* is a rare species distributed in the southwest region of China^8^. Our previous studies on the roots of *C. yunnanensis* led to the discovery of three cytotoxic CTs, which showed as potent activities as taxol against drug resistant breast cancer cell line R-MCF-7. Furthermore, it was found that the p53-dependent mitochondrial signaling pathway contributed to the apoptosis activities induced by these compounds^26^. More recently, a series of active CTs against *p53^N236S^* mouse embryonic fibroblasts were obtained from the overground parts of this plant^3^. However, there is no current literature reported the chemical components and their bioactivities of the fruit of *C. yunnanensis*. This study aimed to fill this gap in the knowledge and has subsequently isolated and identified, two unprecedented types of CTs, cimyunnins A–C (**1–3**) and cimyunnin D (**4**). Compounds **1–3** contains a fused cyclopentenone ring G, which formed by direct carbon-carbon linkage between C-22 and C-26. While, **4** features a highly rearranged *γ*-lactone ring F among C-23 to C-27 (Fig. 1). Biological assay revealed that compound **1** showed significant anti-angiogenic properties both *in vitro* and *ex vivo*. Moreover, a mechanism study indicated that **1** exerted its anti-angiogenic activities by directly targeting VEGFR2-signaling pathways.

## Results and Discussion

### Structural Elucidation of Compounds 1–4

Cimyunnin A (**1**), a white powder, with a molecular formula of C_30_H_44_O_4_, as established by HREIMS (*m*/*z* 486.3235, calcd for [M]^+^ 486.3240), requires nine sites of unsaturation. The ^1^H-NMR spectrum (Table S1) displayed the presence of characteristic cyclopropane methylene signals at *δ*_H_ 0.26 (d, *J* = 3.8 Hz) and 0.67 (d, *J* = 3.4 Hz). The ^13^C NMR and DEPT (Table S1) spectra showed the existence of one ketone (*δ*_C_ 203.5), and one tetrasubstituted double bond (*δ*_C_ 148.9 and 149.3). Aforementioned data suggested that **1** was a CTs with a seven-ring skeleton^3^.

**Figure 2:**
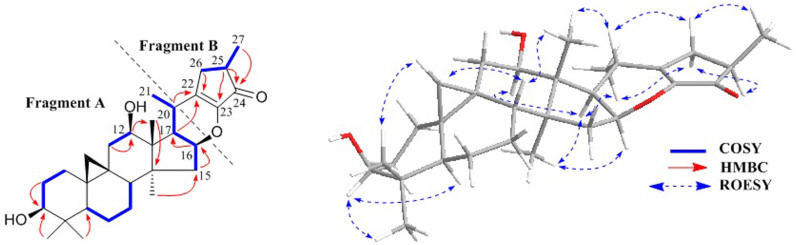
Fragments, key COSY and HMBC correlations of 1.

The NMR data for rings A, B, C, D, and E of **1** were similar to those of asiaticoside A (**5**)^18^, except that the sugar unit at C-3 and the acetoxy group at C-12 were replaced by two hydroxy groups, respectively. This deduction was confirmed by the ^1^H-^1^H COSY correlations (Figure 2) of hydroxymethine protons at *δ*_H_ 3.50 (H-3), and *δ*_H_ 4.23 (H-12) with H-2 (*δ*_H_ 1.81 and 1.93) and H-11 (*δ*_H_ 1.42 and 2.62), respectively. Further study of the ^1^H-^1^H COSY spectrum starting from the H-16 at *δ*_H_ 4.52 revealed the presence of a spin system - CHCHCH-(CH_3_)- (for C-16, C-17, C-20, and CH_3_-21) in **1**. Thus, the fragment A of **1** was constructed as shown (Figure 2).

The spin system -CH_2_CHCH_3_- due to C-26, C-25, and C-27 in fragment B was also deduced from ^1^H-^1^H COSY correlations (Fig. 2). Besides, HMBC correlations (Fig. 2) of H-20 (*δ*_H_ 3.30) and H-26 (*δ*_H_ 2.67, 1.73) with the quaternary olefinic carbon at *δ*_C_ 148.9 (C-22), required the connections of C-20 and C-26 to C-22. Further analyses of the HMBC spectrum showed the correlations from H-25 (*δ*_H_ 2.32) to the carbonyl group at *δ*_C_ 203.5 (C-24) and the olefinic carbon at *δ*_C_ 149.3 (C-23). This information coupled with the UV and IR absorptions of *λ*_max_ 266 nm, and *ν*_max_ 1702 and 1655 cm^−1^, indicated the existence of a cyclopentenone ring G (C-22 to C-26). Therefore, to fulfill the molecular formula and unsaturation requirement, C-16 and C-23 should be connected by an oxygen atom, which also supported by similar chemical shifts of C-22 (*δ*_C_ 148.9) and C-23 (*δ*_C_ 149.3) due to the electronic effect and conjugative effect from the oxygen atom at C-16 and carbonyl group at C-24, repectively. Finally, fragment B and planar structure of **1** were established.

**Figure 3:**
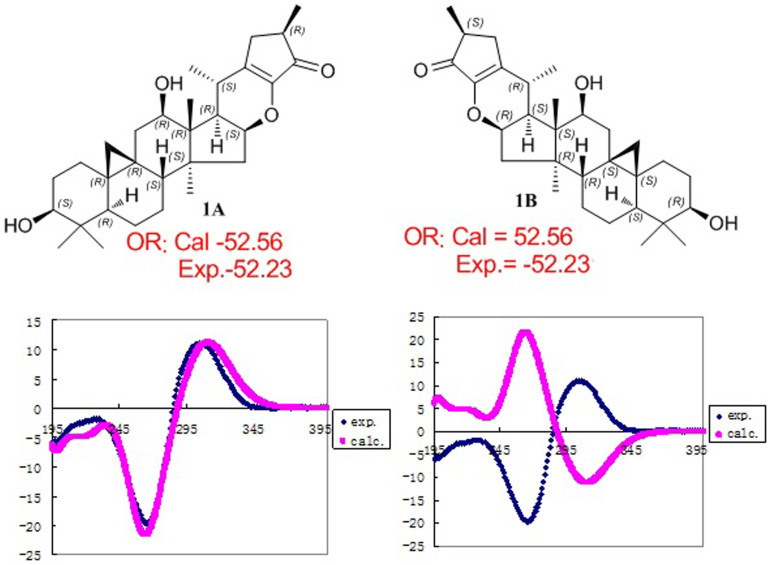
Assignment of the absolute configuration of 1 by comparison of the experimental CD spectra with the spectra calculated for 1A and 1B using TDDFT methods.

In the ROESY spectrum (Figure 2), correlations of H-5 (biogenetically *α*-oriented)/H-3, Me-28 (biogenetically *α*-oriented)/H-17, Me-28/H-16, H-17/H-12, H-17/Me-21, H-8/Me-18 (biogenetically *β*-oriented), Me-18/H-20, H-20/H-26*β*, H-26*β*/Me-27, and H-26*α*/Me-21 were observed, which helped to establish the relative configuration of **1**. Our efforts to make fine crystals from **1** failed which precluded the possibility to determine the absolute configuration directly by X-ray crystallography. Therefore, quantum chemical CD calculations were applied^27^. The results showed that spectrum calculated for **1A** was nearly identical with the experimental data of **1** (Fig. 3, left) over the whole range of wavelengths under investigation, whereas the spectrum simulated for **1B** exhibited very different CD behavior compared with the experimental CD curve of **1** (Fig. 3, right). Therefore, the absolute configuration of **1** was determined as shown.

Cimyunnins B (**2**) and C (**3**), obtained as an inseparable mixture. Side-by-side comparison of their NMR data indicated that **2** and **3** were a pair of C-25 epimers and most signals were exchangeable between the two compounds (Table S2). Whereas, the signals of Me-27 (*δ*_H_ 1.03 in compound **2** and *δ*_H_ 1.00 in compound **3**) and H-24a (*δ*_H_ 2.68 in compound **2** and *δ*_H_ 2.65 in compound **3**) showed clear differences between compounds **2** and **3**. In the ROESY spectrum, the correlations of H-20 (*δ*_H_ 2.83) with Me-18 (*δ*_H_ 0.76, biogenetically *β*-oriented) and Me-27 (*δ*_H_ 1.03) were observed, indicating the *β*-orientation of Me-27 in compound **2**. Therefore, the configuration of Me-27 in compound **3** was *α*-orientation. ^13^C NMR signals for C-24 (*δ*_C_ 34.52), C-25 (*δ*_C_ 38.04) and C-26 (*δ*_C_ 205.13) were able to ascribed to compound **2** on the basis of their HMBC correlations with Me-27 at *δ*_H_ 1.03. Similarly, the ^13^C NMR signals for C-22 and C-23 were successfully assigned based on HMBC correlations between H-24a of compounds **2** and **3** and corresponding carbons.

The ^1^H and ^13^C NMR spectra (Table S2) of **2** and **3** were very similar to those of **1** with the major differences for signals of ring G. A spin system -CH_2_CHCH_3_- in the ring G was established by the ^1^H-^1^H COSY spectrum, which coupled with extreme downfield double bond (C-23) at *δ*_C_ 180.15 (180.20) positioned the cabonyl carbon at C-26. Fortunately, crystals of the epimers (1:1 mixsture) were obtained and X-ray diffraction analysis (Figure S1) allowed to establish the structures of **2** and **3** and further confirm the new skeleton of this type of triterpenoid (**1–3**).

**Figure 4:**
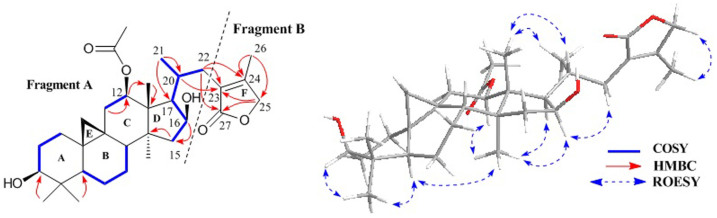
Fragments, key COSY and HMBC correlations of 4.

The molecular composition of cimyunnin D (**4**), C_32_H_48_O_6_, was deduced from HR-EIMS ([M]^+^, *m/z* 528.3449), indicating 9 degrees of unsaturation. In the ^1^H NMR spectrum (Table S3) of **4**, signals due to a typical cyclopropane methylene at *δ*_H_ 0.27 (d, *J* = 4.1 Hz) and 0.46 (d, *J* = 3.7 Hz), an acetoxy methyl group at *δ*_H_ 2.03, a secondary methyl resonance at *δ*_H_ 0.86 (d, *J* = 6.8 Hz), and five tertiary methyl groups at *δ*_H_ 0.85–1.84 (each 3H, s) were observed, in agreement with a structure of a CTs with an acetoxy group^3^. The ^1^H-^1^H COSY correlations (Fig. 4) of H-2 (*δ*_H_ 1.19 and 1.80) with the proton (*δ*_H_ 3.39) of the hydroxymethine at *δ*_C_ 78.3 and H-15 (*δ*_H_ 1.77 and 2.06) with the proton (*δ*_H_ 4.67) of the hydroxymethine at *δ*_C_ 72.1, located the hydroxy groups at C-3 and C-16, respectively. In the HMBC spectrum, correlation of H-12 at *δ*_H_ 5.09 and the carbonyl carbon at *δ*_C_ 171.2 was observed, indicating an acetoxy group were connected to C-12. Further analyses of ^1^H-^1^H COSY associations disclosed that **4** had a partial structure of -CH_2_CHCHCH-(CH_3_)CH_2_- (for C-15 to C-17, C-20 to C-22). Thus, fragment A was constructed as shown (Fig. 4).

**Figure 5:**
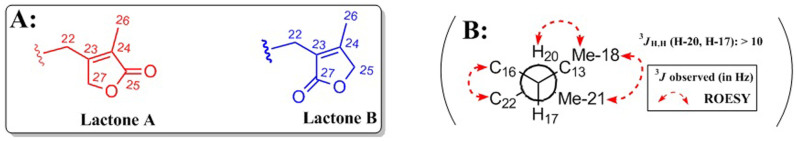
Structure elucidation of 4. (A) The possible structures of ring F for **4**; (B) Determination of relative stereochemistry of coupling systems of C-17/C-20 in **4**.

Apart from fragment A, an isolated methyl (Me-26, *δ*_H_ 1.84, s), an oxygenated methylene (C-25, *δ*_H_ 4.55 and 4.59, each 1H, d, *J* = 17.0 Hz), an ester carbonyl carbon (C-27, *δ*_C_ 177.3), and two quaternary olefinic carbons (C-23, *δ*_C_ 127.1; and C-24 *δ*_C_ 159.7) were observed in **4**. Thus, there was still one degree of unsaturation unaccounted for, requiring another ring in the final structure. The existence of an oxygenated methylene indicating the esterification of C-25 by C-27, which allowed to construct two possible five-membered ring F of **4**, lactone A and lactone B (Fig. 5A). In addition, significant HMBC correlations from H_2_-22 (*δ*_H_ 1.93 and 2.71) to the carbonyl carbon at C-27 (*δ*_C_ 177.3) and ROESY associations of H-25 (*δ*_H_ 4.55 and 4.59) with CH_3_-26 (*δ*_H_1.84) indicated that the strucutre of lactone B is reasonable.

On the basis of similar ROESY correlations (Fig. 4), same relative stereochemistries of C-3, C-5, C-8, C-9, C-10, C-12, C-13, C-14, C-16, and C-17 in **4** as in **1** were elucidated. Figure 5B depicted the relative stereochemistry of the C-17/C-20 coupling system by Newman projection. The anti-orientation of H-17/H-20 was suggested by a large ^3^*J*_H,H_ (>10.0 Hz), while the gauche orientations of H-17/CH_3_-21 and H-20/C-16 were supported by the ROESY correlations of H-20/CH_3_-18, CH_3_-21/CH_3_-18, and H-16/H-22b. Finally, the ^13^C NMR and OR data of **4** were compared to the calculated results of lactone **A** (**4A**) and lactone **B** (**4B**) (Table S9, S10, and S11), which further confirmed the absolute configuration of **4**.

To the best of our knowledge, compounds **1–4** stand for two unprecedented classes of CTs. Biosynthetically, **1–3** probably originate from the cycloartane precursors asiaticoside A (**5**)^18^. The key steps are the formation of intermediates **5A**, and **5B** through nazarov reaction^28^, and [2 + 2] cycloaddition^29^, respectively. While, compound **4** may derive from **1** through a series of oxidation rearrangements (see Scheme 1 in the Supporting Information).

### *In vitro* and *Ex vivo* Anti-angiogenic Activities of Compound 1

**Figure 6:**
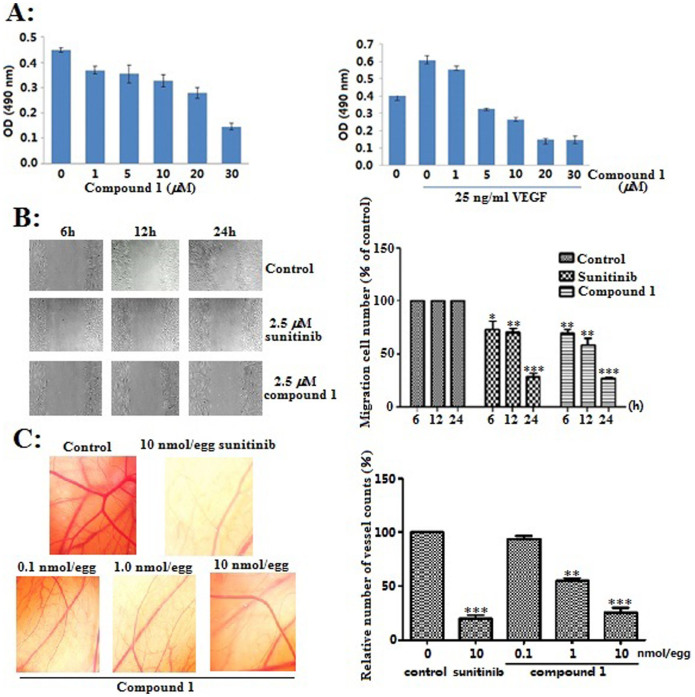
*In vitro* and *ex vivo* anti-angiogenic activities of compound 1. (A) The proliferation inhibitory effect of **1** against normal and VEGF-induced HUVECs; (B) Compound **1** inhibited the VEGF-induced migration of HUVECs. Cells were wounded with a pipette then treated with vehicle or 2.5 *μ*M sunitinib and compound **1**. After 6, 12 and 24 h, the migrated cells were quantified by the Image Pro Plus software; (C) Compound **1** inhibited *ex vivo* angiogenesis in CAM assay, quantification of the number of new vascular growth counts was performed with Image Pro Plus software. *p < 0.05, **p < 0.01, ***p < 0.001 *vs* vehicle control, data were analyzed by using Graphpad student t test (n = 3).

Although CTs from *Cimicifuga* spp showed various bioactivities, anti-angiogenic activities were still unknown. In the present study, compound **1** notably inhibited VEGF-induced proliferation of HUVECs at 5.0 *μ*M (due to sample quantity limitation, the activities of compounds **2–4** were not studied), without obvious cytotoxicity against human umbilical vein endothelial cells (HUVECs) (Fig. 6A). Then, the effect of **1** on the motility of HUVECs was studied. As shown in Figure 6B, HUVECs migrated into the clear area when stimulated with 25 ng/ml VEGF. Conversely, compound **1** significantly inhibited the VEGF-induced migration of HUVECs in a time dependent manner at concentration of 2.5 *μ*M. This effect of **1** as strong as that of sunitinib, a clinically-used first-line angiogenesis inhibitor. Subsequently, **1** drastically reduced the new vascular growth density at a dose-dependent manner in the chick chorioallantoic membrane (CAM) assay. The maximum reduction of new vascular density was observed at concentration of 10.0 nmol/egg (Figure 6C), which was comparable to that of sunitinib at the same concentration. The aforementioned results indicated the anti-angiogenic potential of **1** both *in vitro* and *ex vivo*.

**Figure 7:**
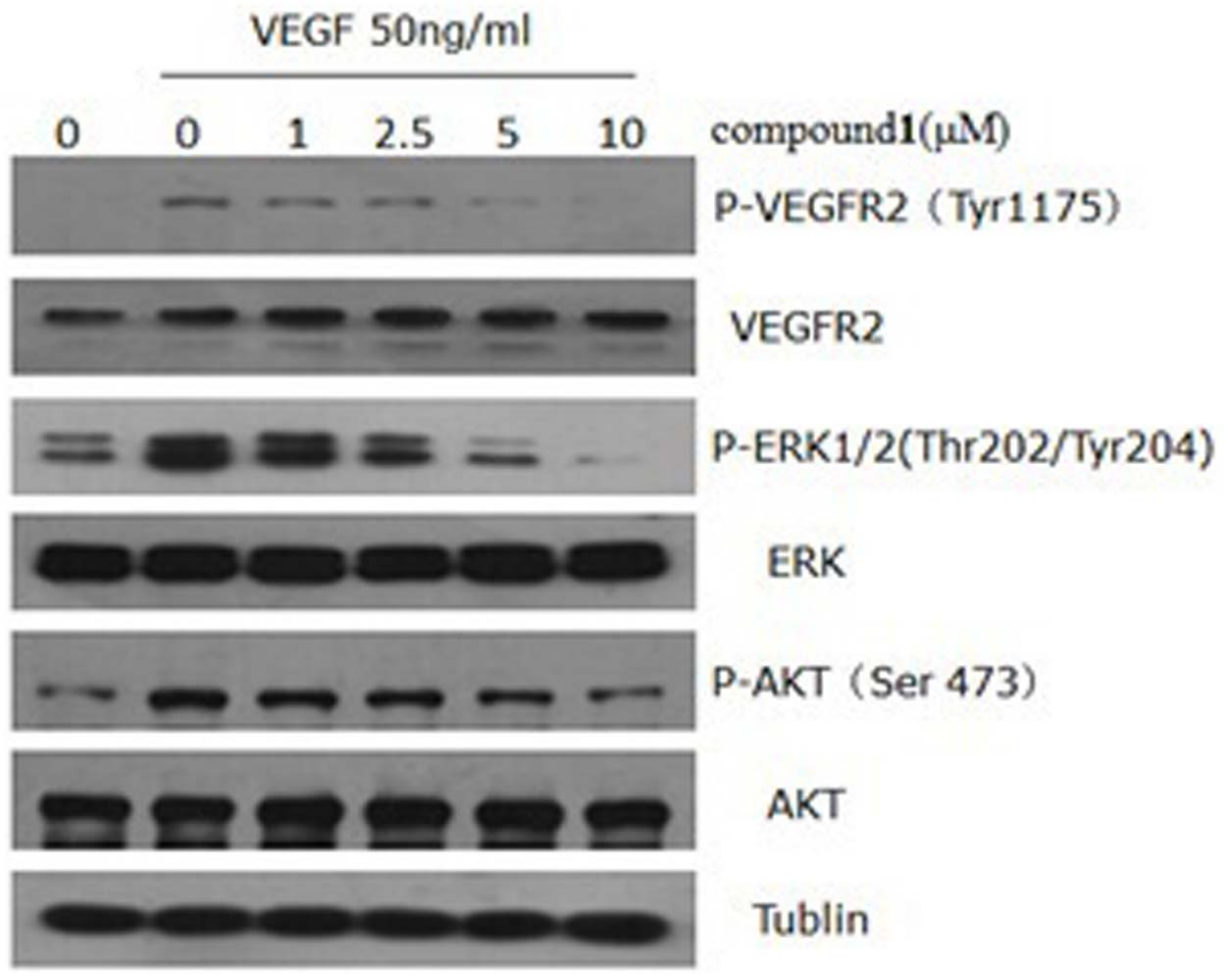
Compound 1 inhibited the activation of VEGFR2-mediated signaling pathways in HUVECs. Proteins (12 *μ*g) from the whole cell lysates were separated by 12% SDS-polyacrylamide gel electrophoresis. The full-length gel and blots were in supplementary materials.

VEGFR2-signaling pathways is essential for the function of vascular endothelial cells^30^. Finally, to understand the molecular mechanism of **1**, we examined the pathways and signaling molecules using western blot. As shown in Figure 7, phosphorylation of VEGFR2 was suppressed by **1** in a dose-dependent manner. Dramatic downregulations of phosph-AKT (Ser473) and phospho-ERK (Thr202/Tyr204), well-known downstream targets of VEGFR2, were observed at 5.0 and 10.0 *μ*M of **1**. However, total VEGFR2, ERK, and AKT remain unchanged. Therefore, compound **1** exerted its anti-angiogenic effect through directly targeting VEGFR2 on the surface of endothelial cells and further antagonizing VEGFR2-mediated downstream signaling cascade.

Natural products, due to unrivaled chemical diversity and structural plasticity, are rich sources of novel leading structures for drug discovery^31^. So far, diverse natural compounds, such as anthocyanins, genistein, resveratrol, curcumin, taxol, betulinic acid, and squalamine, showed anti-angiogenic activities both *in vitro* and *in vivo*^32^. However, few studies about the anti-angiogenic CTs were reported^33^. Herein, compound **1**, with an unprecedented skeleton, showed same level of anti-angiogenic activities as sunitinib both *in vitro* and *ex vivo*. Taken together, compound **1** stands for a new type of leading structure of anti-angiogenesis.

## Methods

### General Experimental Procedures

Optical rotations were obtained with a JASCO P-1020 digital polarimeter, using MeOH as solvent. UV spectra were taken on Shimadzu 2401PC spectrophotometer. CD spectra were obtained by a Chirascan instrument. ^1^H, ^13^C and 2D NMR experiments were measured on Bruker DRX-500 and Avance III-600 MHz spectrometers (Bruker, Zürich, Switzerland) with the solvent signal as internal reference. Mass spectra were collected from a VG Autospec-3000 spectrometer. BRUKER Tensor-27 instrument were used to record infrared spectra (with KBr pellets). X-ray diffraction was realized on a Bruker SMART APEX CCD crystallography system. Precoated TLC plates (200–250 *μ*m thickness, silica gel 60 F_254_, Qingdao Marine Chemical, Inc.) were used for the thin-layer chromatography. Semipreparative HPLC was taken on an Agilent 1100 liquid chromatography, the column used was an YMC-Pack 10 mm × 250 mm column (Pro C18 RS). Column chromatography (cc) was performed on silica gel (200–300 mesh; Qingdao Marine Chemical Inc., P. R. China), on C-18 silica gel (40–60 *μ*m; Merck), and on Sephadex LH-20 (Amersham Pharmacia, Sweden).

### Plant Material

The fruit of *Cimicifuga yunnanensis* (234 g) were collected during September 2012 from Daocheng County, Sichuan Province, China. The herbarium specimen was authenticated by Prof. Shengji Pei, Kunming Institute of Botany, CAS. A voucher specimen (KUN No. 201209002) was deposited at the State Key Laboratory of Phytochemistry and Plant Resources in West China, Kunming Institute of Botany, CAS, P. R. China.

### Extraction and Isolation

The powdered and air-dried fruit of *C. yunnanensis* (234 g) were soaked in methanol at room temperature (1.5 L × 24 h × 3). The combined extracts were concentrated under reduced pressure to afford a dark brown residue (24.2 g). The extract was fractionated by cc (silica gel 600 g, CHCl_3_/MeOH step gradients: 100:0, 50:1, 20:1, 10:1, 5:1, and 0:100) to afford fractions A-U. Fraction G (0.9 g) was divided into ten sub-fractions (Fractions G.1–G.10) by RP-18 cc (200 g, MeOH/H_2_O gradient from 50:50 to 100:0). Fraction G.7 (25 mg) was purified by semipreparative HPLC (eluted with CH_3_CN/H_2_O, gradient from 60:40 to 85:15) to yield compounds **1** (2.4 mg), and **4** (1.2 mg). Compounds **2** and **3** (1.3 mg) were isolated from fraction G.6 (40 mg) by semipreparative HPLC (eluted with CH_3_CN/H_2_O, gradient from 60:40 to 85:15).

### Cimyunnins B and C Single Crystal Cultivation

The clear solution of the pair of epimers (Cimyunnins B and C, 1.2 mg) in MeOH (5 mL) was added several drops of water, and then was kept at ambient temperature for slow evaporation to cultivate a single crystal suitable for X-ray crystallographic measurement.

### X-ray Crystallographic Analysis of the pair of epimers (Cimyunnins B and C)

The crystal data: C_30_H_44_O_4_, *M* = 468.65, orthorhombic, *a* = 11.6984(4) Å, *b* = 14.5084(5) Å, *c* = 15.4962(6) Å, *α* = 90.00°, *β* = 90.00°, *γ* = 90.00°, *V* = 2630.09(16) Å^3^, *T* = 100(2) K, space group *P*212121, *Z* = 4, *μ*(CuKα) = 0.599 mm^−1^, d = 1.184 mg/m^3^, crystal dimensions 0.36 × 0.36 × 0.10 mm, were collected from a Bruker APEX DUO diffractometer with a graphite monochromator (Φ/ω scans, 2θmax = 70.01°), Cu Kα radiation. The total number of independent reflections measured was 29685, of which 4478 were observed (|F|^2^ ≥ 2σ|F|^2^). The final *R_1_* = 0.0610 (*I* > 2*σ*(*I*)), and *wR*(*F*^2^) = 0.1612 (*I* > 2*σ*(*I*)). The final *R_1_* = 0.0616 (all data), and *wR*(*F*^2^) = 0.1619 (all data). The goodness of fit *F*^2^ = 1.047. Flack parameter = 0.6(3). The crystal structure of **1** was solved by direct method SHELXS-97 and the full-matrix least-squares deposited in the Cambridge Crystallographic Data Centre (deposition number: 937160).

### ECD Calculation

The theoretical calculations of compound **1** were performed using Gaussian 09^34^. Conformational analysis was initially carried out using Maestro7.5 conformational searching, together with the OPLS_2005 molecular mechanics methods. The optimized conformation geometries and thermodynamic parameters of the predominant conformations were provided (see computational data for **1** in Supplementary Information). The OPLS_2005 conformers were optimized at B3LYP/6-31G(d,p) level. The theoretical calculation of ECD was performed using time dependent Density Functional Theory (TDDFT) at B3LYP/6-31G(d,p) level in methanol with PCM model. The ECD spectra of compound **1** were obtained by weighing the Boltzmann distribution rate of each geometric conformation^35^.

The ECD spectra were simulated by overlapping Gaussian functions for each transition according to:
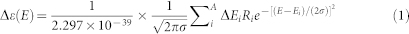
The *σ* represented the width of the band at 1/*e* height, and Δ*E_i_* and *R_i_* were the excitation energies and rotational strengths for transition *i*, respectively. *σ* = 0.30 eV and *R*_vel_ had been used in this work.

### NMR calculation

For the calculations of ^13^C NMR chemical shifts, B3LYP/6-31G(d,p) method was used to optimize the selected conformations. For all optimized structures, vibrational spectra were calculated to ensure that no imaginary frequencies for energy minimum were obtained. NMR calculations were performed at the levels of B3LYP/6-31G(d,p) with the gauge-independent atomic orbital (GIAO) method^36^,^37^,^38^. The solvent effect was considered by using pyridine in the calculations to resemble the experimental condition. The polarized continuum model (PCM) of Tomasi et al. was used^39^,^40^,^41^. The calculated ^13^C NMR chemical shifts were analyzed by subtracting the isotopic shifts for TMS calculated with the same methods^36^,^37^,^38^. Different conformers for Lactone **A** and Lactone **B** were considered. The ^13^C NMR chemical shifts in each compound were considered as the average values of the same atoms in the different conformers (see computational data for **4** in Supplementary Information). The average values were obtained by the Boltzmann distributions, using the relative Gibbs free energies as weighting factors^35^. The differences Δ*δ* were determined by subtracting the experimental chemical shifts *δ*exptl from the calculated chemical shifts*δ*scal.calc. All calculations were performed using Gaussian 09^34^.

### OR calculation

For optical rotation, compounds **1A**, lactone **A** (**4A**) and Lactone **B** (**4B**) were obtained in the gas phase at the B3LYP/6-31G(d,p) level of theory. The optical rotation values were calculated using B3LYP/6-31G(d,p) theory. The solvation effect was considered using methanol in the calculations to resemble the experimental conditions. The polarized continuum model (PCM) of Tomasi et al. was used^39^,^40^,^41^. The OR spectra of compounds **1A**, lactone **A** and Lactone **B** were obtained by weighing the Boltzmann distribution rate of each geometric conformation^35^. All calculations were performed using Gaussian 09^34^.

### Cell Growth Inhibition Assay

Growth inhibition of human cancer cells by compound **1** was assessed by the MTT assay, along with DMSO as a control. HUVECs were grown in DMEM media with 10% FBS. HUVECs were treated with compound 1 at various concentrations. After a 72 h incubation, MTT [3-(4,5-dimethylthiazol-2-yl)-2,5-diphenyltetrazolium bromide] was added to the wells (50 *μ*L; 0.4 mg/ml) and incubated another 4 h. Medium were aspirated and DMSO (150 *μ*L) was added to each well. Absorbance was measured at 490 nm using 2030 Multi-label Reader (Perkin-Elmer Victor X5, US). Compound concentrations causing 50% growth inhibition (IC_50_) were calculated.

### Cell Proliferaion Assay

HUVECs were seeded in 96-well plates and incubated for 24 h, cells were then starved in M200 medium containing 2% FBS for another 16 h. After starvation, cells were pretreated for 30 min with indicated concentration of compound **1** (1, 10, 20, 30 *μ*mol/L), followed by the stimulation with VEGF (25 ng/mL) for another 24 h. Cell viability was then determined by MTT assay.

### Wound-healing Migration Assay

HUVECs were seeded and grown into full confluence in 6 well plates. Cells were starved with 2% FBS M200 media for 12 h to inactivate cell proliferation and then wounded by pipette tips. Fresh M200 medium with 25 ng/mL VEGF containing vehicle or 2.5 *μ*mol/L sunitinib and compound **1** was added to the scratched monolayers. Images were taken after 0, 6, 12, 24 hours using an inverted microscope (magnification, 10×; Nickon). Sunitinib used as a positive control.

### CAM Assay in Fertilized Chicken Eggs

The effect of compound **1** on *ex vivo* angiogenesis was determined by CAM assay. Briefly, fertile leghorn chicken eggs (Poultry Breeding farm, Kunming) were incubated in incubator at 37.8°C with 40% humidity. A small opening was made at the top of live eggs on day 7 under aseptic conditions. Indicated concentrations of compound **1** and sunitinib was mixed with DMSO and tipped on the filter paper, then gently placed on the CAM. The eggs were incubated for 48 h, then fixed with methanol and photographed.

### Western Blot Assay

To determine the effects of compound **1** on VEGFR-2 dependent signaling pathway, HUVECs were serum-starved overnight, then pretreated with or without compound **1** (1, 2.5, 5, 10 *μ*M) for 2 h, followed by the stimulation with 50 ng/mL VEGF_165_ for 15 min. cells were lysed with buffer containing 20 mmol/L Tris, 2.5 mmol/L EDTA, 1% Triton X-100, 1% deoxycholate, 0.1% SDS, 40 mmol/L NaF, 10 mmol/L Na4P2O7, proteinase inhibitor cocktail and 1 mmol/L phenylmethylsulfonyl fluoride. Protein concentrations were determined by Bradford assay and equalized before loading. About 20 *μ*g cellular proteins were separated using gradient SDS-PAGE gels and probed with specific antibodies (Cell Signaling Technology) including phospho-VEGFR2(p-VEGFR2; Tyr1175), VEGFR2, phospho-ERK1/2 (p-ERK1/2; Thr202/Tyr204), ERK, phospho-AKT (p-AKT; Ser473), AKT and actin. Blots were developed by incubating with horseradish peroxidase-conjugated antibodies (GE health care UK) and visualized with enhanced chemiluminescene reagent (Thermo).

Cimyunnin A (**1**): white powder; [α]_D_^25^ = −52.23 (*c* 0.11, MeOH); UV (MeOH) *λ*_max_ (log ε): 202 (0.32), 266 (0.51). IR (KBr): *ν_max_* 3442, 2959, 2868, 1702, 1650, 1456, 1374, 1138, 1024, 986 cm^−1^; ^1^H (C_5_D_5_N, 600 MHz) and ^13^C NMR (C_5_D_5_N, 150 MHz) spectra see Table S1; positive ESIMS: *m/z* 491 [M + Na]^+^; HR-EIMS: *m/z* 486.3235 (calc. for C_30_H_46_O_5_, 486.3240).

Cimyunnins B and C (**2** and **3**): colorless crystals; [α]_D_^25^ = −105.99 (*c* 0.15, MeOH); UV (MeOH) *λ*_max_ (log ε): 200 (0.28), 252 (0.79); IR (KBr): *ν_max_* 3440, 2957, 2867, 1691, 1627, 1415, 1383, 1130, 1024, 990 cm^−1^; ^1^H (C_2_D_6_OS, 500 MHz) and ^13^C NMR (C_2_D_6_OS, 150 MHz) spectrum see Table S2; positive ESIMS: *m/z* 491 [M + Na]^+^; HREIMS: *m/z* 468.3249 (calc. for C_30_H_44_O_4_, 468.3240).

Cimyunnin D (**4**): white powder; [α]_D_^25^ = −51.22 (*c* 0.11, MeOH); UV (MeOH) *λ*_max_ (log ε): 215 (0.53). IR (KBr): *ν_max_* 3442, 2932, 2869, 1734, 1631, 1452, 1384, 1248, 1098, 1028, 986 cm^−1^; ^1^H (C_5_D_5_N, 600 MHz) and ^13^C NMR (C_5_D_5_N, 150 MHz) spectrum see Table S3; positive ESIMS: *m/z* 551 [M + Na]^+^; HR-EIMS: *m/z* 528.3449 (calc. for C_30_H_46_O_5_, 528.3451).

## Author Contributions

Q.M.H., Z.J.H. and N.Y. designed the phytochemical and biological experiments. Y.J. designed and conducted the density functional theory calculations. N.Y. and Z.J.H. analyzed data. N.Y., L.Y. and Z.J.H. wrote the paper. N.Y. and L.T.Y. conducted the phytochemical and biological experiments, repectively. N.Y. and Y.J. were designated as co-first authors.

## Supplementary Material

Supplementary Information Supplementary Information for New Anti-angiogenic Leading Structure Discovered in the Fruit of Cimicifuga yunnanensis

## Figures and Tables

**Figure 1:**
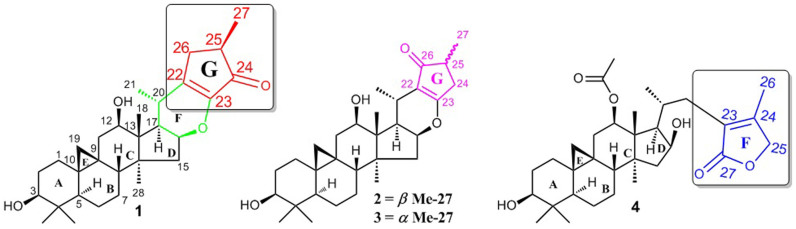
Chemical structures of compounds 1–4.

## References

[b1] Folkman J. Angiogenesis: an organizing principle for drug discovery? Nat Rev Drug Discov. 6, 273–286 (2007). 17396134 10.1038/nrd2115

[b2] Schoors S. *et al.* Partial and transient reduction of glycolysis by PFKFB3 blockade reduces pathological angiogenesis. Cell Metab. 19, 37–48 (2014). 24332967 10.1016/j.cmet.2013.11.008

[b3] Nian Y. *et al.* Triterpenes from the aerial parts of *Cimicifuga yunnanensis* and their antiproliferative effects on *p53^N236S^* mouse embryonic fibroblasts. J. Nat. Prod. 76, 896–902 (2013). 23621813 10.1021/np4000262

[b4] Nian Y. *et al.* Cytotoxic cycloartane triterpenes of the traditional chinese medicine “Shengma” (*Cimicifuga dahurica*). Planta Med. 79, 60–69 (2013). 23225366 10.1055/s-0032-1328019

[b5] Nian Y. *et al.* Cytotoxic cycloartane triterpenes from the roots of *Cimicifuga heracleifolia*. Tetrahedron. 68, 6521–6527 (2012).

[b6] Nian Y. *et al.* Cycloartane triterpenoids from the aerial parts of *Cimicifuga foetida* Linnaeus. Phytochemistry. 72, 1473–1481 (2011). 21565371 10.1016/j.phytochem.2011.03.022

[b7] Nian Y. *et al.* Cytotoxic chemical constituents from the roots of *Cimicifuga foetida*. J. Nat. Prod. 73, 93–98 (2010). 20121210 10.1021/np9003855

[b8] Nian Y. *et al.* Four new 9,19-cyclolanostane derivatives from the rhizomes of *Cimicifuga yunnanensis* Hsiao. Helv. Chim. Acta. 92, 112–120 (2009).

[b9] Lu L. *et al.* Studies on the constituents of *Cimicifuga foetida* collected in Guizhou province and their cytotoxic activities. Chem. Pharm. Bull. 60, 571–577 (2012). 22689393 10.1248/cpb.60.571

[b10] Lu L. *et al.* Five new triterpene bisglycosides with acyclic side chains from the rhizomes of *Cimicifuga foetida* L. Chem. Pharm. Bull. 58, 729–733 (2010). 20460805 10.1248/cpb.58.729

[b11] Lu L. *et al.* Trinor-cycloartane glycosides from the rhizomes of *Cimicifuga foetida*. Molecules. 14, 1578–1584 (2009). 19384286 10.3390/molecules14041578 PMC6254319

[b12] Sun L. R. *et al.* Two new triterpene glycosides with monomethyl malonate groups from the rhizome of *Cimifuga foetida* L. Molecules. 16, 5701–5708 (2011). 21730919 10.3390/molecules16075701 PMC6264661

[b13] Sun L. R. *et al.* New triterpene diglycosides from the rhizome of *Cimifuga foetida*. Molecules. 13, 1712–1721 (2008). 18794781 10.3390/molecules13081712 PMC6245406

[b14] Sun L. R. *et al.* Cimicifine A: a novel triterpene alkaloid from the rhizomes of *Cimicifuga foetida*. Helv. Chim. Acta. 90, 1313–1318 (2007).

[b15] Sun L. R. *et al.* Cimicifoetisides A and B, two cytotoxic cycloartane triterpenoid glycosides from the rhizomes of *Cimicifuga foetida*, inhibit proliferation of cancer cells. Beilstein J. of Org. Chem. 3, 1–6 (2007). 17266751 10.1186/1860-5397-3-3 PMC1803790

[b16] Wang H. Y. *et al.* Four new 9,19-Cyclolanostane triterpenes from the rhizomes of *Cimicifuga foetida* collected in Yulong. Chin J. Chem. 30, 1265–1268 (2012).

[b17] Li D. S., Nian Y., Sun Y. & Qiu M. H. Three new cycloartane (9,19-cyclolanostane) glycosides from *Cimicifuga foetida*. Helv. Chim. Acta. 94, 632–638 (2011).

[b18] Gao J. C. *et al.* Cytotoxic cycloartane triterpene saponins from *Actaea asiatica*. J. Nat. Prod. 69, 1500–150 (2006). 17067171 10.1021/np060113h

[b19] Li J. X. & Yu Z. Y. *Cimicifugae* rhizoma: from origins, bioactive constituents to clinical outcomes. Curr. Med. Chem. 13, 2927–2951 (2006). 17073639 10.2174/092986706778521869

[b20] Ali Z., Khan S. I., Ferreira D. & Khan I. A. Podocarpaside, a triterpenoid possessing a new backbone from *Actaea podocarpa*. Org. Lett. 8, 5529–5532 (2006). 17107064 10.1021/ol062212s

[b21] Dan C. *et al.* Cimicifugadine from *Cimicifuga foetida*, a new class of triterpene alklaoids with novel reactivity. Org. Lett. 9, 1813–1816 (2007). 17391044 10.1021/ol070542m

[b22] Li J. X. *et al.* Triterpenoids from *Cimicifuga* rhizoma, a novel class of inhibitors on bone resorption and ovariectomy-induced bone loss. Maturitas. 58, 59–69 (2007). 17658706 10.1016/j.maturitas.2007.06.001

[b23] Sakurai N. *et al.* Anti-AIDS agents. part 57: Actein, an anti-HIV principle from the rhizome of *Cimicifuga racemosa* (black cohosh), and the anti-HIV activity of related saponins. Bioorg. Med. Chem. Lett. 14, 1329–1332 (2004). 14980692 10.1016/j.bmcl.2003.12.035

[b24] Lee J. H. *et al.* Cycloartane-type triterpene glycosides from the rhizomes of *Cimicifuga heracleifolia* and their anticomplementary activity. Planta Med. 78, 1391–1394 (2012). 22753039 10.1055/s-0032-1314980

[b25] Findeis M. A. *et al.* Discovery of a novel pharmacological and structural class of gamma secretase modulators derived from the extract of *Actaea racemosa*. ACS Chem. Neurosci. 3, 941–951 (2012). 23205187 10.1021/cn3000857 PMC3509716

[b26] Fang Z. Z. *et al.* Cycloartane triterpenoids from *Cimicifuga yunnanensis* induce apoptosis of breast cancer cells (MCF7) *via* p53-dependent mitochondrial signaling pathway. Phytother Res. 25, 17–24 (2011). 20564500 10.1002/ptr.3222

[b27] Huang S. X. *et al.* Structural characterization of schintrilactone, a new class of nortriterpenoids from *Schisandra chinensis*. Org. Lett. 9, 4175–4178 (2007). 17880096 10.1021/ol701679n

[b28] Magnus P., Freund W. A., Moorhead E. J. & Rainey T. Formal synthesis of (±)-methyl rocaglate using an unprecedented acetyl bromide mediated nazarov reaction. J. Am. Chem. Soc. 134, 6140–6142 (2012). 22444715 10.1021/ja300900p

[b29] Tan H. B., Zheng C., Liu Z. & Wang D. Z. Biomimetic total syntheses of linderaspirone A and Bi-linderone and revisions of their biosynthetic pathways. Org. Lett. 9, 2192–2195 (2011). 21446662 10.1021/ol200418e

[b30] Chong C. R. & Jane P. A. The quest to overcome resistance to EGFR-targeted therapies in cancer. Nat Med. 19, 1389–1400 (2013). 24202392 10.1038/nm.3388 PMC4049336

[b31] Li W. H. & Vederas J. C. Drug discovery and natural products: end of an era or an endless frontier? Science. 325, 161–165 (2009). 19589993 10.1126/science.1168243

[b32] Neal C. P. *et al.* Clinical aspects of natural anti-Angiogenic drugs. Curr Drug Targets. 7, 371–383 (2006). 16555420 10.2174/138945006776054951

[b33] Pudhom K., Nuanyai T. & Matsubara K. Cytotoxic and anti-angiogenic properties of minor 3,4-*seco*-cycloartanes from *Gardenia sootepensis* exudate. Chem. Pharm. Bull. 60, 1538–1543 (2012). 23207634 10.1248/cpb.c12-00699

[b34] Frisch M. J. *et al.* Gaussian 09. [Gaussian, Inc. (ed.)] (Wallingford CT, USA, 2010).

[b35] Tähtinen P., Bagno A., Klika K. D. & Pihlaja K. Efficient implementation of the gauge-independent atomic orbital method for NMR chemical shift calculations. Modeling NMR parameters by DFT methods as an aid to the conformational analysis of cis-Fused 7a(8a)-Methyl Octa(hexa)hydrocyclopenta[*d*][1,3]oxazines and [3,1]benzoxazines. J. Am. Chem. Soc. 125, 4609–4618 (2003). 12683833 10.1021/ja021237t

[b36] Ditchfield R. Self-consistent perturbation theory of diamagnetism: I. A gauge-Invariant LCAO (Linear Combination of Atomic Orbitals) method for NMR chemical shifts. Mol. Phys. 27, 789–807 (1974).

[b37] Rohlfing C. M., Allen L. C. & Ditchfield R. Proton and ^13^C chemical shifts: comparison between theory and experiment. Chem. Phys. 87, 9–15 (1984).

[b38] Wolinski K., Hinton J. F. & Pulay P. Efficient implementation of the gauge-independent atomic orbital method for NMR chemical shift calculations. J. Am. Chem. Soc. 112, 8251–8260 (1990).

[b39] Miertus S., Scrocc E. & Tomasi J. Electrostatic interaction of a solute with a continuum. A direct utilization of *ab initio* molecular potentials for the prevision of solvent effects. Chem. Phys. 55, 117–129 (1981).

[b40] Miertus S. & Tomasi J. Approximate evaluations of the electrostatic free energy and internal energy changes in solution processes. Chem. Phys. 65, 239–241 (1982).

[b41] Cossi M., Barone V., Cammi R. & Tomasi J. *Ab initio* study of solvated molecules: a new implementation of the polarizable continuum model. Chem. Phys. Lett. 255, 327–335 (1996).

