# Supplementary material for: New Anti-angiogenic Leading Structure Discovered in the Fruit of Cimicifuga yunnanensis
Source: Sci Rep. 2015 Mar 12;5:9026. doi: 10.1038/srep09026 (PMC4356973; doi:10.1038/srep09026)

**Supplementary Information**

New Anti-angiogenic Leading Structure Discovered in the Fruit of *Cimicifuga yunnanensis*

Yin Nian,^1^ Jing Yang,^1^ Tong-Yang Liu,^2^ Ying Luo,^2^ Ji-Hong Zhang,^2*^ and Ming-Hua Qiu^1*^

^1^State Key Laboratory of Phytochemistry and Plant Resources in West China, Kunming Institute of Botany, Chinese Academy of Sciences, Kunming 650201, P. R. China ^2^Laboratory of Molecular Genetics of Aging & Tumor, Faculty of Medicine, Kunming University of Science & Technology, Kunming, 650500, P. R. China

**^*^**Author to whom correspondence should be addressed;

E-Mail: zhjihong2000@126.com (Z.J.H.); Tel.: +86-871- 65920753; fax: +86-871- 65920753 and mhchiu@mail.kib.ac.cn (Q.M.H.); Tel.: +86-871-65223327; fax: +86-871-65223325.

Content

[1. ^1^H and ^13^C NMR assignments of cimyunnins A-D (**1-4**) 1](#__RefHeading___Toc406685461)

[2. Scheme 1. Key Plausible Biogenetic Routs of Compounds **1-4** 4](#__RefHeading___Toc406685462)

[3*.* Figure S1. X-ray crystal structure of the epimers of **2** and **3**. 4](#__RefHeading___Toc406685463)

[4. Computational data of **1** 5](#__RefHeading___Toc406685464)

[5. Computational data of **4** 21](#__RefHeading___Toc406685465)

[6. NMR, MS, IR, CD, and UV spectra of cimyunnins A-D (**1-4**) 31](#__RefHeading___Toc406685466)

[7. Full length gel and blots of VEGFR2 signalling pathway 93](#__RefHeading___Toc406685467)

## 1. ^1^H and ^13^C NMR assignments of cimyunnins A-D (1-4)

**Table S1**. NMR Data of Compound **1** (*δ* in ppm, *J* in Hz)

| no. | *δ*_H_^b^ | *δ*_C_^b^ | no. | *δ*_H_^b^ | *δ*_C_^b^ |
| --- | --- | --- | --- | --- | --- |
| 1 | 1.53 m  1.20^a^ | 32.8 t | 16 | 4.52 m | 76.9 d |
| 2 | 1.93 m  1.81 m | 30.2 t | 17 | 2.27 brd (6.6) | 52.9 d |
| 3 | 3.50 dd (11.3, 3.7) | 78.2 d | 18 | 1.23 s | 12.6 q |
| 4 |  | 41.5 s | 19 | 0.67 d (3.4)  0.26 d (3.8) | 31.0 t |
| 5 | 1.25^a^ | 47.5 d | 20 | 3.30 m | 29.0 d |
| 6 | 1.54 m  0.83 m | 21.2 t | 21 | 1.26 d (7.4) | 22.6 d |
| 7 | 1.28^a^  0.99 m | 26.4 t | 22 |  | 148.9 s |
| 8 | 1.65 dd (11.8, 5.2) | 47.0 d | 23 |  | 149.3 s |
| 9 |  | 21.4 s | 24 |  | 203.5 s |
| 10 |  | 27.9 s | 25 | 2.32 m | 38.7 d |
| 11 | 2.62 dd (15.4, 9.0)  1.42 dd (13.0, 3.5) | 41.3 t | 26 | 2.67 dd (17.1, 7.3)  1.73 brd (17.1) | 33.3 t |
| 12 | 4.23 m | 73.4 d | 27 | 1.13 d (7.3) | 17.6 q |
| 13 |  | 50.8s | 28 | 0.89 s | 21.5 q |
| 14 |  | 48.5 s | 29 | 1.05 s | 15.3 q |
| 15 | 2.06 dd (13.8, 7.4)  1.96 brd (14.5) | 46.5 t | 30 | 1.21 s | 26.7 q |
| *^a^*Signals overlapped.  *^b^* NMR data was Recorded at 600 MHz in Pyridine-*d*_5_. | | | | | |

**Table S2**. ^1^H and ^13^C NMR Data of Compounds **2** and **3** (*δ* in ppm, *J* in Hz)

| no | 2^b,d^ | | 3^b,d^ | | no | 2^b,d^ | | 3^b,d^ | |
| --- | --- | --- | --- | --- | --- | --- | --- | --- | --- |
|  | H C | | H C | |  | H C | | H C | |
| 1 | 1.45 m  1.12 m | 31.53 t^c^ | 1.45 m  1.12 m | 31.56 t^c^ | 16 | 4.72 m | 78.93 d^c^ | 4.72 m | 78.93 d^c^ |
| 2 | 1.58 m  1.45 m | 30.17 t^c^ | 1.58 m  1.45 m | 30.17 t^c^ | 17 | 2.06 m | 49.73 d^c^ | 2.06 m | 49.73 d^c^ |
| 3 | 3.04 m | 76.52 d^c^ | 3.04 m | 76.52 d^c^ | 18 | 0.76 s | 11.34 q^c^ | 0.76 s | 11.36 q^c^ |
| 4 |  | 40.03 s^c^ |  | 40.03 s^c^ | 19 | 0.56 d (3.9)  0.23 d (3.6) | 29.81 t^c^ | 0.56 d (3.9)  0.23 d (3.6) | 29.95 t^c^ |
| 5 | 1.16 dd (12.4, 3.9) | 46.42 d^c^ | 1.16 dd (12.4, 3.9) | 46.38 d^c^ | 20 | 2.83 m | 20.80 d^c^ | 2.83 m | 20.83 d^c^ |
| 6 | 1.48 m  0.76 | 20.06 t | 1.48 m  0.76 | 20.10 t^c^ | 21 | 1.05 d (7.0) | 22.53 q^c^ | 1.06 d (7.0) | 22.55 q^c^ |
| 7 | 1.30 m  0.97 m | 25.26 t^c^ | 1.30 m  0.97 m | 25.32 t^c^ | 22 |  | 116.38 s |  | 116.54 s |
| 8 | 1.57 m | 45.76 d^c^ | 1.57 m | 45.92 d^c^ | 23 |  | 180.20 s |  | 180.15 s |
| 9 |  | 20.32 s^c^ |  | 20.32 s^c^ | 24 | 2.68 m  1.99 t (18.7) | 34.52 t | 2.65 m  1.99 t (18.7) | 34.68 t |
| 10 |  | 26.69 s^c^ |  | 26.69 s^c^ | 25 | 2.28 m | 38.04 d | 2.28 m | 37.94 d |
| 11 | 2.30 m  0.96 m | 39.54 t^c^ | 2.30 m  0.96 m | 39.61 t^c^ | 26 |  | 205.13 s |  | 205.02 s |
| 12 | 3.74 m | 71.60 d^c^ | 3.74 m | 71.62 d^c^ | 27 | 1.03 d (7.4) | 17.05 q | 1.00 d (7.4) | 16.77 q |
| 13 |  | 49.31s^c^ |  | 49.33 s^c^ | 28 | 0.82 s | 20.26 q^c^ | 0.82 s | 20.25 q^c^ |
| 14 |  | 47.66 s^c^ |  | 47.66 s^c^ | 29 | 0.68 s | 14.34 q^c^ | 0.68 s | 14.34 q^c^ |
| 15 | 2.08 m  1.71 brd (14.2) | 45.38 t^c^ | 2.08 m  1.71 brd (14.2) | 45.42 t^c^ | 30 | 0.86 s | 25.77 q^c^ | 0.86 s | 25.77 q^c^ |
| *^a^*Signals overlapped;  *^b^*NMR data for ^1^H was recorded at 500 MHz in DMSO-*d*_6_, other NMR data was recorded at 150 MHz in DMSO-*d*_6_;^c^Data is exchangeable between **2** and **3**. ^d^Data recorded in Pyridine-*d*_5_ can not assign the NMR data of **2** and **3** due to signals overlapped. Thus, DMSO-*d*_6_ was used as the solvent, and can assign the signals for the key structural units (ring G) by ROESY and HMBC spectra. All of these spectroscopic data were showed below. | | | | | | | | | |

**Table S3.** NMR Data of Compound **4** (*δ* in ppm, *J* in Hz)

| no. | *δ*_H_^b^ | *δ*_C_^b^ | no. | *δ*_H_^b^ | *δ*_C_^b^ |
| --- | --- | --- | --- | --- | --- |
| 1 | 1.35 m  0.93*^a^* | 32.8 t | 16 | 4.67 m | 72.1 d |
| 2 | 1.80 m  1.69 m | 31.7 t | 17^c^ | 1.86 dd (>10, 5.6) | 58.6 d |
| 3 | 3.39 dd (11.5, 4.2) | 78.3 d | 18 | 1.44 s | 14.2 q |
| 4 |  | 41.5 s | 19 | 0.46 d (3.7)  0.27 d (4.1) | 30.3 t |
| 5 | 1.12 m | 47.7 d | 20 | 2.24 m | 31.2 d |
| 6 | 1.43 *^a^*  0.61 m | 21.7 t | 21 | 0.86 d (6.8) | 18.4 q |
| 7 | 1.19 m  0.86 *^a^* | 26.5 t | 22 | 2.71 brd (11.7)  1.93 dd (13.2, 10.6) | 31.2 t |
| 8 | 1.53 dd (12.5, 4.6) | 47.5 d | 23 |  | 127.1 s |
| 9 |  | 19.9 s | 24 |  | 159.7 s |
| 10 |  | 26.6 s | 25 | 4.59 d (17.0)  4.55 d (17.0) | 73.6 t |
| 11 | 2.73 dd (16.0, 8.1)  1.06 brd (16.4) | 38.1 t | 26 | 1.84 s | 12.8 q |
| 12 | 5.09^a^ | 77.1 d | 27 |  | 177.3 s |
| 13 |  | 50.1 s | 28 | 0.85 s | 21.0 q |
| 14 |  | 48.8 s | 29 | 0.95 s | 15.4 q |
| 15 | 2.06 m  1.77 m | 48.7 t | 30 | 1.09 s | 26.6 q |
|  |  |  | AcO-12 | 2.03 s | 22.2 q  171.2 s |
| *^a^*Signals overlapped.  *^b^*NMR data was Recorded at 600 MHz in Pyridine-*d*_5_. ^c^The signal for H-17 should be doublet-doublet splitted by H-20 and H-16. Although the signal for H-17 was partly overlapped, the coupling constant from H-16 was 5.6 Hz. Thus, the coupling constant from H-17 should be bigger than 10 Hz. | | | | | |

# 2. Scheme 1. Key Plausible Biogenetic Routs of Compounds 1-4

# 3*.* Figure S1. X-ray crystal structure of the epimers of 2 and 3.

## 4. Computational data of 1

**Figure S2.** Optimized geometries of predominant conformers for compound **1A** at the B3LYP/6-31G(d,p) level in the gas phase

**1A**


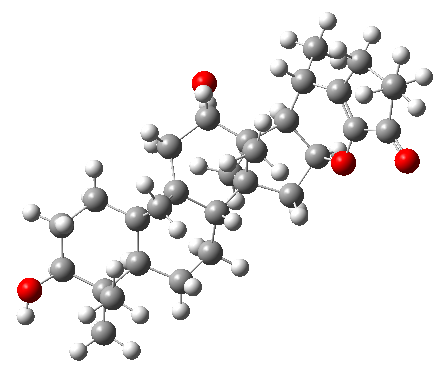

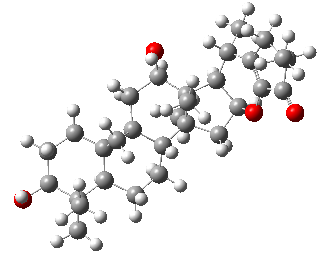


**1a 1b**


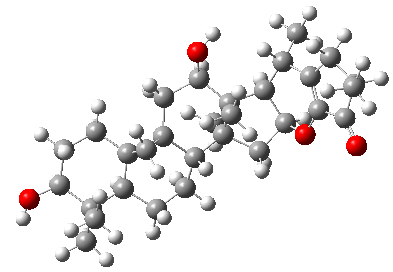

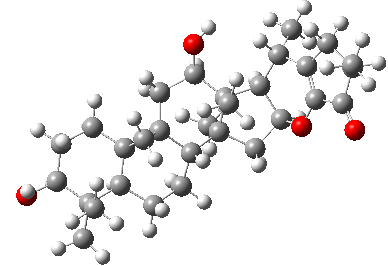


**1c 1d**


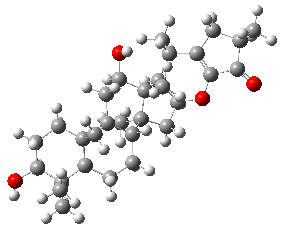

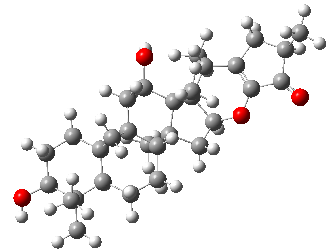


**1e 1f**


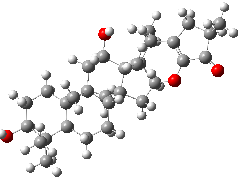

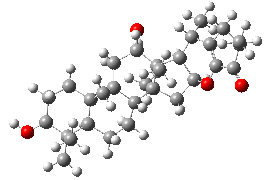


**1g 1h**


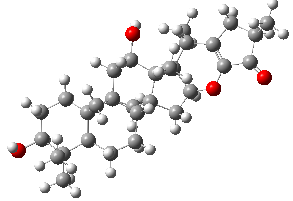

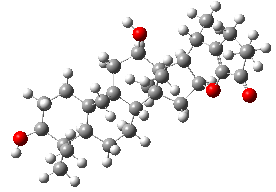


**1i 1j**


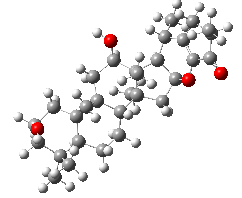

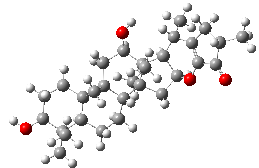


1k 1l


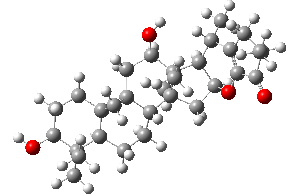


**1m**

**Table S4.** Important thermodynamic parameters (a.u.) and Boltzmann distributions of the optimized compound **1A** at B3LYP/6-31G(d,p) level in the gas phase

| Conformations | E+ZPE | G | % |
| --- | --- | --- | --- |
| **1a** | -1469.899906 | -1469.959275 | 14.3 |
| **1b** | -1469.900581 | -1469.959954 | 29.3 |
| **1c** | -1469.897659 | -1469.957689 | 2.7 |
| **1d** | -1469.898398 | -1469.958361 | 5.4 |
| **1e** | -1469.897485 | -1469.957564 | 2.3 |
| **1f** | -1469.895527 | -1469.956204 | 0.6 |
| **1g** | -1469.897689 | -1469.957697 | 2.7 |
| **1h** | -1469.900226 | -1469.959672 | 21.8 |
| **1i** | -1469.895894 | -1469.956668 | 0.9 |
| **1j** | -1469.899154 | -1469.958816 | 8.8 |
| **1k** | -1469.891377 | -1469.950940 | 0.0 |
| **1l** | -1469.898487 | -1469.958625 | 7.2 |
| **1m** | -1469.897990 | -1469.958096 | 4.1 |

E+ZPE, G: total energy with zero point energy (ZPE) and Gibbs free energy in the gas phase at B3LYP/6-31G(d,p) level, %: Boltzmann distributions, using the relative Gibbs free energies as weighting factors。

**Table S5.** Optimized Z-Matrixes of compound **1A** in the Gas Phase (Å) at B3LYP/6-31G(d,p) level.

| **1a** | | | | **1b** | | | |
| --- | --- | --- | --- | --- | --- | --- | --- |
| C | 5.366044 | 1.262152 | -1.37517 | C | 5.372419 | 1.266822 | -1.35893 |
| C | 6.165412 | 0.27193 | -0.53373 | C | 6.173653 | 0.25901 | -0.53108 |
| C | 5.365544 | -1.02153 | -0.17698 | C | 5.366609 | -1.02578 | -0.16332 |
| C | 4.031687 | -0.56156 | 0.509421 | C | 4.032236 | -0.5587 | 0.514876 |
| C | 3.216286 | 0.408211 | -0.36446 | C | 3.219458 | 0.412437 | -0.35896 |
| C | 4.042512 | 1.648063 | -0.70178 | C | 4.047127 | 1.653419 | -0.68738 |
| C | 3.175557 | -1.69525 | 1.094723 | C | 3.175975 | -1.68919 | 1.104965 |
| C | 1.945825 | -1.1149 | 1.799235 | C | 1.946603 | -1.10523 | 1.806408 |
| C | 0.980406 | -0.46472 | 0.796545 | C | 0.982528 | -0.45997 | 0.798726 |
| C | 1.693871 | 0.536897 | -0.17115 | C | 1.696907 | 0.539212 | -0.17114 |
| C | -0.28423 | 0.160033 | 1.438281 | C | -0.28349 | 0.166438 | 1.436237 |
| C | -1.14502 | 0.845184 | 0.310715 | C | -1.14357 | 0.846819 | 0.305359 |
| C | -0.44346 | 2.151591 | -0.10574 | C | -0.4424 | 2.152077 | -0.11527 |
| C | 1.097159 | 1.957348 | -0.30541 | C | 1.098939 | 1.958735 | -0.31071 |
| C | -2.51232 | 1.023411 | 1.031456 | C | -2.51168 | 1.026939 | 1.02407 |
| C | -2.70925 | -0.32777 | 1.771868 | C | -2.70831 | -0.3216 | 1.769455 |
| O | -3.40934 | -1.34025 | 1.00445 | O | -3.40724 | -1.33715 | 1.005154 |
| C | -1.28499 | -0.87234 | 2.042042 | C | -1.28407 | -0.86442 | 2.042747 |
| C | -3.74089 | 1.529384 | 0.223225 | C | -3.73997 | 1.529289 | 0.212947 |
| C | -4.61537 | 0.38502 | -0.19221 | C | -4.616 | 0.383476 | -0.19526 |
| C | -4.4007 | -0.88538 | 0.206248 | C | -4.40051 | -0.88541 | 0.207531 |
| C | -5.36465 | -1.82158 | -0.40896 | C | -5.36617 | -1.82389 | -0.40153 |
| C | -6.34726 | -0.96745 | -1.22874 | C | -6.35193 | -0.97255 | -1.22038 |
| C | -5.79688 | 0.477923 | -1.12977 | C | -5.8002 | 0.472877 | -1.12984 |
| C | 2.268637 | -0.09735 | -1.42801 | C | 2.276799 | -0.09436 | -1.42671 |
| C | 5.138371 | -1.87953 | -1.43624 | C | 5.133694 | -1.89712 | -1.41467 |
| C | 6.211365 | -1.8401 | 0.821839 | C | 6.219789 | -1.83336 | 0.836605 |
| C | -1.31651 | -0.06076 | -0.93522 | C | -1.31338 | -0.0641 | -0.93715 |
| C | -4.53617 | 2.59083 | 1.016103 | C | -4.53338 | 2.596996 | 0.999232 |
| O | 7.36723 | 0.00488 | -1.25796 | O | 7.42427 | -0.03565 | -1.1557 |
| C | -6.51869 | -1.51004 | -2.6506 | C | -6.5309 | -1.52097 | -2.63901 |
| H | -2.34995 | 1.760739 | 1.824073 | H | -2.35035 | 1.767269 | 1.814048 |
| O | -5.38461 | -3.03447 | -0.30865 | O | -5.38519 | -3.03646 | -0.29756 |
| O | -1.04632 | 2.778027 | -1.24492 | O | -1.04317 | 2.773202 | -1.25841 |
| H | 4.351318 | 0.037318 | 1.38023 | H | 4.354979 | 0.040524 | 1.383939 |
| H | 0.615717 | -1.29155 | 0.175115 | H | 0.619687 | -1.28932 | 0.179505 |
| H | 5.173639 | 0.821549 | -2.36024 | H | 5.163958 | 0.841144 | -2.351 |
| H | 5.984634 | 2.149819 | -1.54598 | H | 5.994096 | 2.153749 | -1.52427 |
| H | 6.415415 | 0.766313 | 0.422289 | H | 6.454887 | 0.741222 | 0.415114 |
| H | 3.493663 | 2.329871 | -1.35605 | H | 3.501576 | 2.341774 | -1.33766 |
| H | 4.252815 | 2.206639 | 0.222324 | H | 4.259472 | 2.204337 | 0.240498 |
| H | 2.862913 | -2.40104 | 0.313958 | H | 2.859745 | -2.39792 | 0.328252 |
| H | 3.766814 | -2.27213 | 1.812085 | H | 3.771313 | -2.26297 | 1.821284 |
| H | 1.417566 | -1.903 | 2.350032 | H | 1.417238 | -1.89019 | 2.360524 |
| H | 2.289242 | -0.38582 | 2.543221 | H | 2.289098 | -0.37228 | 2.546864 |
| H | -0.59316 | 2.890971 | 0.68641 | H | -0.59425 | 2.894687 | 0.673407 |
| H | 1.611648 | 2.631442 | 0.389544 | H | 1.610896 | 2.635047 | 0.383896 |
| H | 1.325296 | 2.351877 | -1.29944 | H | 1.328825 | 2.351079 | -1.30526 |
| H | -3.2924 | -0.18606 | 2.689817 | H | -3.29226 | -0.17669 | 2.686387 |
| H | -1.11071 | -1.03207 | 3.109903 | H | -1.11056 | -1.02025 | 3.111265 |
| H | -1.20446 | -1.8492 | 1.556819 | H | -1.20253 | -1.84299 | 1.561144 |
| H | -3.36158 | 2.029956 | -0.67769 | H | -3.36051 | 2.023746 | -0.69132 |
| H | -7.31436 | -1.03244 | -0.71259 | H | -7.31649 | -1.03456 | -0.69909 |
| H | -6.55511 | 1.184082 | -0.76802 | H | -6.55684 | 1.181324 | -0.76928 |
| H | -5.47946 | 0.853408 | -2.11326 | H | -5.48543 | 0.843337 | -2.11609 |
| H | 2.134568 | -1.16806 | -1.55687 | H | 2.146922 | -1.16559 | -1.55619 |
| H | 2.221101 | 0.462104 | -2.36083 | H | 2.228066 | 0.465768 | -2.35918 |
| H | 4.662961 | -2.83278 | -1.1835 | H | 4.573193 | -2.80295 | -1.1656 |
| H | 6.098811 | -2.09489 | -1.91166 | H | 6.093049 | -2.22641 | -1.82877 |
| H | 4.509469 | -1.37922 | -2.17457 | H | 4.581865 | -1.37591 | -2.20072 |
| H | 5.743202 | -2.79754 | 1.064498 | H | 5.802377 | -2.82698 | 1.024628 |
| H | 6.369706 | -1.29448 | 1.760074 | H | 6.302385 | -1.31414 | 1.798998 |
| H | 7.193804 | -2.07895 | 0.398259 | H | 7.229507 | -1.95966 | 0.43764 |
| H | -2.08886 | 0.346274 | -1.59369 | H | -1.63003 | -1.0724 | -0.66775 |
| H | -0.39409 | -0.14187 | -1.5154 | H | -2.0856 | 0.339519 | -1.59791 |
| H | -1.63356 | -1.06994 | -0.66965 | H | -0.3904 | -0.14704 | -1.51627 |
| H | -5.40363 | 2.945047 | 0.451234 | H | -5.40022 | 2.949295 | 0.432227 |
| H | -3.90374 | 3.4577 | 1.233205 | H | -3.89938 | 3.464041 | 1.210879 |
| H | -4.89839 | 2.183465 | 1.966544 | H | -4.89623 | 2.19621 | 1.952212 |
| H | 7.982516 | -0.44417 | -0.66532 | H | 7.236092 | -0.32235 | -2.0593 |
| H | -5.58471 | -1.42293 | -3.21686 | H | -6.80579 | -2.57838 | -2.59815 |
| H | -6.79325 | -2.56775 | -2.61564 | H | -7.31062 | -0.97594 | -3.18031 |
| H | -7.29594 | -0.9631 | -3.19354 | H | -5.59976 | -1.43678 | -3.21037 |
| H | -0.93214 | 2.183956 | -1.99887 | H | -0.93133 | 2.173799 | -2.00847 |
| C | 0.119861 | 1.14076 | 2.573437 | C | 0.118514 | 1.151497 | 2.568527 |
| H | -0.73006 | 1.702192 | 2.969455 | H | 0.531361 | 0.589096 | 3.409865 |
| H | 0.533385 | 0.575141 | 3.412339 | H | 0.879183 | 1.872961 | 2.262914 |
| H | 0.880559 | 1.862747 | 2.269083 | H | -0.73225 | 1.713783 | 2.961498 |
| **1c** | | | | **1d** | | | |
| C | 5.375895 | 1.284747 | -1.34334 | C | 5.382857 | 1.289612 | -1.32437 |
| C | 6.172385 | 0.278482 | -0.51832 | C | 6.181135 | 0.263392 | -0.51634 |
| C | 5.373414 | -1.02419 | -0.19435 | C | 5.373355 | -1.0303 | -0.1823 |
| C | 4.035615 | -0.58178 | 0.495759 | C | 4.036542 | -0.5795 | 0.50223 |
| C | 3.223609 | 0.406095 | -0.36013 | C | 3.227399 | 0.411316 | -0.35194 |
| C | 4.04823 | 1.654019 | -0.6688 | C | 4.054389 | 1.659903 | -0.65008 |
| C | 3.177417 | -1.7302 | 1.048435 | C | 3.177498 | -1.72414 | 1.060144 |
| C | 1.943323 | -1.16803 | 1.760316 | C | 1.944422 | -1.15749 | 1.769634 |
| C | 0.982215 | -0.49116 | 0.770475 | C | 0.984828 | -0.48536 | 0.774643 |
| C | 1.699609 | 0.529923 | -0.17109 | C | 1.703424 | 0.533835 | -0.16826 |
| C | -0.2813 | 0.120843 | 1.425492 | C | -0.28075 | 0.127881 | 1.424553 |
| C | -1.13474 | 0.848239 | 0.314862 | C | -1.13271 | 0.850116 | 0.309546 |
| C | -0.41453 | 2.165458 | -0.02918 | C | -0.4132 | 2.166752 | -0.03898 |
| C | 1.105783 | 1.953718 | -0.29306 | C | 1.10911 | 1.957092 | -0.29299 |
| C | -2.50886 | 0.998801 | 1.034251 | C | -2.50821 | 1.002528 | 1.025929 |
| C | -2.70556 | -0.37038 | 1.745584 | C | -2.70488 | -0.36393 | 1.742621 |
| O | -3.41748 | -1.36681 | 0.969192 | O | -3.41548 | -1.36365 | 0.969392 |
| C | -1.28382 | -0.92774 | 1.991688 | C | -1.28325 | -0.91964 | 1.992584 |
| C | -3.7395 | 1.511551 | 0.228284 | C | -3.73811 | 1.511302 | 0.216045 |
| C | -4.63163 | 0.378889 | -0.18263 | C | -4.63242 | 0.377277 | -0.18646 |
| C | -4.41586 | -0.89745 | 0.192703 | C | -4.41592 | -0.89753 | 0.193625 |
| C | -5.39521 | -1.81975 | -0.42225 | C | -5.39741 | -1.82217 | -0.41441 |
| C | -6.37856 | -0.94959 | -1.22364 | C | -6.38384 | -0.95494 | -1.21516 |
| C | -5.82246 | 0.492479 | -1.10591 | C | -5.82636 | 0.487314 | -1.10622 |
| C | 2.281004 | -0.08007 | -1.43608 | C | 2.28946 | -0.07412 | -1.43272 |
| C | 5.153063 | -1.85412 | -1.4735 | C | 5.144421 | -1.87075 | -1.45539 |
| C | 6.215728 | -1.86363 | 0.790042 | C | 6.222929 | -1.86196 | 0.800632 |
| C | -1.30123 | 0.003206 | -0.96998 | C | -1.29645 | -0.0008 | -0.9718 |
| C | -4.51761 | 2.593144 | 1.010299 | C | -4.51374 | 2.600785 | 0.989467 |
| O | 7.379839 | 0.029936 | -1.24019 | O | 7.434906 | -0.01584 | -1.14221 |
| C | -6.55595 | -1.46912 | -2.65369 | C | -6.56855 | -1.48068 | -2.64195 |
| H | -2.36079 | 1.717037 | 1.848293 | H | -2.36191 | 1.724137 | 1.837247 |
| O | -5.42401 | -3.03272 | -0.33253 | O | -5.42562 | -3.0348 | -0.32033 |
| O | -0.94678 | 2.825817 | -1.19102 | O | -0.93996 | 2.818711 | -1.20789 |
| H | 4.350199 | -0.00412 | 1.382756 | H | 4.356217 | -0.00245 | 1.387453 |
| H | 0.615627 | -1.30102 | 0.128192 | H | 0.620756 | -1.29772 | 0.134049 |
| H | 5.189023 | 0.864596 | -2.33827 | H | 5.178477 | 0.886561 | -2.32657 |
| H | 5.994093 | 2.176683 | -1.4923 | H | 6.005031 | 2.180227 | -1.46686 |
| H | 6.415295 | 0.752868 | 0.449767 | H | 6.457816 | 0.723725 | 0.442144 |
| H | 3.499482 | 2.346708 | -1.31163 | H | 3.509248 | 2.360631 | -1.28739 |
| H | 4.253658 | 2.19474 | 0.267245 | H | 4.262922 | 2.191184 | 0.290317 |
| H | 2.869333 | -2.4174 | 0.249673 | H | 2.865009 | -2.41393 | 0.265261 |
| H | 3.765122 | -2.32369 | 1.75522 | H | 3.76905 | -2.31518 | 1.76562 |
| H | 1.412834 | -1.97029 | 2.288312 | H | 1.412699 | -1.95634 | 2.301446 |
| H | 2.28443 | -0.45893 | 2.524459 | H | 2.285249 | -0.44434 | 2.530021 |
| H | -0.52231 | 2.846565 | 0.825332 | H | -0.52669 | 2.852889 | 0.810794 |
| H | 1.655947 | 2.634927 | 0.366691 | H | 1.654047 | 2.637837 | 0.371513 |
| H | 1.275559 | 2.324039 | -1.3066 | H | 1.285331 | 2.329039 | -1.30486 |
| H | -3.28044 | -0.24337 | 2.671014 | H | -3.28088 | -0.23331 | 2.666846 |
| H | -1.10848 | -1.13137 | 3.051929 | H | -1.10927 | -1.11978 | 3.053676 |
| H | -1.20893 | -1.88363 | 1.465877 | H | -1.20694 | -1.8772 | 1.470008 |
| H | -3.36964 | 1.982546 | -0.69369 | H | -3.36778 | 1.9743 | -0.70988 |
| H | -7.34404 | -1.0195 | -0.7053 | H | -7.34682 | -1.02184 | -0.69179 |
| H | -6.57613 | 1.193456 | -0.72469 | H | -6.57821 | 1.190615 | -0.72577 |
| H | -5.51357 | 0.882798 | -2.08635 | H | -5.52067 | 0.872669 | -2.08965 |
| H | 2.148536 | -1.14841 | -1.58493 | H | 2.160691 | -1.14274 | -1.58341 |
| H | 2.22958 | 0.496956 | -2.35733 | H | 2.237019 | 0.50466 | -2.35291 |
| H | 4.676575 | -2.81266 | -1.24382 | H | 4.577695 | -2.77927 | -1.2314 |
| H | 6.115819 | -2.05868 | -1.94908 | H | 6.104751 | -2.19478 | -1.87138 |
| H | 4.527125 | -1.33773 | -2.20306 | H | 4.599899 | -1.32808 | -2.2318 |
| H | 5.747997 | -2.82715 | 1.008448 | H | 5.803672 | -2.85913 | 0.964162 |
| H | 6.367861 | -1.33899 | 1.741219 | H | 6.303221 | -1.36533 | 1.775107 |
| H | 7.201025 | -2.09112 | 0.366683 | H | 7.23369 | -1.98008 | 0.40174 |
| H | -2.01085 | 0.489268 | -1.64191 | H | -2.00607 | 0.481007 | -1.64677 |
| H | -0.36541 | -0.08661 | -1.52228 | H | -0.35987 | -0.09162 | -1.52269 |
| H | -1.67349 | -0.99952 | -0.75346 | H | -1.66753 | -1.00307 | -0.75116 |
| H | -5.37742 | 2.960115 | 0.441978 | H | -5.37219 | 2.965932 | 0.417938 |
| H | -3.87378 | 3.451354 | 1.233793 | H | -3.86763 | 3.458807 | 1.206948 |
| H | -4.88879 | 2.195823 | 1.961016 | H | -4.8865 | 2.211586 | 1.942907 |
| H | 7.989744 | -0.43568 | -0.65479 | H | 7.250147 | -0.27968 | -2.05346 |
| H | -5.62308 | -1.37698 | -3.22073 | H | -6.84841 | -2.53724 | -2.61675 |
| H | -6.83535 | -2.52593 | -2.63462 | H | -7.3473 | -0.92372 | -3.17237 |
| H | -7.3324 | -0.9102 | -3.18542 | H | -5.6384 | -1.39162 | -3.21396 |
| H | -1.71822 | 3.336266 | -0.91638 | H | -1.71803 | 3.323872 | -0.94221 |
| C | 0.126143 | 1.062827 | 2.592192 | C | 0.123006 | 1.074279 | 2.589075 |
| H | 0.900561 | 1.781167 | 2.312998 | H | 0.896542 | 1.793299 | 2.309394 |
| H | -0.71898 | 1.625663 | 2.997266 | H | -0.72386 | 1.636693 | 2.991101 |
| H | 0.524403 | 0.468219 | 3.41827 | H | 0.521049 | 0.482852 | 3.417491 |
| **1e** | | | | **1f** | | | |
| C | 5.376037 | 1.777901 | -0.15586 | C | 5.385354 | 1.770644 | -0.1541 |
| C | 5.957565 | 0.615335 | 0.635849 | C | 5.964559 | 0.607311 | 0.637898 |
| C | 5.462393 | -0.77078 | 0.120107 | C | 5.467194 | -0.77777 | 0.121915 |
| C | 3.889301 | -0.77637 | 0.161394 | C | 3.89397 | -0.78074 | 0.162149 |
| C | 3.256064 | 0.435539 | -0.55833 | C | 3.262132 | 0.432657 | -0.55642 |
| C | 3.845668 | 1.76514 | -0.09929 | C | 3.85504 | 1.7607 | -0.09688 |
| C | 3.283955 | -2.0978 | -0.338 | C | 3.287457 | -2.10084 | -0.33902 |
| C | 1.764363 | -2.1598 | -0.14227 | C | 1.768301 | -2.16076 | -0.14132 |
| C | 0.942468 | -0.90807 | -0.57462 | C | 0.947879 | -0.90861 | -0.57527 |
| C | 1.769782 | 0.352208 | -0.9526 | C | 1.774729 | 0.353195 | -0.95063 |
| C | -0.10983 | -0.52335 | 0.511973 | C | -0.11059 | -0.52757 | 0.506412 |
| C | -1.09823 | 0.576003 | -0.04062 | C | -1.08709 | 0.582285 | -0.04789 |
| C | -0.29189 | 1.876762 | -0.19419 | C | -0.26736 | 1.877779 | -0.18087 |
| C | 0.923174 | 1.640872 | -1.11805 | C | 0.933388 | 1.644965 | -1.11386 |
| C | -2.20153 | 0.568757 | 1.056621 | C | -2.20521 | 0.5706 | 1.035663 |
| C | -2.41531 | -0.94948 | 1.33602 | C | -2.42043 | -0.94947 | 1.316219 |
| O | -3.46636 | -1.57319 | 0.555918 | O | -3.4807 | -1.57623 | 0.553191 |
| C | -1.0907 | -1.65098 | 0.940085 | C | -1.10152 | -1.65359 | 0.909057 |
| C | -3.49689 | 1.405771 | 0.845894 | C | -3.49953 | 1.406302 | 0.798237 |
| C | -4.63935 | 0.539084 | 0.410123 | C | -4.65127 | 0.534832 | 0.396652 |
| C | -4.54782 | -0.8022 | 0.306704 | C | -4.56221 | -0.8071 | 0.308444 |
| C | -5.79572 | -1.39804 | -0.21449 | C | -5.81932 | -1.40705 | -0.18841 |
| C | -6.80406 | -0.24634 | -0.36608 | C | -6.82409 | -0.25419 | -0.35173 |
| C | -6.00276 | 1.033087 | -0.01521 | C | -6.01726 | 1.027547 | -0.02195 |
| C | 2.856778 | 0.261664 | -2.00776 | C | 2.861149 | 0.261666 | -2.0059 |
| C | 6.02462 | -1.0429 | -1.29037 | C | 6.029211 | -1.05084 | -1.28846 |
| C | 6.011058 | -1.84735 | 1.081988 | C | 6.013661 | -1.85529 | 1.084072 |
| C | -1.73427 | 0.187477 | -1.39994 | C | -1.71268 | 0.217213 | -1.41699 |
| C | -3.84961 | 2.21738 | 2.112286 | C | -3.84549 | 2.270935 | 2.031034 |
| O | 7.378398 | 0.753936 | 0.588228 | O | 7.385779 | 0.743716 | 0.591802 |
| C | -7.46504 | -0.25141 | -1.74753 | C | -7.48764 | -0.27561 | -1.73211 |
| H | -1.73998 | 0.965893 | 1.964562 | H | -1.76078 | 0.966432 | 1.953967 |
| O | -5.99967 | -2.56641 | -0.48811 | O | -6.03035 | -2.57939 | -0.43645 |
| O | -1.05654 | 2.988183 | -0.67597 | O | -1.00197 | 2.969487 | -0.76228 |
| H | 3.620488 | -0.67908 | 1.226768 | H | 3.624584 | -0.68486 | 1.227721 |
| H | 0.385618 | -1.19535 | -1.47317 | H | 0.394585 | -1.19552 | -1.47545 |
| H | 5.722413 | 1.721332 | -1.19389 | H | 5.730911 | 1.712832 | -1.19231 |
| H | 5.767525 | 2.714116 | 0.256186 | H | 5.779193 | 2.706279 | 0.257235 |
| H | 5.614317 | 0.712878 | 1.681767 | H | 5.620638 | 0.705343 | 1.683653 |
| H | 3.463485 | 2.595062 | -0.69866 | H | 3.474078 | 2.591318 | -0.6958 |
| H | 3.526446 | 1.957899 | 0.936298 | H | 3.537154 | 1.953789 | 0.939319 |
| H | 3.544724 | -2.2563 | -1.38884 | H | 3.54664 | -2.25744 | -1.39049 |
| H | 3.724177 | -2.94021 | 0.203446 | H | 3.727412 | -2.9448 | 0.200355 |
| H | 1.374194 | -3.03878 | -0.66675 | H | 1.376224 | -3.03996 | -0.66412 |
| H | 1.587366 | -2.35809 | 0.918704 | H | 1.593209 | -2.35791 | 0.920254 |
| H | 0.04031 | 2.210549 | 0.792202 | H | 0.082027 | 2.185954 | 0.81226 |
| H | 1.552741 | 2.531743 | -1.07349 | H | 1.567221 | 2.532373 | -1.06551 |
| H | 0.531057 | 1.624897 | -2.14533 | H | 0.524741 | 1.647477 | -2.13088 |
| H | -2.68187 | -1.11239 | 2.387275 | H | -2.67537 | -1.10823 | 2.371074 |
| H | -0.69123 | -2.2527 | 1.761134 | H | -0.7114 | -2.27933 | 1.716594 |
| H | -1.30986 | -2.33812 | 0.118296 | H | -1.32553 | -2.31735 | 0.069735 |
| H | -3.29265 | 2.140149 | 0.05608 | H | -3.30523 | 2.097748 | -0.0333 |
| H | -7.58064 | -0.41698 | 0.391479 | H | -7.5996 | -0.41174 | 0.409628 |
| H | -6.49178 | 1.622354 | 0.770848 | H | -6.49976 | 1.625546 | 0.761736 |
| H | -5.91687 | 1.701437 | -0.88413 | H | -5.93718 | 1.685608 | -0.89918 |
| H | 3.038677 | -0.68096 | -2.51192 | H | 3.040897 | -0.68055 | -2.51165 |
| H | 2.961819 | 1.123541 | -2.66389 | H | 2.966249 | 1.124105 | -2.66105 |
| H | 5.851519 | -2.08025 | -1.59249 | H | 5.855604 | -2.08831 | -1.58986 |
| H | 7.103153 | -0.86491 | -1.29356 | H | 7.107835 | -0.87321 | -1.2922 |
| H | 5.576958 | -0.39743 | -2.04947 | H | 5.581156 | -0.4058 | -2.04766 |
| H | 5.726215 | -2.85787 | 0.778858 | H | 5.725722 | -2.86518 | 0.78183 |
| H | 5.653264 | -1.69044 | 2.106951 | H | 5.656982 | -1.69672 | 2.109198 |
| H | 7.10673 | -1.83171 | 1.097817 | H | 7.109447 | -1.84298 | 1.099244 |
| H | -2.20364 | -0.79631 | -1.36509 | H | -2.42726 | 0.990054 | -1.70812 |
| H | -1.01744 | 0.179539 | -2.22284 | H | -0.97734 | 0.16092 | -2.21961 |
| H | -2.52038 | 0.902186 | -1.66312 | H | -2.2408 | -0.73676 | -1.38285 |
| H | -4.7576 | 2.810554 | 1.96831 | H | -4.74172 | 2.874951 | 1.861242 |
| H | -3.03662 | 2.907929 | 2.357946 | H | -3.02338 | 2.955804 | 2.269307 |
| H | -4.01247 | 1.557116 | 2.971397 | H | -4.02406 | 1.64454 | 2.911641 |
| H | 7.761359 | 0.158896 | 1.244556 | H | 7.76715 | 0.142868 | 1.24377 |
| H | -6.73071 | -0.03884 | -2.53252 | H | -7.92569 | -1.25966 | -1.91954 |
| H | -7.8981 | -1.23474 | -1.94977 | H | -8.27659 | 0.479509 | -1.80549 |
| H | -8.25721 | 0.501158 | -1.81221 | H | -6.75371 | -0.07721 | -2.521 |
| H | -1.26481 | 2.819473 | -1.6052 | H | -1.45653 | 3.433725 | -0.04946 |
| C | 0.643968 | -0.05455 | 1.790489 | C | 0.633211 | -0.07781 | 1.798011 |
| H | 1.348214 | 0.756225 | 1.599299 | H | 1.360294 | 0.715336 | 1.617731 |
| H | -0.03079 | 0.270954 | 2.58505 | H | -0.04598 | 0.264304 | 2.582344 |
| H | 1.220152 | -0.88598 | 2.204481 | H | 1.182454 | -0.92242 | 2.221768 |
| **1g** | | | | **1h** | | | |
| C | 5.369225 | 1.783144 | -0.12097 | C | 5.364238 | 1.254865 | -1.37332 |
| C | 5.975736 | 0.602207 | 0.633838 | C | 6.164128 | 0.257231 | -0.53178 |
| C | 5.468142 | -0.77775 | 0.114242 | C | 5.366983 | -1.02549 | -0.16883 |
| C | 3.89584 | -0.77787 | 0.16796 | C | 4.032163 | -0.55878 | 0.510923 |
| C | 3.256234 | 0.435166 | -0.54157 | C | 3.217393 | 0.411649 | -0.3625 |
| C | 3.838173 | 1.760969 | -0.06403 | C | 4.044497 | 1.651526 | -0.69672 |
| C | 3.282769 | -2.10103 | -0.31652 | C | 3.1766 | -1.69191 | 1.096473 |
| C | 1.765822 | -2.15642 | -0.10375 | C | 1.947301 | -1.1111 | 1.801057 |
| C | 0.944194 | -0.91059 | -0.55335 | C | 0.981913 | -0.46198 | 0.797221 |
| C | 1.771365 | 0.348265 | -0.93812 | C | 1.694988 | 0.539694 | -0.17099 |
| C | -0.113 | -0.51745 | 0.526026 | C | -0.28364 | 0.162489 | 1.437524 |
| C | -1.10039 | 0.576832 | -0.03808 | C | -1.14435 | 0.846352 | 0.309097 |
| C | -0.2951 | 1.87746 | -0.19671 | C | -0.44346 | 2.152832 | -0.10828 |
| C | 0.924294 | 1.635915 | -1.11303 | C | 1.097627 | 1.959715 | -0.30559 |
| C | -2.20808 | 0.57615 | 1.054666 | C | -2.51206 | 1.024463 | 1.029056 |
| C | -2.42166 | -0.94023 | 1.343446 | C | -2.70851 | -0.32618 | 1.770607 |
| O | -3.46833 | -1.56985 | 0.562099 | O | -3.40838 | -1.33949 | 1.003989 |
| C | -1.09434 | -1.643 | 0.95886 | C | -1.28413 | -0.8701 | 2.04134 |
| C | -3.50316 | 1.410767 | 0.83291 | C | -3.7406 | 1.529239 | 0.21997 |
| C | -4.64324 | 0.540108 | 0.398997 | C | -4.61548 | 0.384411 | -0.19342 |
| C | -4.54979 | -0.80168 | 0.304134 | C | -4.4004 | -0.88557 | 0.206193 |
| C | -5.79511 | -1.402 | -0.21826 | C | -5.36519 | -1.82238 | -0.40685 |
| C | -6.80421 | -0.25235 | -0.38031 | C | -6.34809 | -0.96919 | -1.22728 |
| C | -6.00573 | 1.030022 | -0.0339 | C | -5.79794 | 0.47638 | -1.12989 |
| C | 2.861061 | 0.262785 | -1.99207 | C | 2.271684 | -0.09303 | -1.42799 |
| C | 6.011601 | -1.04836 | -1.30503 | C | 5.136625 | -1.89433 | -1.42207 |
| C | 6.031122 | -1.85584 | 1.064402 | C | 6.21956 | -1.83218 | 0.834277 |
| C | -1.72998 | 0.17788 | -1.39738 | C | -1.31482 | -0.06093 | -0.93603 |
| C | -3.86095 | 2.231679 | 2.09185 | C | -4.53549 | 2.59237 | 1.010972 |
| O | 7.401521 | 0.689046 | 0.667831 | O | 7.361989 | -0.13826 | -1.20383 |
| C | -7.45987 | -0.2661 | -1.76423 | C | -6.51944 | -1.51333 | -2.6486 |
| H | -1.75046 | 0.979907 | 1.961664 | H | -2.3503 | 1.762545 | 1.821084 |
| O | -5.99658 | -2.57212 | -0.48608 | O | -5.38521 | -3.03513 | -0.3052 |
| O | -1.05791 | 2.984695 | -0.69078 | O | -1.04517 | 2.777301 | -1.24927 |
| H | 3.642577 | -0.67923 | 1.23677 | H | 4.351623 | 0.039628 | 1.381775 |
| H | 0.391874 | -1.20755 | -1.45165 | H | 0.618291 | -1.28935 | 0.175889 |
| H | 5.700172 | 1.7614 | -1.16883 | H | 5.16584 | 0.814766 | -2.35725 |
| H | 5.754615 | 2.713234 | 0.311386 | H | 5.976833 | 2.150569 | -1.54846 |
| H | 5.67771 | 0.679427 | 1.688656 | H | 6.4248 | 0.749616 | 0.422775 |
| H | 3.44967 | 2.599918 | -0.64663 | H | 3.49773 | 2.338178 | -1.34771 |
| H | 3.522053 | 1.93269 | 0.975891 | H | 4.260515 | 2.205431 | 0.228797 |
| H | 3.527568 | -2.26915 | -1.36995 | H | 2.862021 | -2.39775 | 0.316727 |
| H | 3.731117 | -2.938 | 0.227035 | H | 3.771414 | -2.26804 | 1.811224 |
| H | 1.367325 | -3.04388 | -0.60726 | H | 1.418643 | -1.89861 | 2.352309 |
| H | 1.599774 | -2.33343 | 0.962631 | H | 2.290225 | -0.38123 | 2.544467 |
| H | 0.031525 | 2.218981 | 0.788821 | H | -0.59476 | 2.893213 | 0.682639 |
| H | 1.553243 | 2.527508 | -1.07218 | H | 1.610351 | 2.63381 | 0.390749 |
| H | 0.536327 | 1.613363 | -2.14169 | H | 1.32677 | 2.354992 | -1.29918 |
| H | -2.69331 | -1.0964 | 2.394399 | H | -3.2918 | -0.18387 | 2.688366 |
| H | -0.69739 | -2.23759 | 1.786252 | H | -1.11002 | -1.02938 | 3.109271 |
| H | -1.30908 | -2.33687 | 0.141551 | H | -1.20294 | -1.84712 | 1.556572 |
| H | -3.29665 | 2.139516 | 0.038471 | H | -3.36135 | 2.0281 | -0.68194 |
| H | -7.58347 | -0.41952 | 0.375251 | H | -7.31516 | -1.03389 | -0.71106 |
| H | -6.49813 | 1.623122 | 0.747141 | H | -6.55602 | 1.182524 | -0.76775 |
| H | -5.91766 | 1.693574 | -0.90629 | H | -5.48171 | 0.851355 | -2.11396 |
| H | 3.051251 | -0.67893 | -2.495 | H | 2.141059 | -1.16389 | -1.55841 |
| H | 2.959179 | 1.125118 | -2.64882 | H | 2.223443 | 0.467681 | -2.36009 |
| H | 5.741794 | -2.05159 | -1.64621 | H | 4.615004 | -2.82098 | -1.16267 |
| H | 7.106267 | -1.00509 | -1.30607 | H | 6.097372 | -2.16321 | -1.86711 |
| H | 5.638694 | -0.33742 | -2.04702 | H | 4.545627 | -1.38349 | -2.18503 |
| H | 5.85976 | -2.86867 | 0.689039 | H | 5.786138 | -2.81564 | 1.037279 |
| H | 5.577063 | -1.78298 | 2.059771 | H | 6.317619 | -1.30272 | 1.789969 |
| H | 7.108881 | -1.71568 | 1.178828 | H | 7.22262 | -1.98407 | 0.428885 |
| H | -2.51652 | 0.888912 | -1.66919 | H | -2.08757 | 0.34447 | -1.59508 |
| H | -1.00959 | 0.165139 | -2.21714 | H | -0.39216 | -0.14202 | -1.51585 |
| H | -2.19775 | -0.80646 | -1.35724 | H | -1.63095 | -1.07011 | -0.66939 |
| H | -4.76859 | 2.823431 | 1.940032 | H | -5.40276 | 2.946043 | 0.445464 |
| H | -3.0491 | 2.924361 | 2.335254 | H | -3.90269 | 3.459319 | 1.22671 |
| H | -4.02672 | 1.577908 | 2.955344 | H | -4.89793 | 2.186743 | 1.962065 |
| H | 7.706723 | 0.813847 | -0.24055 | H | 7.871634 | 0.661068 | -1.38828 |
| H | -8.25273 | 0.485076 | -1.83624 | H | -6.79376 | -2.57105 | -2.61244 |
| H | -6.72277 | -0.05704 | -2.54758 | H | -7.29682 | -0.96715 | -3.19211 |
| H | -7.89094 | -1.2511 | -1.96257 | H | -5.58547 | -1.42664 | -3.21494 |
| H | -1.26832 | 2.805022 | -1.61748 | H | -0.93374 | 2.179856 | -2.00097 |
| C | 0.63526 | -0.03739 | 1.803868 | C | 0.118929 | 1.144015 | 2.572571 |
| H | 1.337644 | 0.774204 | 1.609417 | H | 0.532759 | 0.578928 | 3.41163 |
| H | -0.04334 | 0.292237 | 2.593381 | H | 0.879154 | 1.866612 | 2.268476 |
| **1i** | | | | **1j** | | | |
| C | 5.38155 | 1.774754 | -0.12505 | C | 5.36923 | 1.275192 | -1.36018 |
| C | 5.987885 | 0.593214 | 0.628681 | C | 6.167704 | 0.279022 | -0.52484 |
| C | 5.474874 | -0.78578 | 0.112223 | C | 5.369944 | -1.02003 | -0.18423 |
| C | 3.90265 | -0.78195 | 0.169969 | C | 4.032827 | -0.57037 | 0.502722 |
| C | 3.263033 | 0.432445 | -0.53721 | C | 3.218578 | 0.406496 | -0.36389 |
| C | 3.850764 | 1.756456 | -0.06162 | C | 4.042347 | 1.651164 | -0.6878 |
| C | 3.285594 | -2.10372 | -0.3129 | C | 3.176189 | -1.71256 | 1.070526 |
| C | 1.769972 | -2.15475 | -0.09243 | C | 1.943057 | -1.14273 | 1.77783 |
| C | 0.949437 | -0.9099 | -0.54641 | C | 0.978945 | -0.47945 | 0.781981 |
| C | 1.775837 | 0.350494 | -0.9299 | C | 1.694542 | 0.530449 | -0.17405 |
| C | -0.11526 | -0.52008 | 0.526499 | C | -0.28388 | 0.140039 | 1.431539 |
| C | -1.09026 | 0.584062 | -0.04138 | C | -1.1447 | 0.842844 | 0.312828 |
| C | -0.27187 | 1.879873 | -0.17761 | C | -0.43923 | 2.149212 | -0.06852 |
| C | 0.934811 | 1.642054 | -1.1013 | C | 1.095071 | 1.951651 | -0.30627 |
| C | -2.21471 | 0.578523 | 1.035608 | C | -2.51224 | 1.012682 | 1.037181 |
| C | -2.42964 | -0.93966 | 1.3256 | C | -2.70883 | -0.34595 | 1.765505 |
| O | -3.48342 | -1.57289 | 0.559023 | O | -3.42042 | -1.35057 | 0.999677 |
| C | -1.10667 | -1.64443 | 0.932965 | C | -1.28533 | -0.89961 | 2.018942 |
| C | -3.50865 | 1.410949 | 0.784762 | C | -3.74109 | 1.524553 | 0.232707 |
| C | -4.65694 | 0.53501 | 0.383127 | C | -4.61838 | 0.384975 | -0.18908 |
| C | -4.56497 | -0.80726 | 0.303494 | C | -4.40851 | -0.88802 | 0.202573 |
| C | -5.81819 | -1.41234 | -0.19707 | C | -5.37489 | -1.81741 | -0.41844 |
| C | -6.82408 | -0.26226 | -0.37288 | C | -6.34938 | -0.95631 | -1.24078 |
| C | -6.02156 | 1.02281 | -0.04554 | C | -5.79475 | 0.486621 | -1.13187 |
| C | 2.862359 | 0.262834 | -1.98661 | C | 2.274248 | -0.09219 | -1.43283 |
| C | 6.013305 | -1.05925 | -1.30842 | C | 5.148348 | -1.86549 | -1.45295 |
| C | 6.037949 | -1.86424 | 1.061941 | C | 6.214396 | -1.84698 | 0.808866 |
| C | -1.70636 | 0.208043 | -1.41169 | C | -1.31609 | -0.03504 | -0.95162 |
| C | -3.86217 | 2.284769 | 2.008859 | C | -4.53493 | 2.581667 | 1.032896 |
| O | 7.414189 | 0.676551 | 0.657759 | O | 7.373618 | 0.023322 | -1.2464 |
| C | -7.4791 | -0.29254 | -1.75715 | C | -6.51148 | -1.49177 | -2.66653 |
| H | -1.77605 | 0.981371 | 1.953632 | H | -2.35221 | 1.741387 | 1.838826 |
| O | -6.02555 | -2.5864 | -0.43992 | O | -5.40334 | -3.0304 | -0.32001 |
| O | -1.00307 | 2.966728 | -0.77228 | O | -1.08502 | 2.697809 | -1.22628 |
| H | 3.652346 | -0.68361 | 1.239722 | H | 4.348191 | 0.018484 | 1.382012 |
| H | 0.401797 | -1.20837 | -1.4464 | H | 0.611923 | -1.29688 | 0.150075 |
| H | 5.707707 | 1.75071 | -1.17434 | H | 5.181065 | 0.843239 | -2.34979 |
| H | 5.771599 | 2.704382 | 0.304316 | H | 5.986644 | 2.165743 | -1.52045 |
| H | 5.693857 | 0.672236 | 1.68452 | H | 6.412094 | 0.76481 | 0.43717 |
| H | 3.461935 | 2.596264 | -0.64248 | H | 3.492908 | 2.336281 | -1.33848 |
| H | 3.539934 | 1.92883 | 0.980044 | H | 4.248554 | 2.202829 | 0.241642 |
| H | 3.524772 | -2.27142 | -1.36765 | H | 2.867328 | -2.40907 | 0.280135 |
| H | 3.734666 | -2.94209 | 0.228015 | H | 3.765603 | -2.29739 | 1.783033 |
| H | 1.36674 | -3.04427 | -0.58864 | H | 1.414006 | -1.93879 | 2.316284 |
| H | 1.609969 | -2.32546 | 0.975948 | H | 2.284361 | -0.42403 | 2.532878 |
| H | 0.069963 | 2.195748 | 0.815633 | H | -0.56392 | 2.859542 | 0.761071 |
| H | 1.568366 | 2.529839 | -1.05418 | H | 1.631269 | 2.632106 | 0.366979 |
| H | 0.531563 | 1.640069 | -2.12042 | H | 1.291042 | 2.314978 | -1.31924 |
| H | -2.69193 | -1.09147 | 2.379668 | H | -3.28245 | -0.20833 | 2.690193 |
| H | -0.72 | -2.26207 | 1.748307 | H | -1.10669 | -1.08233 | 3.08244 |
| H | -1.32417 | -2.31602 | 0.09815 | H | -1.21077 | -1.86564 | 1.511953 |
| H | -3.31097 | 2.096311 | -0.05105 | H | -3.36244 | 2.025542 | -0.66762 |
| H | -7.60391 | -0.41697 | 0.384602 | H | -7.32055 | -1.02081 | -0.7322 |
| H | -6.50939 | 1.623963 | 0.732429 | H | -6.55309 | 1.193351 | -0.77098 |
| H | -5.93806 | 1.676428 | -0.92578 | H | -5.46969 | 0.865446 | -2.11149 |
| H | 3.048071 | -0.67887 | -2.49133 | H | 2.141292 | -1.16195 | -1.56962 |
| H | 2.959663 | 1.125444 | -2.6429 | H | 2.221987 | 0.473571 | -2.36113 |
| H | 5.740597 | -2.06245 | -1.64733 | H | 4.674528 | -2.82228 | -1.21095 |
| H | 7.108078 | -1.01792 | -1.31339 | H | 6.11045 | -2.0737 | -1.92825 |
| H | 5.638797 | -0.34873 | -2.04998 | H | 4.519654 | -1.35934 | -2.18736 |
| H | 5.86242 | -2.87708 | 0.688537 | H | 5.747081 | -2.80759 | 1.040305 |
| H | 5.587226 | -1.78878 | 2.058666 | H | 6.36861 | -1.31056 | 1.75309 |
| H | 7.116495 | -1.72706 | 1.172774 | H | 7.198735 | -2.08001 | 0.386296 |
| H | -2.23198 | -0.74717 | -1.37387 | H | -2.05019 | 0.4192 | -1.61842 |
| H | -0.96567 | 0.147954 | -2.20916 | H | -0.38806 | -0.11839 | -1.51815 |
| H | -2.4213 | 0.976657 | -1.71291 | H | -1.66258 | -1.03958 | -0.70407 |
| H | -3.04181 | 2.971967 | 2.246464 | H | -5.40285 | 2.940392 | 0.471371 |
| H | -4.04529 | 1.665159 | 2.893302 | H | -3.90219 | 3.447057 | 1.256744 |
| H | -4.75777 | 2.886915 | 1.829378 | H | -4.89691 | 2.169206 | 1.981253 |
| H | 7.716281 | 0.797513 | -0.25217 | H | 7.984504 | -0.43712 | -0.65791 |
| H | -6.74067 | -0.09719 | -2.54261 | H | -7.28294 | -0.93986 | -3.2128 |
| H | -7.91427 | -1.27838 | -1.94181 | H | -5.57285 | -1.4046 | -3.22488 |
| H | -8.26893 | 0.460731 | -1.83958 | H | -6.78957 | -2.5488 | -2.63866 |
| H | -1.46699 | 3.432852 | -0.06675 | H | -0.71227 | 3.575412 | -1.38087 |
| C | 0.621185 | -0.05768 | 1.818175 | C | 0.12472 | 1.101999 | 2.581139 |
| H | 1.348668 | 0.734288 | 1.634342 | H | 0.894072 | 1.82029 | 2.288084 |
| H | -0.06276 | 0.292166 | 2.594867 | H | -0.72136 | 1.666579 | 2.980943 |
| H | 1.168601 | -0.89697 | 2.254442 | H | 0.530244 | 0.522448 | 3.414424 |
| **1k** | | | | **1l** | | | |
| C | 5.625929 | 1.370375 | -0.70895 | C | 5.474621 | 1.466828 | -1.02819 |
| C | 5.945518 | -0.05675 | -1.14147 | C | 6.247732 | 0.336053 | -0.3456 |
| C | 5.38892 | -1.09971 | -0.10658 | C | 5.455255 | -0.99761 | -0.26754 |
| C | 4.094085 | -0.53492 | 0.591533 | C | 4.080447 | -0.67912 | 0.419548 |
| C | 3.289806 | 0.388471 | -0.33218 | C | 3.297217 | 0.438888 | -0.29064 |
| C | 4.103702 | 1.635472 | -0.71207 | C | 4.12208 | 1.722029 | -0.34967 |
| C | 3.226646 | -1.59165 | 1.294291 | C | 3.209638 | -1.90839 | 0.721294 |
| C | 2.009951 | -0.93137 | 1.949083 | C | 1.943948 | -1.48298 | 1.470736 |
| C | 1.045254 | -0.38689 | 0.882394 | C | 1.018613 | -0.63129 | 0.586445 |
| C | 1.763887 | 0.525619 | -0.1622 | C | 1.766862 | 0.53005 | -0.14444 |
| C | -0.23011 | 0.28664 | 1.451027 | C | -0.25556 | -0.12647 | 1.309516 |
| C | -1.07339 | 0.87515 | 0.255641 | C | -1.08437 | 0.782227 | 0.32043 |
| C | -0.36387 | 2.139314 | -0.24732 | C | -0.35126 | 2.131649 | 0.215074 |
| C | 1.178853 | 1.93359 | -0.40445 | C | 1.171478 | 1.956661 | -0.05739 |
| C | -1.2394 | -0.6959 | 2.116703 | C | -2.47089 | 0.823314 | 1.031546 |
| C | -2.65866 | -0.16642 | 1.79693 | C | -2.68584 | -0.64129 | 1.509102 |
| C | -2.45184 | 1.112767 | 0.93794 | O | -3.39777 | -1.48984 | 0.574558 |
| O | -3.36845 | -1.24115 | 1.131617 | C | -1.27272 | -1.24562 | 1.673983 |
| C | -4.3504 | -0.85785 | 0.28678 | C | -3.68571 | 1.472839 | 0.301291 |
| C | -4.54996 | 0.368443 | -0.23745 | C | -4.60908 | 0.430322 | -0.25544 |
| C | -3.66617 | 1.540884 | 0.065144 | C | -4.40393 | -0.89229 | -0.09611 |
| C | -5.32173 | -1.84104 | -0.23526 | C | -5.43824 | -1.69292 | -0.78785 |
| C | -6.28434 | -1.06666 | -1.15198 | C | -6.33061 | -0.6954 | -1.54471 |
| C | -5.72192 | 0.376902 | -1.19141 | C | -5.84821 | 0.701722 | -1.07705 |
| O | 5.405987 | -0.18885 | -2.45905 | C | 2.402356 | 0.139165 | -1.47008 |
| C | 6.47356 | -1.31616 | 0.971017 | C | 5.300176 | -1.61853 | -1.67073 |
| C | 5.149918 | -2.44562 | -0.81823 | C | 6.272772 | -1.97054 | 0.609582 |
| C | 2.356119 | -0.19201 | -1.36244 | C | -1.2313 | 0.1643 | -1.08952 |
| O | -0.97577 | 2.545552 | -1.47967 | C | -4.43338 | 2.466045 | 1.217236 |
| C | -1.22745 | -0.12653 | -0.91522 | O | 7.484885 | 0.080041 | -1.01414 |
| C | -4.45807 | 2.689821 | 0.728359 | H | -2.33074 | 1.398108 | 1.953507 |
| C | -6.43591 | -1.74983 | -2.51449 | O | -5.56859 | -2.90239 | -0.77286 |
| O | -5.36132 | -3.03605 | -0.00621 | O | -0.85942 | 2.983994 | -0.82603 |
| H | 6.043695 | 1.534087 | 0.290598 | H | 4.346281 | -0.26232 | 1.406751 |
| H | 6.129648 | 2.075586 | -1.37765 | H | 0.661789 | -1.31144 | -0.19651 |
| H | 7.039347 | -0.18901 | -1.18665 | H | 5.326168 | 1.214441 | -2.08433 |
| H | 3.804658 | 1.984431 | -1.70368 | H | 6.082195 | 2.382696 | -1.0069 |
| H | 3.894335 | 2.456079 | -0.01317 | H | 6.451181 | 0.644972 | 0.695976 |
| H | 3.823502 | -2.1159 | 2.049734 | H | 3.592876 | 2.516321 | -0.88198 |
| H | 2.88242 | -2.35774 | 0.588831 | H | 4.293363 | 2.092872 | 0.672143 |
| H | 2.357978 | -0.12839 | 2.60993 | H | 2.938786 | -2.43991 | -0.20004 |
| H | 1.481367 | -1.65536 | 2.581656 | H | 3.775025 | -2.61831 | 1.33214 |
| H | 0.690219 | -1.26876 | 0.334869 | H | 1.393684 | -2.36596 | 1.818679 |
| H | -0.52204 | 2.937569 | 0.492853 | H | 2.249595 | -0.92967 | 2.366786 |
| H | 1.680526 | 2.650356 | 0.257458 | H | -0.47021 | 2.65874 | 1.171172 |
| H | 1.43453 | 2.243404 | -1.42248 | H | 1.711841 | 2.531158 | 0.703927 |
| H | -1.1592 | -1.70334 | 1.699059 | H | 1.353868 | 2.474883 | -1.00195 |
| H | -1.07402 | -0.7829 | 3.194379 | H | -3.26907 | -0.66027 | 2.438018 |
| H | -3.23847 | 0.065003 | 2.698883 | H | -1.11365 | -1.63067 | 2.6851 |
| H | -3.27194 | 1.932391 | -0.88178 | H | -1.19596 | -2.09691 | 0.991865 |
| H | -7.26055 | -1.07282 | -0.6491 | H | -3.30182 | 2.048943 | -0.55355 |
| H | -6.47809 | 1.121087 | -0.91008 | H | -6.62287 | 1.201995 | -0.47957 |
| H | -5.39119 | 0.649334 | -2.20398 | H | -5.63378 | 1.372786 | -1.91966 |
| H | 5.660525 | -1.05417 | -2.80149 | H | 2.288668 | -0.88975 | -1.80077 |
| H | 6.114242 | -1.97378 | 1.769028 | H | 2.382264 | 0.862275 | -2.28327 |
| H | 7.369166 | -1.77888 | 0.541283 | H | 4.788848 | -2.5846 | -1.61359 |
| H | 6.777587 | -0.37123 | 1.434559 | H | 6.285277 | -1.78623 | -2.11231 |
| H | 4.337379 | -2.38902 | -1.54612 | H | 4.7312 | -0.9818 | -2.35139 |
| H | 4.908334 | -3.23637 | -0.10292 | H | 5.847233 | -2.97809 | 0.604319 |
| H | 6.058962 | -2.76863 | -1.34343 | H | 6.315184 | -1.62757 | 1.650675 |
| H | 2.228287 | -1.27056 | -1.41103 | H | 7.296763 | -2.03614 | 0.234419 |
| H | 2.32207 | 0.293826 | -2.33437 | H | -1.91855 | 0.766296 | -1.68692 |
| H | -0.59147 | 3.395619 | -1.72989 | H | -0.28333 | 0.152015 | -1.6287 |
| H | -1.95231 | 0.256827 | -1.63455 | H | -1.62171 | -0.85442 | -1.05057 |
| H | -0.29176 | -0.26588 | -1.45705 | H | -5.2741 | 2.937839 | 0.699621 |
| H | -1.57868 | -1.10043 | -0.57049 | H | -3.76235 | 3.262634 | 1.558098 |
| H | -3.81667 | 3.566795 | 0.86488 | H | -4.82727 | 1.957834 | 2.103905 |
| H | -5.31174 | 2.995126 | 0.115529 | H | 7.983831 | 0.90672 | -1.03266 |
| H | -4.84082 | 2.39042 | 1.710355 | H | -1.64992 | 3.423961 | -0.48993 |
| H | -7.20101 | -1.25759 | -3.12317 | C | 0.133651 | 0.606409 | 2.624406 |
| H | -6.71722 | -2.79739 | -2.37676 | H | 0.499919 | -0.121 | 3.353296 |
| H | -5.49208 | -1.72428 | -3.0703 | H | 0.925699 | 1.346383 | 2.485777 |
| C | 0.152225 | 1.350432 | 2.517881 | H | -0.71364 | 1.112672 | 3.094575 |
| H | 0.912533 | 2.056117 | 2.176042 | C | -7.81856 | -0.99115 | -1.34491 |
| H | -0.70721 | 1.932046 | 2.862003 | H | -8.44065 | -0.34815 | -1.97531 |
| H | 0.558409 | 0.848239 | 3.400067 | H | -8.0275 | -2.03473 | -1.59488 |
| H | -2.3031 | 1.917911 | 1.664889 | H | -8.11296 | -0.83037 | -0.30182 |
| H | 4.459292 | 0.11675 | 1.401503 | H | -6.08757 | -0.82218 | -2.60851 |
| **1m** | | | |  | | | |
| C | 5.373993 | 1.276324 | -1.34252 |  |  |  |  |
| C | 6.171237 | 0.262901 | -0.51753 |  |  |  |  |
| C | 5.374777 | -1.02884 | -0.18615 |  |  |  |  |
| C | 4.036387 | -0.5789 | 0.497933 |  |  |  |  |
| C | 3.224984 | 0.409639 | -0.35752 |  |  |  |  |
| C | 4.050815 | 1.657174 | -0.66371 |  |  |  |  |
| C | 3.178578 | -1.72629 | 1.051488 |  |  |  |  |
| C | 1.945068 | -1.16323 | 1.763447 |  |  |  |  |
| C | 0.984001 | -0.48788 | 0.772151 |  |  |  |  |
| C | 1.701042 | 0.533132 | -0.16997 |  |  |  |  |
| C | -0.28073 | 0.123689 | 1.425154 |  |  |  |  |
| C | -1.13379 | 0.849662 | 0.313117 |  |  |  |  |
| C | -0.41417 | 2.167158 | -0.0315 |  |  |  |  |
| C | 1.106929 | 1.956713 | -0.29186 |  |  |  |  |
| C | -2.50855 | 0.999956 | 1.031416 |  |  |  |  |
| C | -2.70481 | -0.36873 | 1.743884 |  |  |  |  |
| O | -3.41619 | -1.36607 | 0.968095 |  |  |  |  |
| C | -1.283 | -0.9253 | 1.990879 |  |  |  |  |
| C | -3.7392 | 1.511342 | 0.224432 |  |  |  |  |
| C | -4.63178 | 0.378175 | -0.18422 |  |  |  |  |
| C | -4.4154 | -0.89774 | 0.19222 |  |  |  |  |
| C | -5.39552 | -1.82078 | -0.42055 |  |  |  |  |
| C | -6.3801 | -0.9515 | -1.22134 |  |  |  |  |
| C | -5.82362 | 0.490658 | -1.10634 |  |  |  |  |
| C | 2.283941 | -0.07545 | -1.43517 |  |  |  |  |
| C | 5.150576 | -1.86969 | -1.45944 |  |  |  |  |
| C | 6.22433 | -1.85637 | 0.80237 |  |  |  |  |
| C | -1.2988 | 0.003219 | -0.97092 |  |  |  |  |
| C | -4.51685 | 2.594854 | 1.00421 |  |  |  |  |
| O | 7.374325 | -0.11585 | -1.19031 |  |  |  |  |
| C | -6.56019 | -1.47271 | -2.65042 |  |  |  |  |
| H | -2.3613 | 1.719009 | 1.844851 |  |  |  |  |
| O | -5.42413 | -3.03364 | -0.3296 |  |  |  |  |
| O | -0.94404 | 2.825603 | -1.19554 |  |  |  |  |
| H | 4.351311 | -0.00149 | 1.384627 |  |  |  |  |
| H | 0.618881 | -1.29853 | 0.130055 |  |  |  |  |
| H | 5.180181 | 0.856332 | -2.33602 |  |  |  |  |
| H | 5.986525 | 2.176106 | -1.49643 |  |  |  |  |
| H | 6.425304 | 0.736032 | 0.448676 |  |  |  |  |
| H | 3.504072 | 2.354748 | -1.303 |  |  |  |  |
| H | 4.262809 | 2.193272 | 0.273422 |  |  |  |  |
| H | 2.86836 | -2.41373 | 0.253949 |  |  |  |  |
| H | 3.769657 | -2.31897 | 1.755863 |  |  |  |  |
| H | 1.414237 | -1.96467 | 2.292321 |  |  |  |  |
| H | 2.285796 | -0.45298 | 2.526669 |  |  |  |  |
| H | -0.52426 | 2.849468 | 0.821816 |  |  |  |  |
| H | 1.65482 | 2.637344 | 0.370454 |  |  |  |  |
| H | 1.278967 | 2.328513 | -1.30453 |  |  |  |  |
| H | -3.28011 | -0.2411 | 2.668961 |  |  |  |  |
| H | -1.10812 | -1.12878 | 3.051197 |  |  |  |  |
| H | -1.20716 | -1.88121 | 1.465261 |  |  |  |  |
| H | -3.36959 | 1.980233 | -0.69878 |  |  |  |  |
| H | -7.34462 | -1.0206 | -0.70114 |  |  |  |  |
| H | -6.57676 | 1.192376 | -0.72544 |  |  |  |  |
| H | -5.5157 | 0.879374 | -2.08775 |  |  |  |  |
| H | 2.154642 | -1.14391 | -1.5853 |  |  |  |  |
| H | 2.231655 | 0.502633 | -2.35576 |  |  |  |  |
| H | 4.629082 | -2.80249 | -1.22275 |  |  |  |  |
| H | 6.113473 | -2.12684 | -1.9068 |  |  |  |  |
| H | 4.561368 | -1.34244 | -2.21241 |  |  |  |  |
| H | 5.789638 | -2.84346 | 0.983927 |  |  |  |  |
| H | 6.319783 | -1.3465 | 1.768951 |  |  |  |  |
| H | 7.228566 | -2.00052 | 0.397014 |  |  |  |  |
| H | -2.00853 | 0.487787 | -1.64387 |  |  |  |  |
| H | -0.3626 | -0.08643 | -1.52259 |  |  |  |  |
| H | -1.67026 | -0.9996 | -0.7535 |  |  |  |  |
| H | -5.37651 | 2.961062 | 0.435177 |  |  |  |  |
| H | -3.87265 | 3.453217 | 1.226063 |  |  |  |  |
| H | -4.88818 | 2.199557 | 1.955706 |  |  |  |  |
| H | 7.876923 | 0.689951 | -1.36581 |  |  |  |  |
| H | -5.62829 | -1.38158 | -3.21922 |  |  |  |  |
| H | -7.33742 | -0.91419 | -3.18144 |  |  |  |  |
| H | -6.83989 | -2.52941 | -2.6296 |  |  |  |  |
| H | -1.7217 | 3.328732 | -0.92486 |  |  |  |  |
| C | 0.124507 | 1.066555 | 2.59195 |  |  |  |  |
| H | 0.52336 | 0.472537 | 3.418122 |  |  |  |  |
| H | 0.898082 | 1.785974 | 2.313245 |  |  |  |  |
| H | -0.72166 | 1.628199 | 2.99657 |  |  |  |  |

**Figure S3.** Calculated and experimental ECDs of **1A** (purple, calculated at the B3LYP-PCM/6-31G(d,p)//B3LYP/6-31G(d,p) level in CH_3_OH; blue, experimental in CH_3_OH).

**1A**

**Figure S4**. Calculated and experimental ECDs of **1B** (purple, calculated at the B3LYP-PCM/6-31G(d,p)//B3LYP/6-31G(d,p) level in CH_3_OH; blue, experimental in CH_3_OH).

**1B**

**Figure S5.** Calculated ECDs spectra of each conformations for **1A** (calculated at the B3LYP-PCM/6-31G(d,p)//B3LYP/6-31G(d,p) level in CH_3_OH).


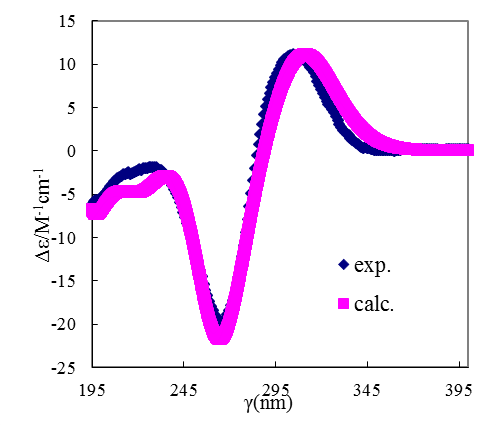

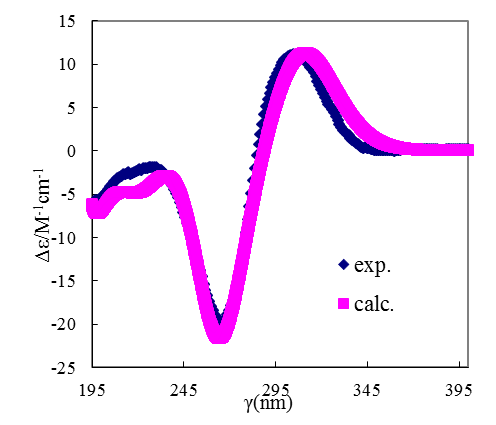

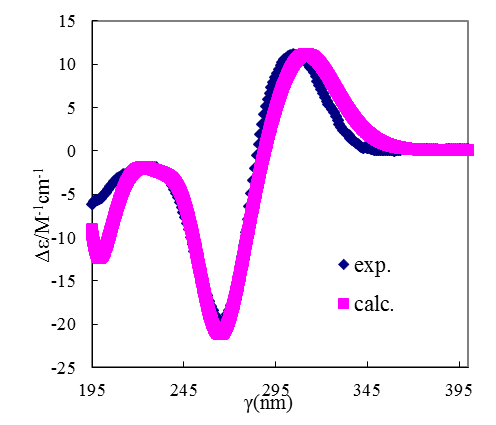


**1a 1b 1c**


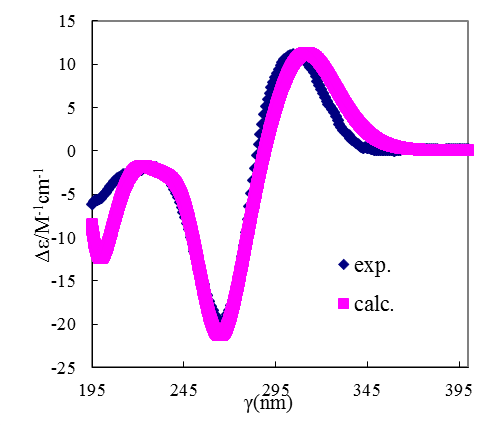

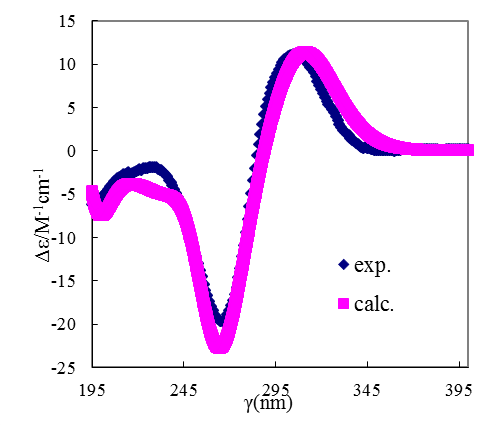

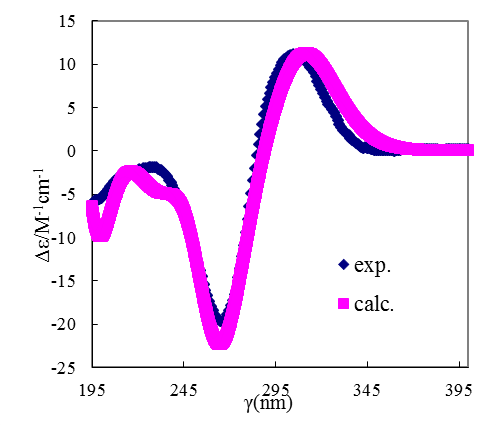


**1d 1e 1f**


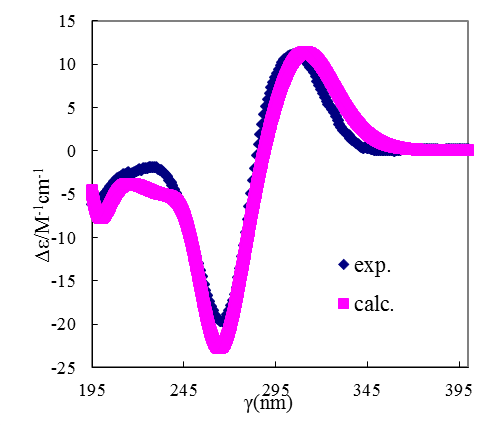

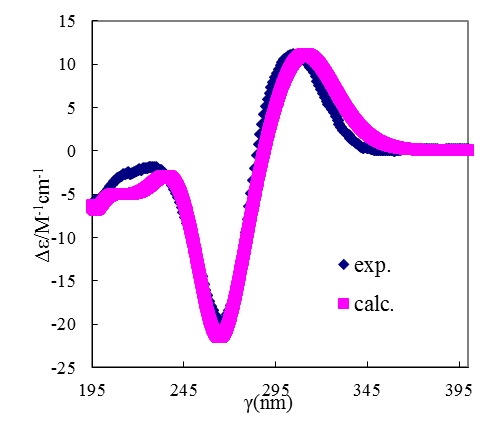

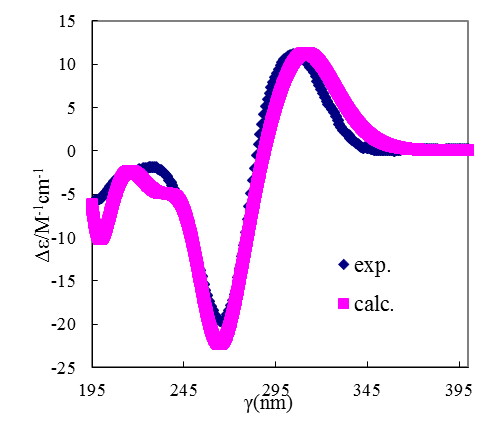


**1g 1h 1i**


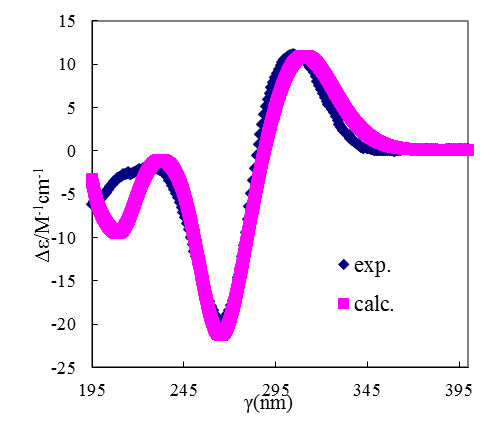

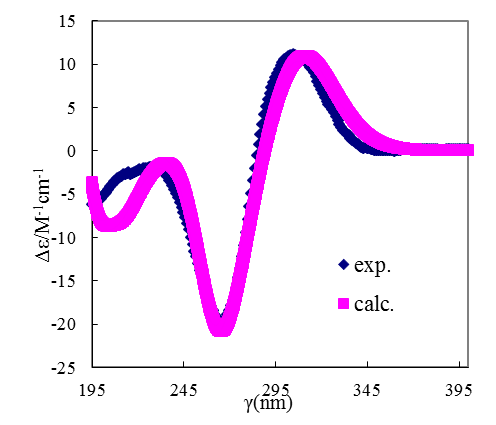

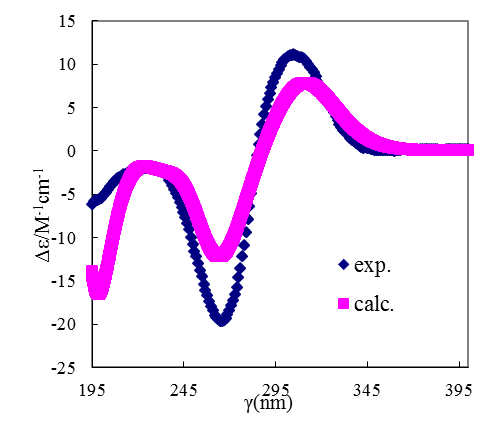


**1j 1k 1l**


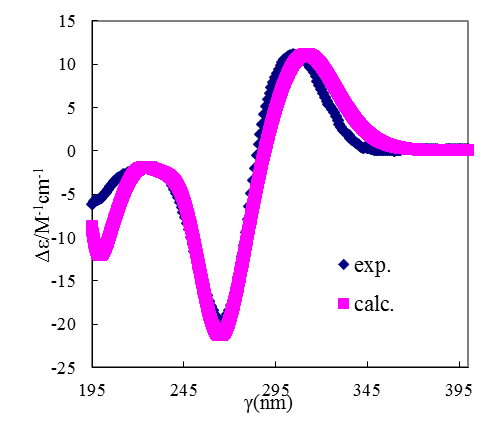


**1m**

**Table S6.** Computed Optical Rotation Considering the Solvation Effect of **1A** and **1B**

|  | Calc. | Exptl. |
| --- | --- | --- |
| **1A** | -52.56 | -52.23 |
| **1B** | 52.56 |

## 5. Computational data of 4

**Figure S6**. Optimized geometries of predominant conformers for compound lactone **A** (**4A**) and lactone **B** (**4B**) at the B3LYP/6-31G(d,p) level in the gas phase.


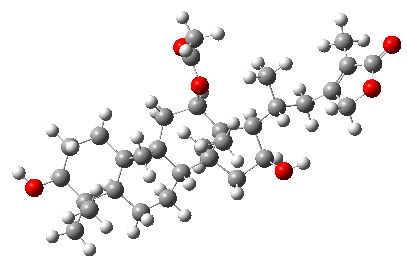

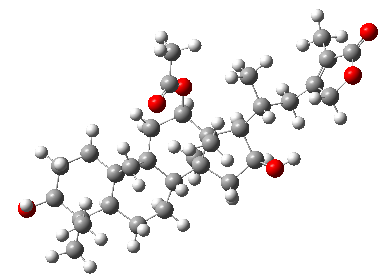


**Lactone A-1 Lactone A-2**


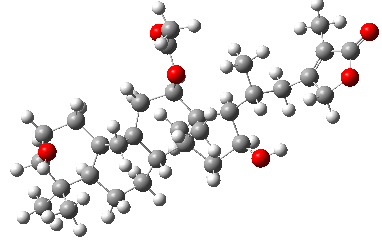


**Lactone A-3**


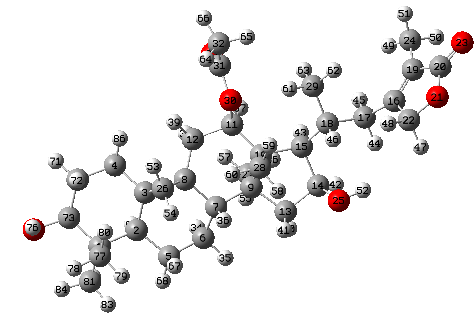


**Lactone A-4**


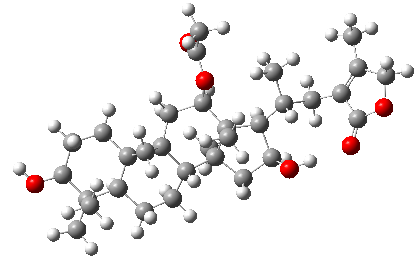

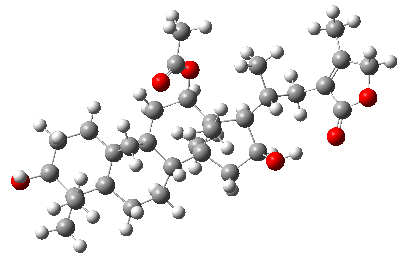


**Lactone B-1 Lactone B-2**


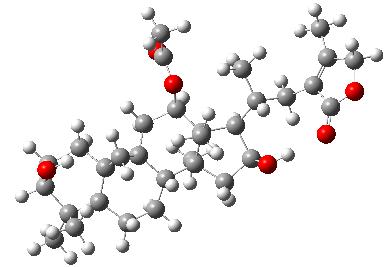


**Lactone B-3**


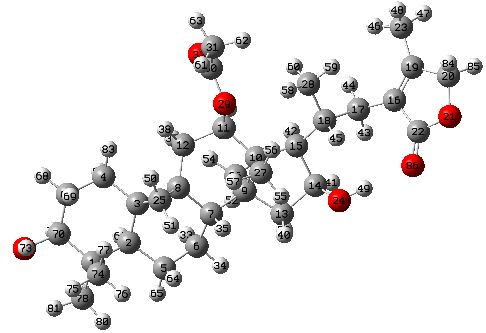


**Lactone B-4**

**Table S7.** Important thermodynamic parameters (a.u.) and Boltzmann distributions of the optimized compound Lactone **A** (**4A**) and lactone **B** (**4B**) at B3LYP/6-31G(d,p) level in the gas phase

| Conformations | E+ZPE | G | % |
| --- | --- | --- | --- |
| **Lactone A-1** | -1698.938187 | -1699.008180 | 44.4 |
| **Lactone A-2** | -1698.928977 | -1698.998453 | 0.0 |
| **Lactone A-3** | -1698.930101 | -1698.999847 | 0.0 |
| **Lactone A-4** | -1698.938488 | -1699.008394 | 55.6 |
| **Lactone B-1** | -1698.947702 | -1699.016392 | 46.3 |
| **Lactone B-2** | -1698.937816 | -1699.004757 | 0.0 |
| **Lactone B-3** | -1698.940397 | -1699.008557 | 0.0 |
| **Lactone B-4** | -1698.948028 | -1699.016532 | 53.7 |

E+ZPE, G: total energy with zero point energy (ZPE) and Gibbs free energy in the gas phase at B3LYP/6-31G(d,p) level, %: Boltzmann distributions, using the relative Gibbs free energies as weighting factors

**Table S8.** Optimized Z-Matrixes of compound Lactone **A** (**4A**) and lactone **B** (**4B**) in the Gas Phase (Å) at B3LYP/6-31G(d,p) level.

| **Lactone A-1** | | | | **Lactone A-2** | | | |
| --- | --- | --- | --- | --- | --- | --- | --- |
| C | 6.231901 | -0.65411 | -0.35942 | C | 6.21202 | -0.6194 | -0.29842 |
| C | 4.813061 | -0.832 | 0.282901 | C | 4.764593 | -0.90827 | 0.231443 |
| C | 3.872935 | 0.354006 | 0.003205 | C | 3.852687 | 0.32984 | 0.197695 |
| C | 4.477156 | 1.645437 | 0.552499 | C | 4.451195 | 1.451115 | 1.044921 |
| C | 4.153655 | -2.19561 | 0.028626 | C | 4.095069 | -2.15978 | -0.35704 |
| C | 2.804626 | -2.27479 | 0.750653 | C | 2.72566 | -2.37959 | 0.292441 |
| C | 1.794901 | -1.30107 | 0.124335 | C | 1.741447 | -1.26643 | -0.10251 |
| C | 2.348561 | 0.148767 | 0.014174 | C | 2.323859 | 0.158831 | 0.117809 |
| C | 0.381707 | -1.33788 | 0.759571 | C | 0.328331 | -1.42173 | 0.513929 |
| C | -0.55462 | -0.26112 | 0.057754 | C | -0.60564 | -0.20855 | 0.069286 |
| C | -0.05764 | 1.124291 | 0.540394 | C | -0.08004 | 1.019576 | 0.861059 |
| C | 1.493459 | 1.246281 | 0.658765 | C | 1.475401 | 1.114568 | 0.969721 |
| C | -0.36987 | -2.65429 | 0.480819 | C | -0.43995 | -2.63742 | -0.0387 |
| C | -1.8713 | -2.31114 | 0.495657 | C | -1.93613 | -2.30148 | 0.088849 |
| C | -1.97775 | -0.73671 | 0.544395 | C | -2.02955 | -0.76844 | 0.463382 |
| C | -5.83895 | -0.57885 | 0.00731 | C | -5.90284 | -0.51007 | 0.062888 |
| C | -4.51051 | -0.7912 | 0.670696 | C | -4.55584 | -0.85335 | 0.626233 |
| C | -3.2855 | -0.23434 | -0.12309 | C | -3.34877 | -0.14466 | -0.06954 |
| C | -6.92286 | 0.090033 | 0.438773 | C | -6.97325 | 0.039378 | 0.663499 |
| C | -7.99289 | -0.04098 | -0.58492 | C | -8.07401 | 0.130846 | -0.33133 |
| O | -7.50926 | -0.80148 | -1.62586 | O | -7.62266 | -0.37814 | -1.52816 |
| C | -6.16207 | -1.17759 | -1.3406 | C | -6.26751 | -0.79969 | -1.37356 |
| O | -9.11167 | 0.409926 | -0.57484 | O | -9.19147 | 0.562186 | -0.18855 |
| C | -7.1795 | 0.863869 | 1.690518 | C | -7.19132 | 0.514183 | 2.063052 |
| O | -2.43198 | -2.90278 | -0.68306 | O | -2.53274 | -2.64023 | -1.16886 |
| C | 3.078742 | 0.464537 | -1.27951 | C | 3.102332 | 0.732485 | -1.05082 |
| C | 0.448391 | -1.17642 | 2.304838 | C | 0.407525 | -1.60196 | 2.057495 |
| C | -0.48676 | -0.34586 | -1.4868 | C | -0.55959 | 0.035572 | -1.45498 |
| C | -3.42902 | 1.289425 | -0.22066 | C | -3.50358 | 1.366975 | 0.132334 |
| O | -0.51567 | 2.165267 | -0.37454 | O | -0.68284 | 2.303015 | 0.485699 |
| C | -0.67361 | 3.404836 | 0.147624 | C | -0.21508 | 3.11835 | -0.48471 |
| C | -1.13567 | 4.381128 | -0.9086 | C | -0.99702 | 4.414965 | -0.48787 |
| O | -0.4819 | 3.680597 | 1.312927 | O | 0.697467 | 2.872594 | -1.24389 |
| H | 2.963712 | -2.04887 | 1.812386 | H | 2.862948 | -2.41578 | 1.380143 |
| H | 2.408513 | -3.29629 | 0.699824 | H | 2.316828 | -3.35224 | -0.00715 |
| H | 1.656199 | -1.66171 | -0.9022 | H | 1.600467 | -1.3832 | -1.18401 |
| H | -0.49106 | 1.356438 | 1.515229 | H | -0.46816 | 0.927275 | 1.878352 |
| H | 1.744859 | 1.329051 | 1.721175 | H | 1.729083 | 0.965108 | 2.025343 |
| H | 1.761645 | 2.211944 | 0.228621 | H | 1.7616 | 2.139277 | 0.745399 |
| H | -0.12233 | -3.43904 | 1.201349 | H | -0.18404 | -3.56675 | 0.47791 |
| H | -0.13027 | -3.04682 | -0.51146 | H | -0.22655 | -2.79429 | -1.09969 |
| H | -2.36245 | -2.73931 | 1.37922 | H | -2.40568 | -2.89993 | 0.880508 |
| H | -2.05775 | -0.45847 | 1.603509 | H | -2.09814 | -0.71425 | 1.558106 |
| H | -4.39572 | -1.86935 | 0.848945 | H | -4.44283 | -1.94509 | 0.576362 |
| H | -4.5207 | -0.33019 | 1.665062 | H | -4.53502 | -0.60696 | 1.694084 |
| H | -3.32039 | -0.64919 | -1.13694 | H | -3.40327 | -0.35675 | -1.14331 |
| H | -6.09122 | -2.27439 | -1.33897 | H | -6.19782 | -1.86945 | -1.61598 |
| H | -5.51317 | -0.80142 | -2.14118 | H | -5.64309 | -0.25283 | -2.09089 |
| H | -6.30694 | 0.890488 | 2.346744 | H | -6.29126 | 0.423099 | 2.674933 |
| H | -8.01973 | 0.429677 | 2.242968 | H | -7.99653 | -0.05548 | 2.539603 |
| H | -7.4659 | 1.893035 | 1.449612 | H | -7.51046 | 1.561636 | 2.063882 |
| H | -3.38847 | -2.96862 | -0.57373 | H | -3.48632 | -2.7249 | -1.04875 |
| H | 2.965978 | 1.456618 | -1.71259 | H | 2.987575 | 1.79055 | -1.26527 |
| H | 3.146991 | -0.32792 | -2.02014 | H | 3.186255 | 0.112716 | -1.94013 |
| H | 0.977066 | -2.03186 | 2.733579 | H | 0.882379 | -2.55988 | 2.284377 |
| H | -0.54336 | -1.16622 | 2.763679 | H | -0.57918 | -1.62978 | 2.526692 |
| H | 0.970663 | -0.27908 | 2.638909 | H | 0.991509 | -0.83241 | 2.564581 |
| H | -0.72246 | -1.34916 | -1.84578 | H | -0.83987 | -0.86151 | -2.01004 |
| H | -1.21566 | 0.336273 | -1.92727 | H | -1.27809 | 0.812096 | -1.72852 |
| H | 0.490418 | -0.05321 | -1.87389 | H | 0.415907 | 0.383077 | -1.79088 |
| H | -2.69778 | 1.717188 | -0.90296 | H | -2.8 | 1.930778 | -0.4748 |
| H | -4.42833 | 1.555575 | -0.5796 | H | -4.51577 | 1.683608 | -0.13936 |
| H | -3.29465 | 1.764197 | 0.758947 | H | -3.33526 | 1.652916 | 1.176445 |
| H | -0.5709 | 4.247566 | -1.8342 | H | -2.05949 | 4.221827 | -0.66219 |
| H | -2.19147 | 4.20154 | -1.13681 | H | -0.91343 | 4.90585 | 0.486014 |
| H | -1.02207 | 5.39828 | -0.53444 | H | -0.6076 | 5.066894 | -1.26911 |
| H | 4.00931 | -2.37655 | -1.04472 | H | 3.978876 | -2.07624 | -1.44559 |
| H | 4.811087 | -2.99178 | 0.389706 | H | 4.730549 | -3.0326 | -0.18014 |
| H | 4.988234 | -0.80046 | 1.372314 | H | 4.900441 | -1.13411 | 1.303746 |
| C | 5.87051 | 1.882402 | -0.04799 | C | 5.870508 | 1.79255 | 0.570262 |
| H | 6.321715 | 2.779187 | 0.399616 | H | 6.324246 | 2.550168 | 1.218976 |
| H | 5.788399 | 2.07747 | -1.12336 | H | 5.815519 | 2.231423 | -0.43602 |
| C | 6.804295 | 0.688958 | 0.172945 | C | 6.785117 | 0.565456 | 0.541786 |
| H | 6.944 | 0.564203 | 1.26224 | H | 6.899077 | 0.200633 | 1.571939 |
| O | 8.073653 | 0.90721 | -0.44623 | O | 8.111821 | 0.925172 | 0.151363 |
| H | 8.440712 | 1.723202 | -0.08264 | C | 6.244544 | -0.28117 | -1.80262 |
| C | 6.202087 | -0.65788 | -1.90145 | H | 7.277711 | -0.12732 | -2.13272 |
| H | 7.212903 | -0.518 | -2.29129 | H | 5.844204 | -1.1057 | -2.39991 |
| H | 5.823286 | -1.6127 | -2.27975 | H | 5.668164 | 0.613482 | -2.04995 |
| H | 5.572841 | 0.134881 | -2.3113 | C | 7.124103 | -1.83921 | -0.05447 |
| C | 7.176371 | -1.78037 | 0.112845 | H | 7.029396 | -2.20693 | 0.974429 |
| H | 7.147695 | -1.89334 | 1.203556 | H | 6.892183 | -2.66741 | -0.73065 |
| H | 6.913583 | -2.74435 | -0.33241 | H | 8.167521 | -1.55422 | -0.2121 |
| H | 8.203919 | -1.54575 | -0.17466 | H | 4.489058 | 1.135873 | 2.098336 |
| H | 4.557473 | 1.576211 | 1.647215 | H | 3.836945 | 2.354488 | 1.01201 |
| H | 3.843042 | 2.510647 | 0.343083 | H | 8.047107 | 1.405396 | -0.68498 |
| **Lactone A-3** | | | | **Lactone A-4** | | | |
| C | 6.014979 | 1.622295 | 0.799026 | C | 6.230326 | -0.65508 | -0.35818 |
| C | 6.623893 | 0.900806 | -0.39916 | C | 4.813242 | -0.83273 | 0.285363 |
| C | 6.257472 | -0.62808 | -0.38868 | C | 3.873687 | 0.353711 | 0.007673 |
| C | 4.862517 | -0.84132 | 0.306874 | C | 4.478341 | 1.642987 | 0.561344 |
| C | 3.913528 | 0.344115 | 0.087581 | C | 4.153511 | -2.19739 | 0.036461 |
| C | 4.470627 | 1.621914 | 0.736398 | C | 2.80441 | -2.27389 | 0.758287 |
| C | 4.205072 | -2.20505 | 0.042855 | C | 1.795866 | -1.30136 | 0.128135 |
| C | 2.863957 | -2.30285 | 0.779385 | C | 2.349721 | 0.148479 | 0.017657 |
| C | 1.847519 | -1.33264 | 0.154803 | C | 0.381643 | -1.33801 | 0.761171 |
| C | 2.391973 | 0.120627 | 0.076869 | C | -0.55374 | -0.26103 | 0.058615 |
| C | 0.425303 | -1.38732 | 0.770362 | C | -0.05697 | 1.124014 | 0.542476 |
| C | -0.50043 | -0.2924 | 0.081226 | C | 1.494126 | 1.245971 | 0.661855 |
| C | -0.00841 | 1.087434 | 0.590816 | C | -0.36942 | -2.65441 | 0.480966 |
| C | 1.537263 | 1.192351 | 0.756733 | C | -1.87087 | -2.31125 | 0.493837 |
| C | -0.32607 | -2.69474 | 0.450258 | C | -1.97741 | -0.7369 | 0.543484 |
| C | -1.82709 | -2.34675 | 0.462879 | C | -5.83822 | -0.57907 | 0.003835 |
| C | -1.9287 | -0.77325 | 0.543745 | C | -4.5101 | -0.79193 | 0.667693 |
| C | -5.78463 | -0.57242 | -0.01561 | C | -3.28463 | -0.23424 | -0.12481 |
| C | -4.46208 | -0.81109 | 0.650306 | C | -6.92257 | 0.088819 | 0.435697 |
| C | -3.22724 | -0.24881 | -0.12428 | C | -7.99208 | -0.04125 | -0.58874 |
| C | -6.86048 | 0.108022 | 0.418073 | O | -7.50759 | -0.80022 | -1.63046 |
| C | -7.92745 | 0.004075 | -0.61187 | C | -6.16049 | -1.17628 | -1.34496 |
| O | -7.45027 | -0.7521 | -1.65895 | O | -9.11099 | 0.409214 | -0.57865 |
| C | -6.11023 | -1.15145 | -1.37145 | C | -7.18017 | 0.860837 | 1.68836 |
| O | -9.03961 | 0.471203 | -0.60197 | O | -2.4296 | -2.90204 | -0.6862 |
| C | -7.11134 | 0.872909 | 1.67649 | C | 3.080248 | 0.465415 | -1.2759 |
| O | -2.38275 | -2.91276 | -0.73067 | C | 0.44633 | -1.17711 | 2.306626 |
| O | 6.158521 | 1.608186 | -1.55147 | C | -0.48447 | -0.34508 | -1.48589 |
| C | 3.133546 | 0.487455 | -1.19575 | C | -3.42814 | 1.289579 | -0.22148 |
| C | 0.468572 | -1.26984 | 2.320557 | O | -0.51417 | 2.165932 | -0.37159 |
| C | -0.41636 | -0.35104 | -1.46423 | C | -0.67607 | 3.404182 | 0.15325 |
| C | -3.3558 | 1.278004 | -0.19334 | C | -1.13722 | 4.382187 | -0.90171 |
| O | -0.4281 | 2.136633 | -0.33335 | O | -0.48835 | 3.677162 | 1.319749 |
| C | -0.59215 | 3.375092 | 0.18882 | H | 2.963104 | -2.04452 | 1.819266 |
| C | -0.99799 | 4.361891 | -0.88077 | H | 2.407792 | -3.2953 | 0.710576 |
| O | -0.44554 | 3.643012 | 1.362479 | H | 1.659187 | -1.66337 | -0.89824 |
| C | 6.302262 | -1.17193 | -1.83024 | H | -0.49067 | 1.355292 | 1.517357 |
| C | 7.337697 | -1.36324 | 0.4337 | H | 1.745134 | 1.328374 | 1.724329 |
| H | 6.386781 | 2.651173 | 0.832134 | H | 1.76253 | 2.211759 | 0.232123 |
| H | 6.364255 | 1.133903 | 1.71551 | H | -0.12289 | -3.43934 | 1.201638 |
| H | 7.72288 | 0.976367 | -0.3517 | H | -0.1285 | -3.04666 | -0.51112 |
| H | 3.024436 | -2.07801 | 1.841164 | H | -2.36332 | -2.74011 | 1.376315 |
| H | 2.477769 | -3.32815 | 0.72616 | H | -2.05843 | -0.45932 | 1.602659 |
| H | 1.725063 | -1.68041 | -0.8785 | H | -4.39536 | -1.87028 | 0.844878 |
| H | -0.47052 | 1.318359 | 1.55302 | H | -4.52085 | -0.33198 | 1.662536 |
| H | 1.760183 | 1.212787 | 1.828674 | H | -3.31869 | -0.64836 | -1.139 |
| H | 1.833244 | 2.176943 | 0.391693 | H | -6.08932 | -2.27308 | -1.34456 |
| H | -0.08692 | -3.49827 | 1.15292 | H | -5.51129 | -0.79899 | -2.14478 |
| H | -0.0796 | -3.06322 | -0.54948 | H | -6.30822 | 0.886301 | 2.345445 |
| H | -2.32498 | -2.79156 | 1.334555 | H | -8.02099 | 0.425975 | 2.239354 |
| H | -2.01763 | -0.51652 | 1.607466 | H | -7.46614 | 1.890423 | 1.448762 |
| H | -4.35761 | -1.89392 | 0.805754 | H | -3.38643 | -2.96671 | -0.57902 |
| H | -4.47345 | -0.37131 | 1.654232 | H | 2.966059 | 1.457771 | -1.7081 |
| H | -3.25797 | -0.64374 | -1.14623 | H | 3.147555 | -0.32684 | -2.01708 |
| H | -6.05584 | -2.24915 | -1.38141 | H | 0.973417 | -2.03323 | 2.735946 |
| H | -5.45177 | -0.77624 | -2.16468 | H | -0.54605 | -1.16604 | 2.763971 |
| H | -6.24325 | 0.875623 | 2.339131 | H | 0.968966 | -0.28043 | 2.641859 |
| H | -7.96396 | 0.449218 | 2.217955 | H | -0.72064 | -1.34802 | -1.84558 |
| H | -7.3758 | 1.910102 | 1.44498 | H | -1.21252 | 0.337845 | -1.92656 |
| H | -3.34132 | -2.96522 | -0.63244 | H | 0.493216 | -0.05286 | -1.87204 |
| H | 6.597371 | 1.238287 | -2.32686 | H | -2.69668 | 1.717738 | -0.90332 |
| H | 3.025771 | 1.49025 | -1.6009 | H | -4.42731 | 1.555915 | -0.58064 |
| H | 3.21836 | -0.28403 | -1.95756 | H | -3.29417 | 1.763784 | 0.758439 |
| H | 1.004185 | -2.13029 | 2.730633 | H | -0.57168 | 4.250446 | -1.82708 |
| H | -0.52956 | -1.28894 | 2.765021 | H | -2.19283 | 4.202871 | -1.13101 |
| H | 0.971622 | -0.37554 | 2.68912 | H | -1.0242 | 5.398668 | -0.52555 |
| H | -0.65118 | -1.34729 | -1.84251 | H | 4.007844 | -2.38249 | -1.03622 |
| H | -1.13871 | 0.340325 | -1.90081 | H | 4.811865 | -2.99194 | 0.399677 |
| H | 0.565637 | -0.054 | -1.83479 | H | 4.993223 | -0.79901 | 1.37389 |
| H | -2.61721 | 1.710981 | -0.86447 | C | 5.873954 | 1.886782 | -0.03159 |
| H | -4.35057 | 1.561282 | -0.55168 | H | 6.335149 | 2.772602 | 0.419051 |
| H | -3.22043 | 1.733313 | 0.795231 | H | 5.77788 | 2.096457 | -1.10677 |
| H | -0.38451 | 4.235918 | -1.77599 | C | 6.812274 | 0.693815 | 0.171499 |
| H | -2.04018 | 4.185966 | -1.16695 | H | 6.975582 | 0.568462 | 1.250702 |
| H | -0.90263 | 5.375299 | -0.49175 | O | 8.114124 | 0.973217 | -0.34637 |
| H | 6.208933 | -2.26125 | -1.84936 | H | 8.007762 | 1.253126 | -1.26545 |
| H | 7.267252 | -0.93612 | -2.29906 | C | 6.194531 | -0.66222 | -1.90007 |
| H | 5.508838 | -0.75311 | -2.4535 | H | 7.210535 | -0.58102 | -2.30103 |
| H | 7.096333 | -2.42478 | 0.54989 | H | 5.781014 | -1.6016 | -2.27892 |
| H | 7.441063 | -0.93727 | 1.437675 | H | 5.59523 | 0.151601 | -2.31574 |
| H | 8.31594 | -1.30054 | -0.05625 | C | 7.17709 | -1.7797 | 0.109106 |
| H | 4.138758 | 2.498095 | 0.17371 | H | 7.13557 | -1.90813 | 1.197373 |
| H | 4.080317 | 1.736048 | 1.75598 | H | 6.932337 | -2.74071 | -0.35313 |
| H | 4.035491 | -2.36686 | -1.02901 | H | 8.206227 | -1.52667 | -0.15835 |
| H | 4.876558 | -3.00653 | 0.371778 | H | 4.558233 | 1.569552 | 1.655574 |
| H | 5.072206 | -0.83802 | 1.388462 | H | 3.843985 | 2.508863 | 0.355181 |
| **Lactone B-1** | | | | **Lactone B-2** | | | |
| C | 6.097982 | -0.84299 | -0.3422 | C | 6.065709 | -0.80596 | -0.33693 |
| C | 4.670352 | -0.93092 | 0.30064 | C | 4.629749 | -0.98611 | 0.264947 |
| C | 3.791219 | 0.292342 | -0.01468 | C | 3.772688 | 0.287472 | 0.165293 |
| C | 4.461471 | 1.568897 | 0.491349 | C | 4.451197 | 1.437631 | 0.906833 |
| C | 3.940536 | -2.26493 | 0.082874 | C | 3.885649 | -2.24821 | -0.19779 |
| C | 2.593655 | -2.25452 | 0.81354 | C | 2.52756 | -2.34932 | 0.503815 |
| C | 1.628695 | -1.24631 | 0.171044 | C | 1.585172 | -1.22507 | 0.044504 |
| C | 2.258648 | 0.167367 | 0.005157 | C | 2.236254 | 0.184135 | 0.143793 |
| C | 0.227387 | -1.18235 | 0.83151 | C | 0.177182 | -1.26743 | 0.692292 |
| C | -0.66265 | -0.08491 | 0.100582 | C | -0.69834 | -0.03975 | 0.172076 |
| C | -0.0875 | 1.289398 | 0.518489 | C | -0.10185 | 1.206085 | 0.876538 |
| C | 1.469049 | 1.336817 | 0.607908 | C | 1.458081 | 1.238234 | 0.944431 |
| C | -0.6015 | -2.46816 | 0.623458 | C | -0.66388 | -2.47873 | 0.241887 |
| C | -2.08711 | -2.04716 | 0.590982 | C | -2.14432 | -2.04908 | 0.310072 |
| C | -2.09561 | -0.46518 | 0.629094 | C | -2.13869 | -0.49378 | 0.620196 |
| C | -5.91111 | -0.3796 | 0.181904 | C | -5.96009 | -0.32674 | 0.26843 |
| C | -4.60322 | -0.30438 | 0.906391 | C | -4.63334 | -0.36324 | 0.960233 |
| C | -3.39565 | 0.090331 | -0.00714 | C | -3.44326 | 0.158761 | 0.087776 |
| C | -6.97974 | 0.437843 | 0.145072 | C | -7.02506 | 0.488004 | 0.383836 |
| C | -8.01462 | -0.17407 | -0.76305 | C | -8.08958 | 0.017651 | -0.57307 |
| O | -7.44806 | -1.40424 | -1.22743 | O | -7.54266 | -1.13006 | -1.23141 |
| C | -6.20701 | -1.55073 | -0.67792 | C | -6.28698 | -1.3573 | -0.74672 |
| C | -7.22416 | 1.733998 | 0.846097 | C | -7.24312 | 1.666976 | 1.274785 |
| O | -2.64371 | -2.60532 | -0.58989 | O | -2.72807 | -2.39627 | -0.93623 |
| C | 3.000215 | 0.398763 | -1.29964 | C | 3.000205 | 0.634541 | -1.08666 |
| C | 0.336401 | -0.95955 | 2.36678 | C | 0.279303 | -1.34143 | 2.243338 |
| C | -0.62271 | -0.23642 | -1.44001 | C | -0.65859 | 0.092488 | -1.36725 |
| C | -3.45286 | 1.603451 | -0.24465 | C | -3.51159 | 1.689294 | 0.058653 |
| O | -0.50664 | 2.315631 | -0.43375 | O | -0.66232 | 2.49119 | 0.440477 |
| C | -0.61016 | 3.578419 | 0.03625 | C | -0.18842 | 3.227941 | -0.58512 |
| C | -1.02169 | 4.530307 | -1.06359 | C | -0.92377 | 4.55101 | -0.65029 |
| O | -0.41119 | 3.897401 | 1.189963 | O | 0.69918 | 2.907999 | -1.34669 |
| H | 2.773405 | -2.01164 | 1.868263 | H | 2.694918 | -2.30472 | 1.587155 |
| H | 2.142682 | -3.2539 | 0.791251 | H | 2.065136 | -3.32152 | 0.294403 |
| H | 1.451607 | -1.63048 | -0.84064 | H | 1.416508 | -1.41602 | -1.02226 |
| H | -0.49315 | 1.584704 | 1.487954 | H | -0.46861 | 1.190559 | 1.905647 |
| H | 1.742042 | 1.457494 | 1.661257 | H | 1.732027 | 1.151297 | 2.002257 |
| H | 1.778316 | 2.26529 | 0.126117 | H | 1.786484 | 2.228701 | 0.638619 |
| H | -0.40462 | -3.21622 | 1.397136 | H | -0.46756 | -3.36834 | 0.847646 |
| H | -0.37838 | -2.93866 | -0.33798 | H | -0.45235 | -2.7477 | -0.79648 |
| H | -2.61849 | -2.43607 | 1.471399 | H | -2.66337 | -2.57835 | 1.122064 |
| H | -2.12874 | -0.17005 | 1.686618 | H | -2.17356 | -0.38562 | 1.712988 |
| H | -4.43065 | -1.28609 | 1.358458 | H | -4.45542 | -1.40216 | 1.255089 |
| H | -4.66711 | 0.410383 | 1.734938 | H | -4.67021 | 0.218957 | 1.888415 |
| H | -3.52608 | -0.40937 | -0.97249 | H | -3.59171 | -0.20382 | -0.93424 |
| H | -6.38102 | 2.015664 | 1.479434 | H | -6.37867 | 1.856051 | 1.913853 |
| H | -8.12218 | 1.675705 | 1.473406 | H | -8.11998 | 1.515134 | 1.916191 |
| H | -7.3938 | 2.544071 | 0.126222 | H | -7.43486 | 2.572768 | 0.686626 |
| H | -3.61698 | -2.60451 | -0.55337 | H | -3.69978 | -2.4164 | -0.87381 |
| H | 2.936542 | 1.379582 | -1.7672 | H | 2.926997 | 1.67889 | -1.37468 |
| H | 3.027311 | -0.42095 | -2.01255 | H | 3.024052 | -0.05085 | -1.93027 |
| H | 0.829127 | -1.82293 | 2.821598 | H | 0.727866 | -2.2969 | 2.527518 |
| H | -0.64407 | -0.88018 | 2.84221 | H | -0.69996 | -1.30706 | 2.727102 |
| H | 0.911009 | -0.07748 | 2.653086 | H | 0.895913 | -0.55877 | 2.688888 |
| H | -0.90754 | -1.24276 | -1.74897 | H | -0.99219 | -0.82774 | -1.84946 |
| H | -1.332 | 0.454251 | -1.89916 | H | -1.34434 | 0.879541 | -1.6894 |
| H | 0.359521 | 0.002669 | -1.8512 | H | 0.327202 | 0.367188 | -1.73982 |
| H | -2.73861 | 1.922239 | -1.00172 | H | -2.83342 | 2.113761 | -0.67956 |
| H | -4.45425 | 1.889685 | -0.58578 | H | -4.52664 | 2.007837 | -0.20549 |
| H | -3.24154 | 2.162553 | 0.675258 | H | -3.25843 | 2.129974 | 1.028523 |
| H | -0.42423 | 4.361457 | -1.96283 | H | -1.99666 | 4.386264 | -0.78403 |
| H | -2.06972 | 4.356347 | -1.32802 | H | -0.79438 | 5.097736 | 0.288332 |
| H | -0.90547 | 5.556531 | -0.71592 | H | -0.53295 | 5.13848 | -1.48053 |
| H | 3.780464 | -2.46354 | -0.98498 | H | 3.739789 | -2.25013 | -1.28602 |
| H | 4.557571 | -3.0858 | 0.460184 | H | 4.486118 | -3.13298 | 0.034477 |
| H | 4.847167 | -0.87839 | 1.389128 | H | 4.795709 | -1.13381 | 1.346708 |
| C | 5.862294 | 1.716879 | -0.11798 | C | 5.867966 | 1.675523 | 0.364371 |
| H | 6.358385 | 2.605756 | 0.296932 | H | 6.3791 | 2.452657 | 0.943826 |
| H | 5.786208 | 1.876674 | -1.19952 | H | 5.801061 | 2.047113 | -0.66818 |
| C | 6.735348 | 0.487277 | 0.145962 | C | 6.723593 | 0.406701 | 0.393282 |
| H | 6.866242 | 0.393195 | 1.239436 | H | 6.860826 | 0.110759 | 1.442426 |
| O | 8.016189 | 0.621984 | -0.47479 | O | 8.049899 | 0.675143 | -0.0679 |
| H | 8.419523 | 1.432444 | -0.13838 | H | 7.974119 | 1.087079 | -0.93908 |
| C | 6.073667 | -0.89912 | -1.88367 | C | 6.051559 | -0.57907 | -1.86227 |
| H | 7.091601 | -0.81988 | -2.27213 | H | 7.075665 | -0.4904 | -2.24172 |
| H | 5.652926 | -1.84815 | -2.23059 | H | 5.597304 | -1.42849 | -2.38092 |
| H | 5.483308 | -0.09328 | -2.32499 | H | 5.497757 | 0.316485 | -2.15381 |
| C | 6.98467 | -1.99748 | 0.172574 | C | 6.932491 | -2.04657 | -0.0384 |
| H | 6.945223 | -2.07276 | 1.266236 | H | 6.868851 | -2.32987 | 1.019171 |
| H | 6.677275 | -2.96178 | -0.24223 | H | 6.631663 | -2.91149 | -0.63701 |
| H | 8.023844 | -1.8228 | -0.11618 | H | 7.979283 | -1.82531 | -0.26231 |
| H | 4.541672 | 1.531097 | 1.587686 | H | 4.511963 | 1.198246 | 1.979045 |
| H | 3.871668 | 2.458133 | 0.255095 | H | 3.878048 | 2.364804 | 0.826711 |
| H | -8.24511 | 0.458734 | -1.62949 | H | -8.34353 | 0.771712 | -1.32883 |
| H | -8.95599 | -0.3931 | -0.24322 | H | -9.01565 | -0.27613 | -0.06268 |
| O | -5.51917 | -2.52717 | -0.89768 | O | -5.61117 | -2.29119 | -1.12881 |
| **Lactone B-3** | | | | **Lactone B-4** | | | |
| C | 6.021432 | 1.471947 | 0.671406 | C | 6.097982 | -0.84299 | -0.3422 |
| C | 6.536936 | 0.660384 | -0.52241 | C | 4.670352 | -0.93092 | 0.30064 |
| C | 6.112605 | -0.84563 | -0.40425 | C | 3.791219 | 0.292342 | -0.01468 |
| C | 4.722155 | -0.9505 | 0.330762 | C | 4.461471 | 1.568897 | 0.491349 |
| C | 3.832067 | 0.278961 | 0.085414 | C | 3.940536 | -2.26493 | 0.082874 |
| C | 4.475299 | 1.543247 | 0.683312 | C | 2.593655 | -2.25452 | 0.81354 |
| C | 3.985296 | -2.28497 | 0.140664 | C | 1.628695 | -1.24631 | 0.171044 |
| C | 2.653109 | -2.27422 | 0.897937 | C | 2.258648 | 0.167367 | 0.005157 |
| C | 1.682957 | -1.27699 | 0.244399 | C | 0.227387 | -1.18235 | 0.83151 |
| C | 2.306775 | 0.141073 | 0.102759 | C | -0.66265 | -0.08491 | 0.100582 |
| C | 0.266819 | -1.22546 | 0.876123 | C | -0.0875 | 1.289398 | 0.518489 |
| C | -0.60356 | -0.10908 | 0.150083 | C | 1.469049 | 1.336817 | 0.607908 |
| C | -0.02912 | 1.261747 | 0.59061 | C | -0.6015 | -2.46816 | 0.623458 |
| C | 1.520355 | 1.285025 | 0.747941 | C | -2.08711 | -2.04716 | 0.590982 |
| C | -0.56285 | -2.50201 | 0.620117 | C | -2.09561 | -0.46518 | 0.629094 |
| C | -2.04645 | -2.07054 | 0.581465 | C | -5.91111 | -0.3796 | 0.181904 |
| C | -2.04555 | -0.48876 | 0.650165 | C | -4.60322 | -0.30438 | 0.906391 |
| C | -5.8512 | -0.36104 | 0.14558 | C | -3.39565 | 0.090331 | -0.00714 |
| C | -4.55504 | -0.3058 | 0.892667 | C | -6.97974 | 0.437843 | 0.145072 |
| C | -3.32956 | 0.09132 | 0.004615 | C | -8.01462 | -0.17407 | -0.76305 |
| C | -6.9107 | 0.467691 | 0.097935 | O | -7.44806 | -1.40424 | -1.22743 |
| C | -7.93588 | -0.12548 | -0.83335 | C | -6.20701 | -1.55073 | -0.67792 |
| O | -7.37391 | -1.35748 | -1.29872 | C | -7.22416 | 1.733998 | 0.846097 |
| C | -6.14412 | -1.52131 | -0.72972 | O | -2.64371 | -2.60532 | -0.58989 |
| C | -7.15402 | 1.76007 | 0.806267 | C | 3.000215 | 0.398763 | -1.29964 |
| O | -2.595 | -2.60269 | -0.61458 | C | 0.336401 | -0.95955 | 2.36678 |
| O | 6.146419 | 1.276431 | -1.7515 | C | -0.62271 | -0.23642 | -1.44001 |
| C | 3.032355 | 0.42223 | -1.20185 | C | -3.45286 | 1.603451 | -0.24465 |
| C | 0.335336 | -1.04834 | 2.420099 | O | -0.50664 | 2.315631 | -0.43375 |
| C | -0.54469 | -0.24319 | -1.39249 | C | -0.61016 | 3.578419 | 0.03625 |
| C | -3.36291 | 1.609555 | -0.2043 | C | -1.02169 | 4.530307 | -1.06359 |
| O | -0.3899 | 2.287208 | -0.38537 | O | -0.41119 | 3.897401 | 1.189963 |
| C | -0.49358 | 3.555195 | 0.072145 | H | 2.773405 | -2.01164 | 1.868263 |
| C | -0.84684 | 4.505125 | -1.04908 | H | 2.142682 | -3.2539 | 0.791251 |
| O | -0.3374 | 3.877942 | 1.231216 | H | 1.451607 | -1.63048 | -0.84064 |
| C | 6.108012 | -1.48806 | -1.80519 | H | -0.49315 | 1.584704 | 1.487954 |
| C | 7.173598 | -1.57423 | 0.449179 | H | 1.742042 | 1.457494 | 1.661257 |
| O | -5.46227 | -2.5027 | -0.94664 | H | 1.778316 | 2.26529 | 0.126117 |
| H | 6.44173 | 2.48183 | 0.63396 | H | -0.40462 | -3.21622 | 1.397136 |
| H | 6.385148 | 1.015472 | 1.599411 | H | -0.37838 | -2.93866 | -0.33798 |
| H | 7.632349 | 0.699334 | -0.54591 | H | -2.61849 | -2.43607 | 1.471399 |
| H | 4.618865 | -3.10487 | 0.497606 | H | -2.12874 | -0.17005 | 1.686618 |
| H | 3.794235 | -2.48552 | -0.92067 | H | -4.43065 | -1.28609 | 1.358458 |
| H | 2.842295 | -2.0128 | 1.946579 | H | -4.66711 | 0.410383 | 1.734938 |
| H | 2.206448 | -3.27569 | 0.895187 | H | -3.52608 | -0.40937 | -0.97249 |
| H | 1.529021 | -1.65741 | -0.77252 | H | -6.38102 | 2.015664 | 1.479434 |
| H | -0.47226 | 1.568163 | 1.5403 | H | -8.12218 | 1.675705 | 1.473406 |
| H | 1.752224 | 1.324486 | 1.817054 | H | -7.3938 | 2.544071 | 0.126222 |
| H | 1.870024 | 2.239228 | 0.350679 | H | -3.61698 | -2.60451 | -0.55337 |
| H | -0.38002 | -3.27223 | 1.375411 | H | 2.936542 | 1.379582 | -1.7672 |
| H | -0.3286 | -2.94633 | -0.35092 | H | 3.027311 | -0.42095 | -2.01255 |
| H | -2.58853 | -2.47243 | 1.449411 | H | 0.829127 | -1.82293 | 2.821598 |
| H | -2.09277 | -0.21225 | 1.712081 | H | -0.64407 | -0.88018 | 2.84221 |
| H | -4.62712 | 0.398347 | 1.729546 | H | 0.911009 | -0.07748 | 2.653086 |
| H | -4.39725 | -1.29487 | 1.334101 | H | -0.90754 | -1.24276 | -1.74897 |
| H | -3.45086 | -0.3892 | -0.97154 | H | -1.332 | 0.454251 | -1.89916 |
| H | -8.88834 | -0.33926 | -0.33195 | H | 0.359521 | 0.002669 | -1.8512 |
| H | -8.14446 | 0.517167 | -1.69805 | H | -2.73861 | 1.922239 | -1.00172 |
| H | -7.3043 | 2.577877 | 0.090862 | H | -4.45425 | 1.889685 | -0.58578 |
| H | -8.06261 | 1.705066 | 1.418443 | H | -3.24154 | 2.162553 | 0.675258 |
| H | -6.31858 | 2.028214 | 1.455489 | H | -0.42423 | 4.361457 | -1.96283 |
| H | -3.56868 | -2.59825 | -0.58654 | H | -2.06972 | 4.356347 | -1.32802 |
| H | 5.183845 | 1.212578 | -1.81423 | H | -0.90547 | 5.556531 | -0.71592 |
| H | 3.056028 | -0.37728 | -1.93815 | H | 3.780464 | -2.46354 | -0.98498 |
| H | 2.929105 | 1.416353 | -1.63553 | H | 4.557571 | -3.0858 | 0.460184 |
| H | 0.855043 | -1.90552 | 2.856423 | H | 4.847167 | -0.87839 | 1.389128 |
| H | -0.65797 | -1.02913 | 2.874537 | C | 5.862294 | 1.716879 | -0.11798 |
| H | 0.861697 | -0.15271 | 2.752156 | H | 6.358385 | 2.605756 | 0.296932 |
| H | -0.8369 | -1.24223 | -1.7172 | H | 5.786208 | 1.876674 | -1.19952 |
| H | 0.444289 | -0.01013 | -1.78977 | C | 6.735348 | 0.487277 | 0.145962 |
| H | -1.24032 | 0.460275 | -1.85252 | H | 6.866242 | 0.393195 | 1.239436 |
| H | -3.15347 | 2.147816 | 0.728336 | O | 8.016189 | 0.621984 | -0.47479 |
| H | -2.63622 | 1.932601 | -0.94783 | H | 7.868425 | 0.817999 | -1.40883 |
| H | -4.35605 | 1.917602 | -0.55033 | C | 6.073667 | -0.89912 | -1.88367 |
| H | -0.71453 | 5.531735 | -0.70839 | H | 7.091601 | -0.81988 | -2.27213 |
| H | -0.22874 | 4.311388 | -1.929 | H | 5.652926 | -1.84815 | -2.23059 |
| H | -1.89135 | 4.355799 | -1.34143 | H | 5.483308 | -0.09328 | -2.32499 |
| H | 5.972518 | -2.57174 | -1.73822 | C | 6.98467 | -1.99748 | 0.172574 |
| H | 7.062236 | -1.30048 | -2.30725 | H | 6.945223 | -2.07276 | 1.266236 |
| H | 5.324666 | -1.08546 | -2.45057 | H | 6.677275 | -2.96178 | -0.24223 |
| H | 8.140396 | -1.5935 | -0.06558 | H | 8.023844 | -1.8228 | -0.11618 |
| H | 7.321234 | -1.08689 | 1.419218 | H | 4.541672 | 1.531097 | 1.587686 |
| H | 6.883984 | -2.61234 | 0.643296 | H | 3.871668 | 2.458133 | 0.255095 |
| H | 4.148358 | 2.429815 | 0.131177 | H | -8.24511 | 0.458734 | -1.62949 |
| H | 4.13439 | 1.685885 | 1.716172 | H | -8.95599 | -0.3931 | -0.24322 |
| H | 4.952015 | -0.90604 | 1.407382 | O | -5.51917 | -2.52717 | -0.89768 |

**Table S9.** Computed optical rotation considering the solvation effect of Lactone **A** (**4A**) and Lactone **B** (**4B**).

|  | Calc. | Exptl. |
| --- | --- | --- |
| Lactone **A** (**4A**) | 3.65 | -51.22 |
| Lactone **B** (**4B**) | -58.53 |

**Table S10.** The computed ^13^C NMR data for Lactone **A** (**4A**) (The serial numbers of carbon atoms can be seen in Figure S5)

|  | *δ*exp. | *δ*scal.calc. | Δ*δ* |
| --- | --- | --- | --- |
| 1 | 41.52 | 44.86317 | 3.34317 |
| 2 | 47.67 | 49.81793 | 2.147928 |
| 3 | 26.55 | 30.07226 | 3.522256 |
| 4 | 32.81 | 34.51919 | 1.709194 |
| 5 | 21.67 | 24.57699 | 2.906985 |
| 6 | 26.52 | 28.77862 | 2.258616 |
| 7 | 47.49 | 50.75109 | 3.261088 |
| 8 | 19.91 | 25.29739 | 5.387389 |
| 9 | 50.08 | 52.73194 | 2.651937 |
| 10 | 48.8 | 54.87507 | **6.075073** |
| 11 | 77.08 | 77.53743 | 0.457434 |
| 12 | 38.1 | 40.94909 | 2.849092 |
| 13 | 48.66 | 47.40989 | -1.25011 |
| 14 | 72.05 | 74.20772 | 2.157715 |
| 15 | 58.57 | 60.27094 | 1.700944 |
| 16 | 159.71 | 157.6236 | -2.08639 |
| 17 | 31.21 | 37.13205 | 5.922048 |
| 18 | 31.24 | 34.25928 | 3.019282 |
| 19 | 127.06 | 124.3701 | -2.68991 |
| 20 | 177.33 | 167.8602 | **-9.46976** |
| 22 | 73.57 | 70.13841 | -3.43159 |
| 24 | 12.84 | 11.47819 | -1.36181 |
| 26 | 30.28 | 31.84359 | 1.563592 |
| 27 | 21.03 | 21.82719 | 0.797194 |
| 28 | 14.24 | 15.53303 | 1.293027 |
| 29 | 18.39 | 18.24059 | -0.14941 |
| 31 | 171.17 | 165.5486 | -5.62136 |
| 32 | 22.24 | 21.94812 | -0.29188 |
| 70 | 31.69 | 33.8776 | 1.878576 |
| 73 | 78.33 | 78.2446 | 0.050064 |
| 77 | 15.35 | 16.2409 | 0.567757 |
| 81 | 26.64 | 27.1293 | 0.379943 |

**Table S11.** The computed ^13^C NMR data for Lactone **B** (**4B**) (The serial numbers of carbon atoms can be seen in Figure S5)

|  | *δ*exp. | *δ*scal.calc. | Δ*δ* |
| --- | --- | --- | --- |
| 1 | 41.52 | 45.21221 | 3.673856 |
| 2 | 47.67 | 50.01712 | 2.32951 |
| 3 | 26.55 | 30.14173 | 3.571548 |
| 4 | 32.81 | 34.37301 | 1.543597 |
| 5 | 21.67 | 24.60548 | 2.91471 |
| 6 | 26.52 | 28.88337 | 2.343185 |
| 7 | 47.49 | 50.50198 | 2.994356 |
| 8 | 19.91 | 25.31662 | 5.385636 |
| 9 | 50.08 | 53.47016 | 3.372846 |
| 10 | 48.8 | 54.331 | 5.513536 |
| 11 | 77.08 | 77.73042 | 0.636393 |
| 12 | 38.1 | 41.28629 | 3.167515 |
| 13 | 48.66 | 48.0995 | -0.57798 |
| 14 | 72.05 | 73.0702 | 1.005561 |
| 15 | 58.57 | 60.11666 | 1.530378 |
| 16 | 127.06 | 125.0663 | -2.00165 |
| 17 | 31.21 | 33.19818 | 1.968571 |
| 18 | 31.24 | 34.59697 | 3.337362 |
| 19 | 159.71 | 158.2428 | -1.47114 |
| 20 | 73.57 | 72.47373 | -1.11073 |
| 22 | 177.33 | 171.3521 | -5.97968 |
| 23 | 12.84 | 14.13909 | 1.277244 |
| 25 | 30.28 | 31.72671 | 1.426989 |
| 26 | 21.03 | 22.11415 | 1.063298 |
| 27 | 14.24 | 15.81497 | 1.553289 |
| 28 | 18.39 | 19.43847 | 1.027302 |
| 30 | 171.17 | 165.5596 | -5.61299 |
| 31 | 22.24 | 22.18873 | -0.07197 |
| 67 | 31.69 | 33.72447 | 2.014919 |
| 70 | 78.33 | 78.1226 | -0.22127 |
| 74 | 15.35 | 15.99591 | 0.624368 |
| 78 | 26.64 | 27.23625 | 0.57608 |

**Note:** From the NMR and OR data, we can see structure Lactone **B** matches with the experimental data.

## 6. NMR, MS, IR, CD, and UV spectra of cimyunnins A-D (1-4)

**Figure S7.** ^1^H (600 Hz) NMR Spectrum of cimyunnin **A** (**1**) in Pyridine-*d*_5_


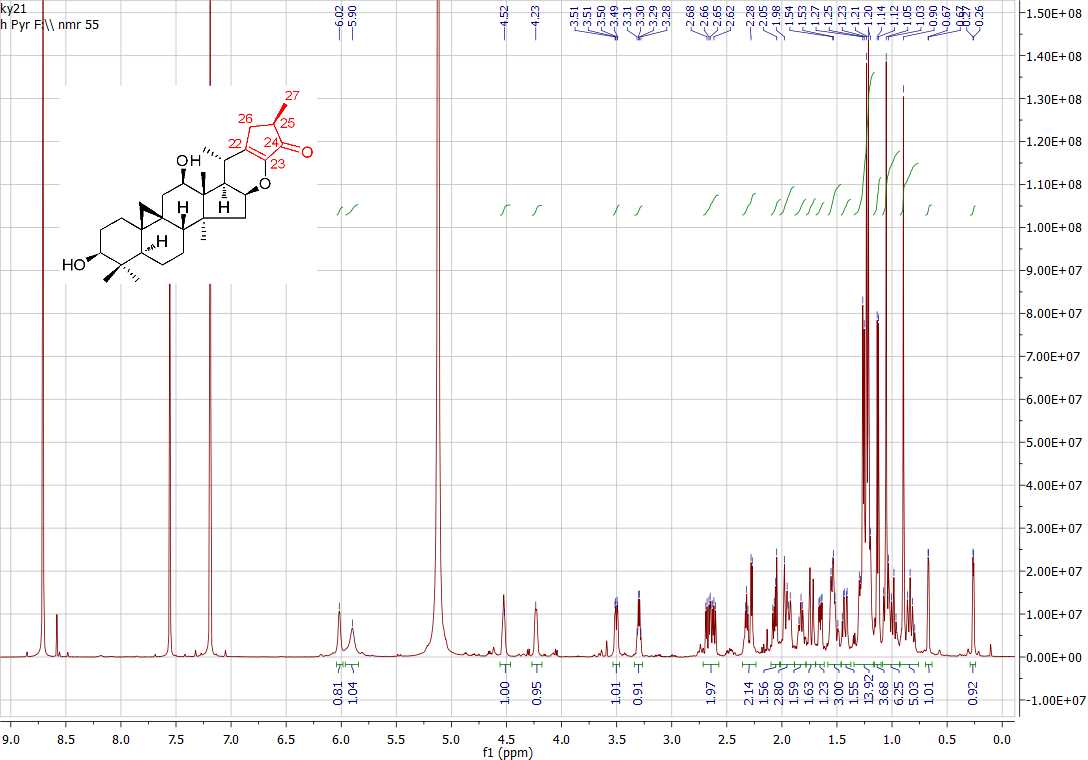


**Figure S8**. Expansions of ^1^H (600 Hz) NMR Spectrum of cimyunnin **A** (**1**) in Pyridine-*d*_5_


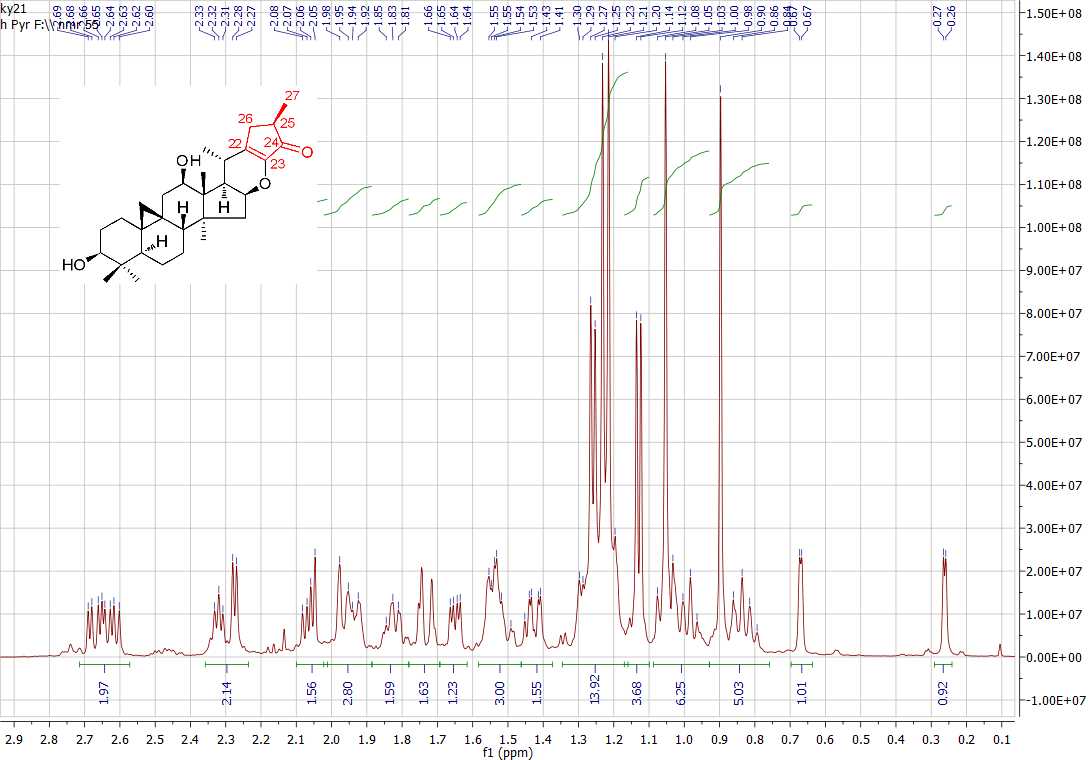


**Figure S9.** ^13^C (150 Hz) NMR Spectrum of cimyunnin **A** (**1**) in Pyridine-*d*_5_


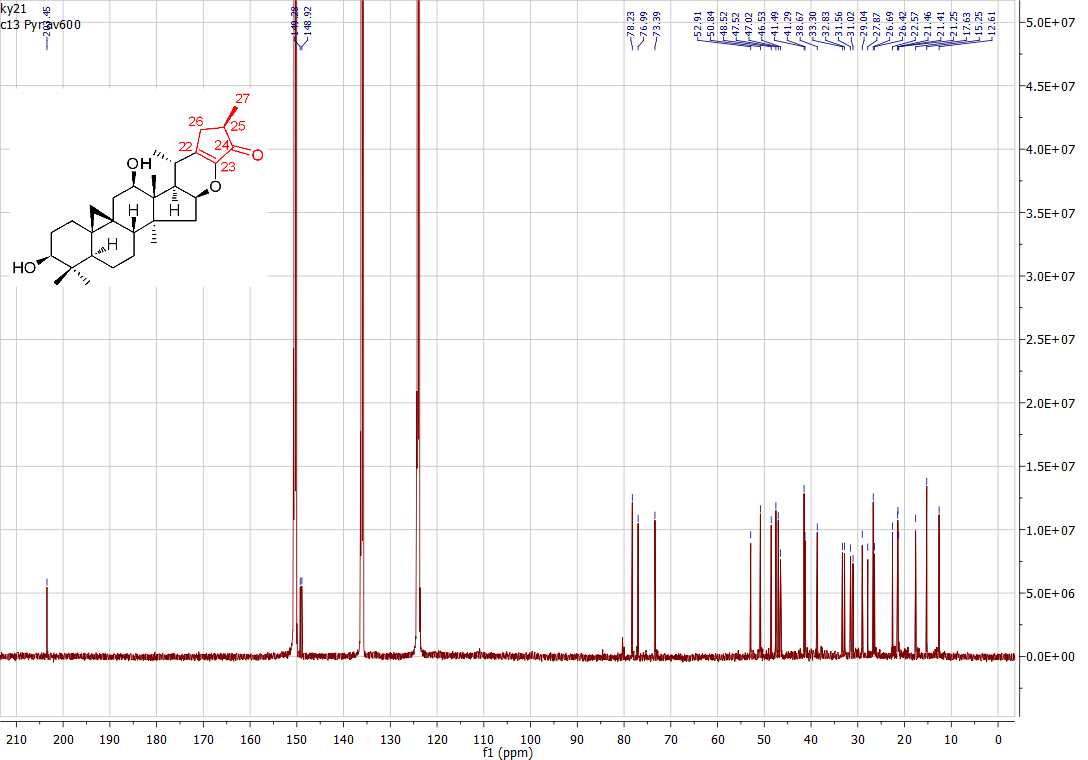


**Figure S10.** Expansions of ^13^C (150 Hz) NMR Spectrum of cimyunnin **A** (**1**) in Pyridine-*d*_5_


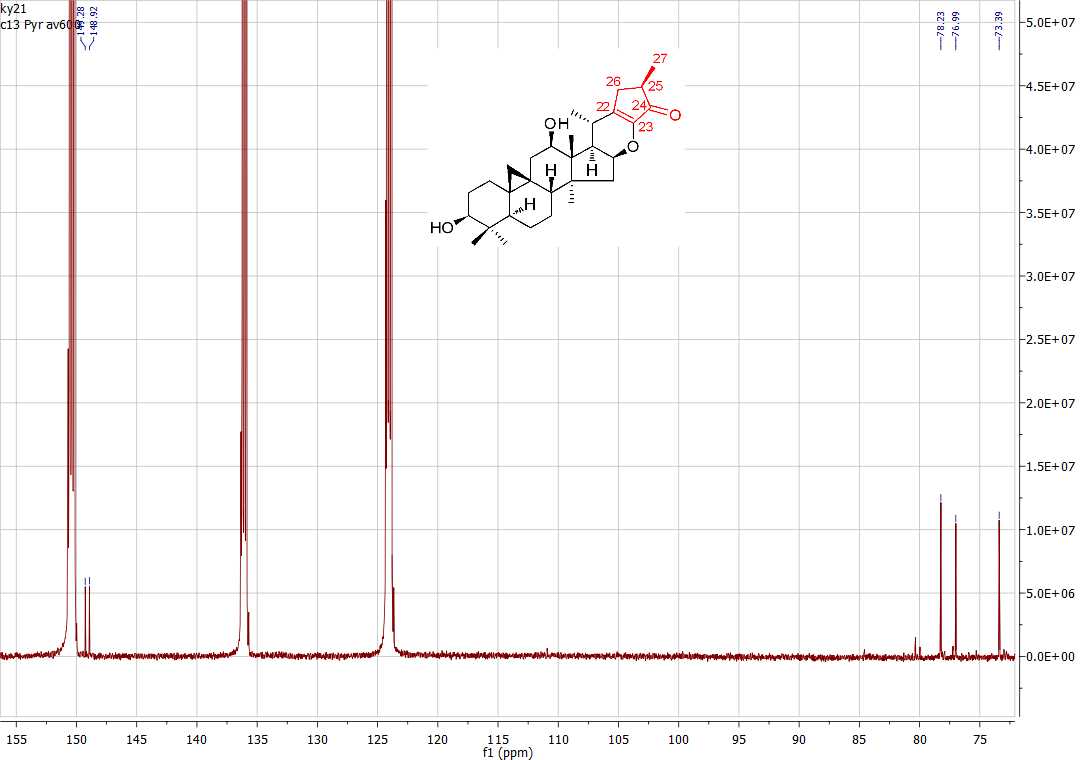


**Figure S11.** Expansions of ^13^C (150 Hz) NMR Spectrum of cimyunnin **A** (**1**) in Pyridine-*d*_5_


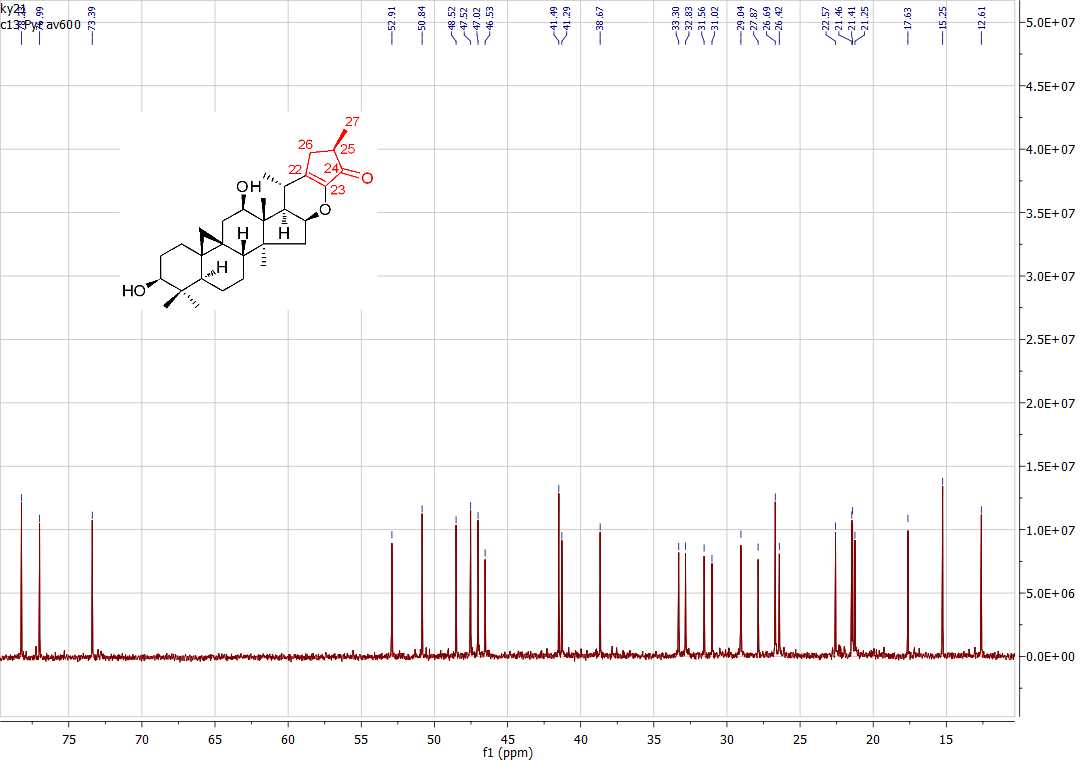


**Figure S12.** HSQC (600 Hz) Spectrum of cimyunnin **A** (**1**) in Pyridine-*d*_5_


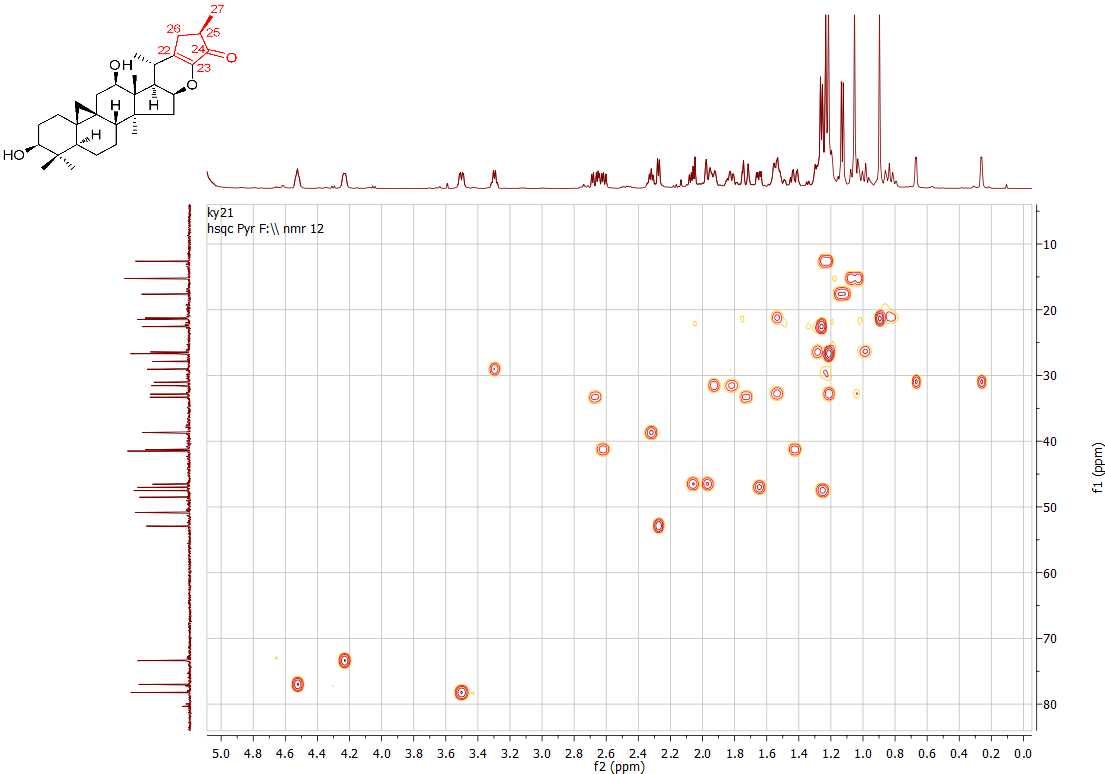


**Figure S13.** HMBC (600 Hz) Spectrum of cimyunnin **A** (**1**) in Pyridine-*d*_5_


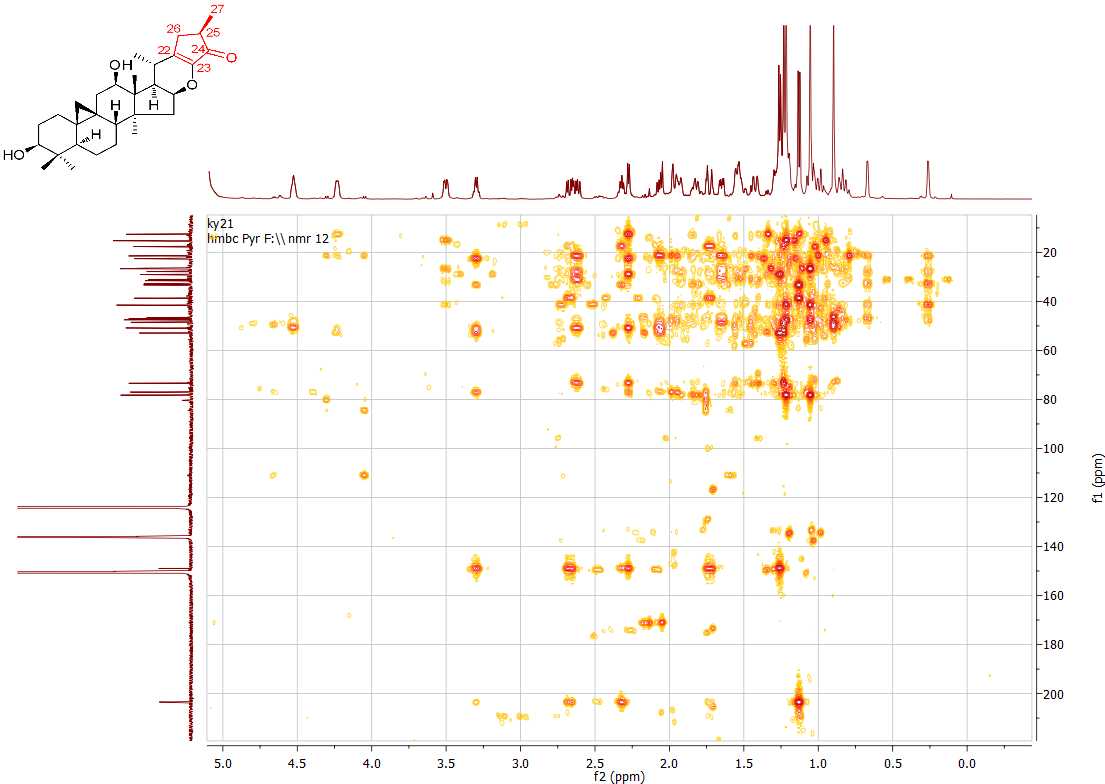


**Figure S14.** Expansions of HMBC (600 Hz) Spectrum of cimyunnin **A** (**1**) in Pyridine-*d*_5_


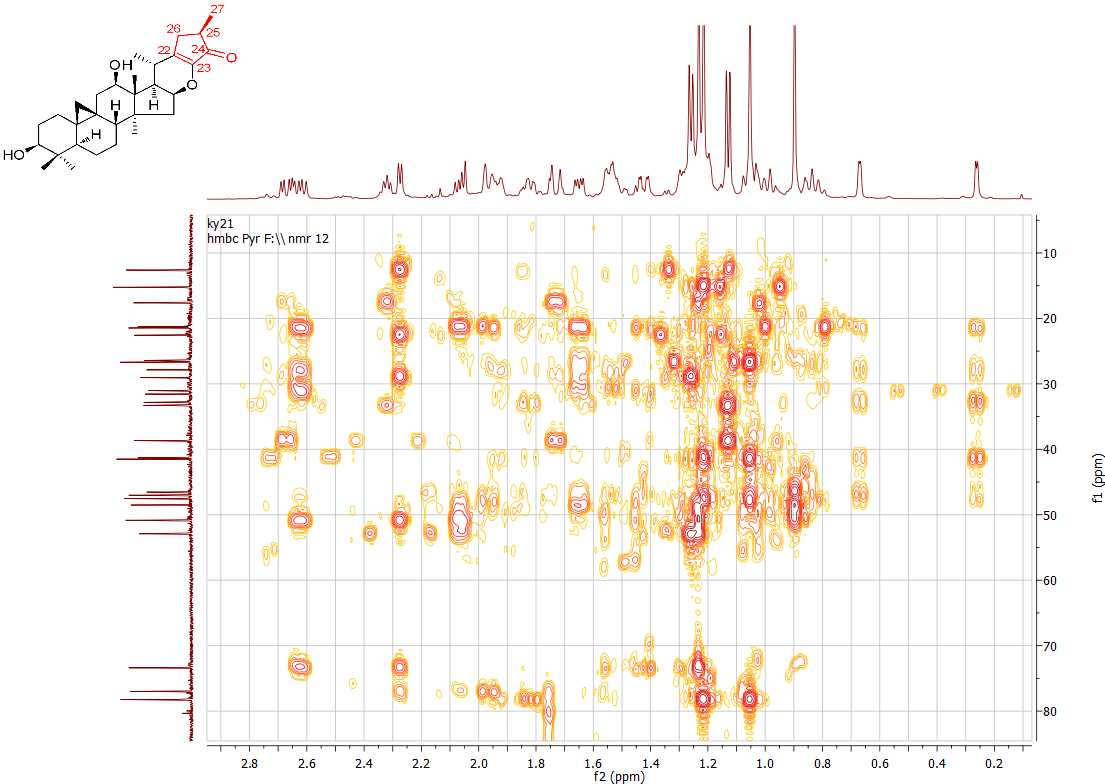


**Figure S15.** Expansions of HMBC (600 Hz) Spectrum of cimyunnin **A** (**1**) in Pyridine-*d*_5_


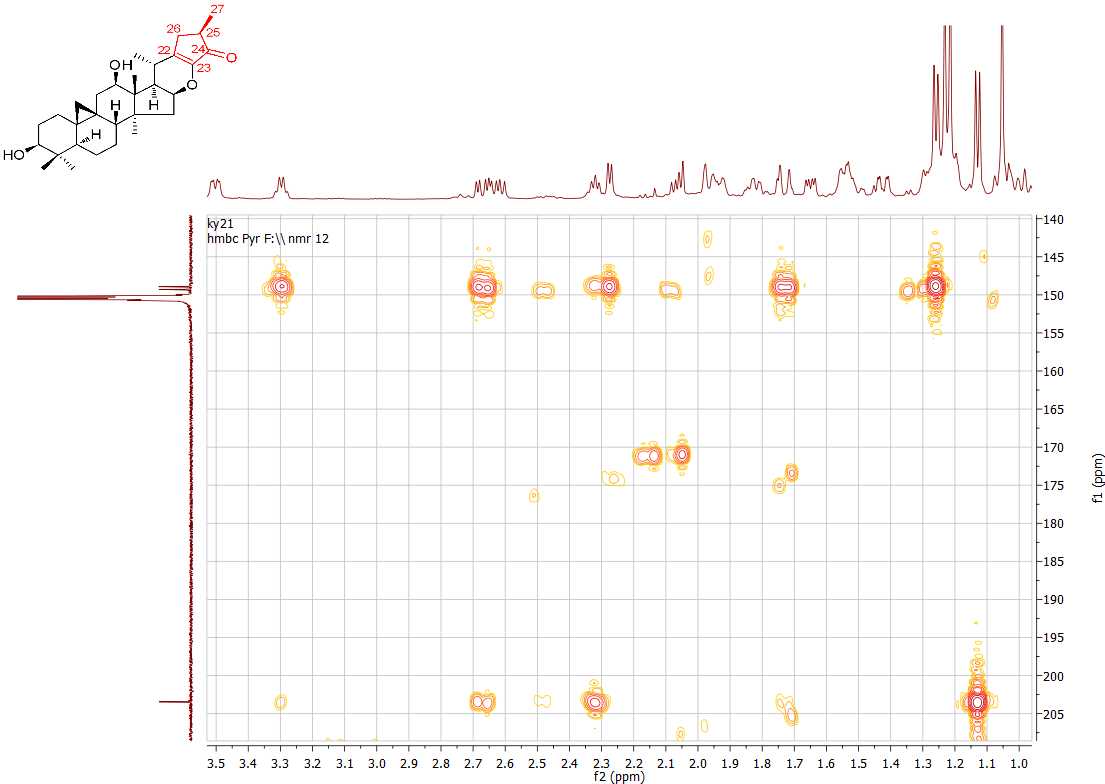


**Figure S16.** Expansions of HMBC (600 Hz) Spectrum of cimyunnin **A** (**1**) in Pyridine-*d*_5_


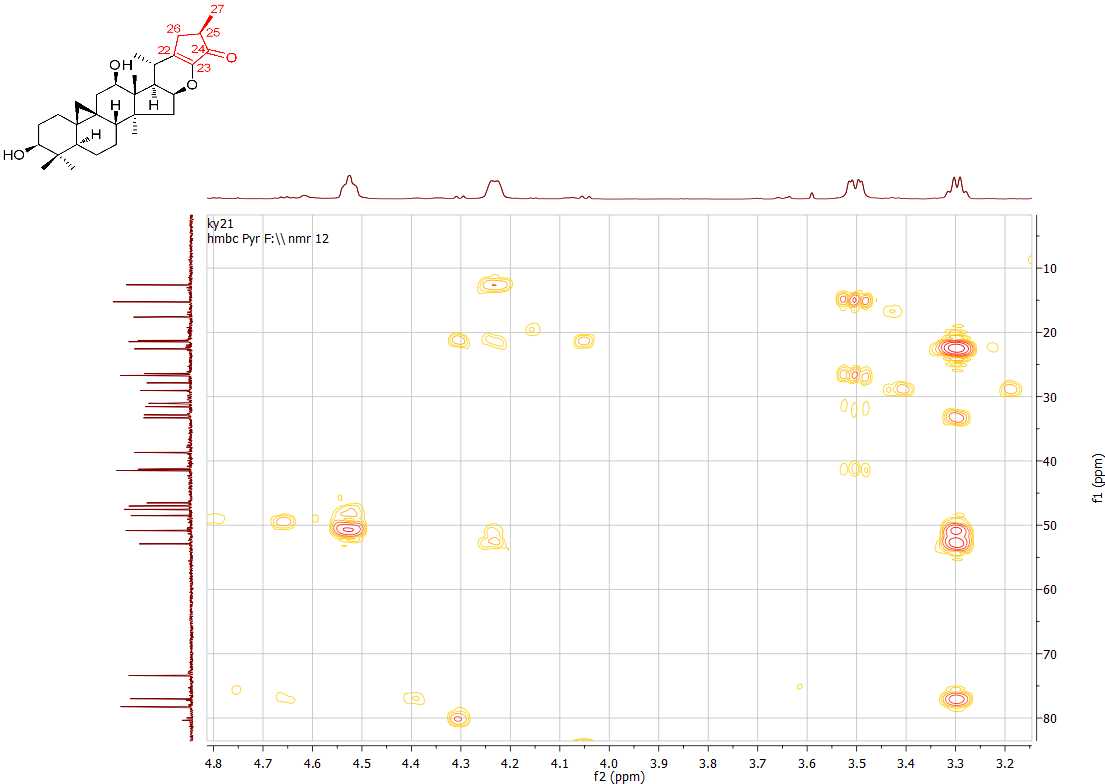


**Figure S17.** ^1^H-^1^H COSY (600 Hz) Spectrum of cimyunnin **A** (**1**) in Pyridine-*d*_5_


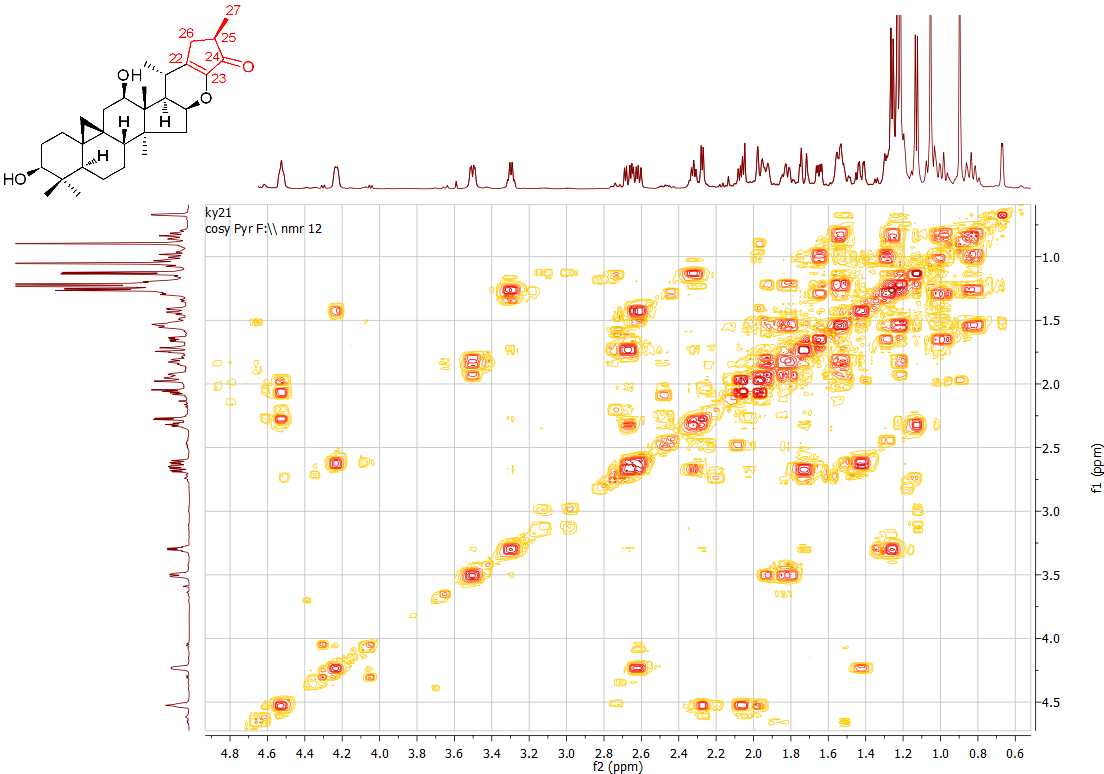


**Figure S18.** ROESY (600 Hz) Spectrum of cimyunnin **A** (**1**) in Pyridine-*d*_5_


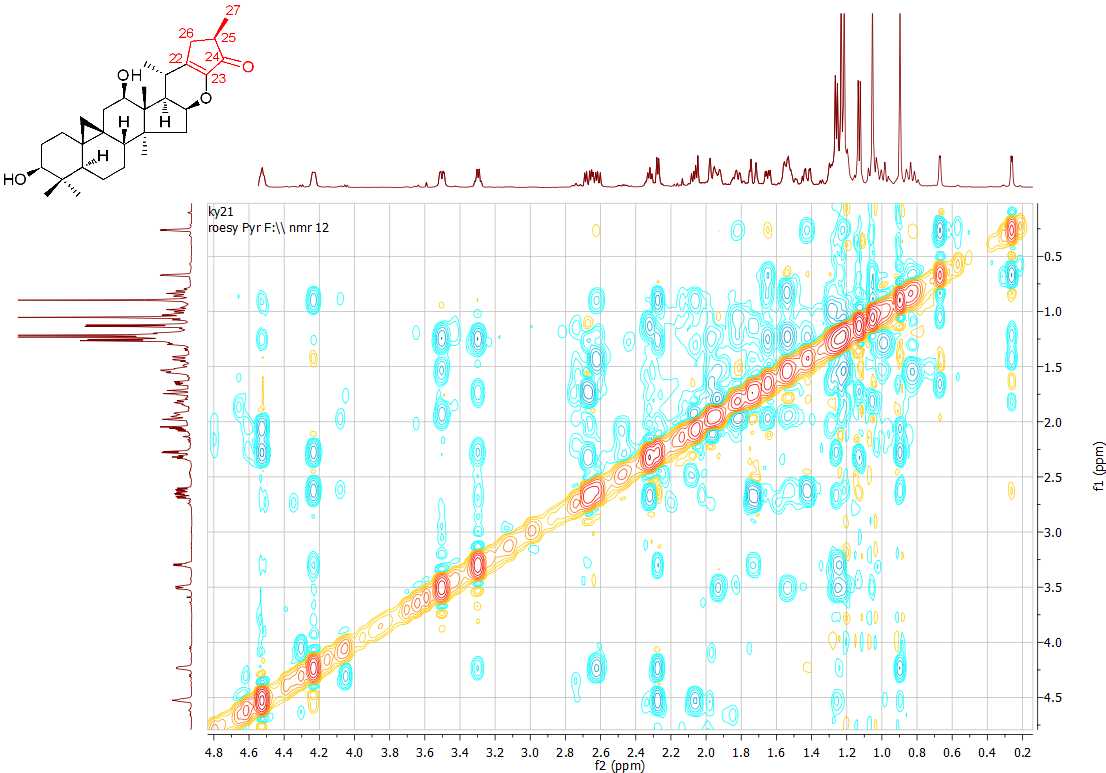


**Figure S19.** HREIMS of cimyunnin **A** (**1**)


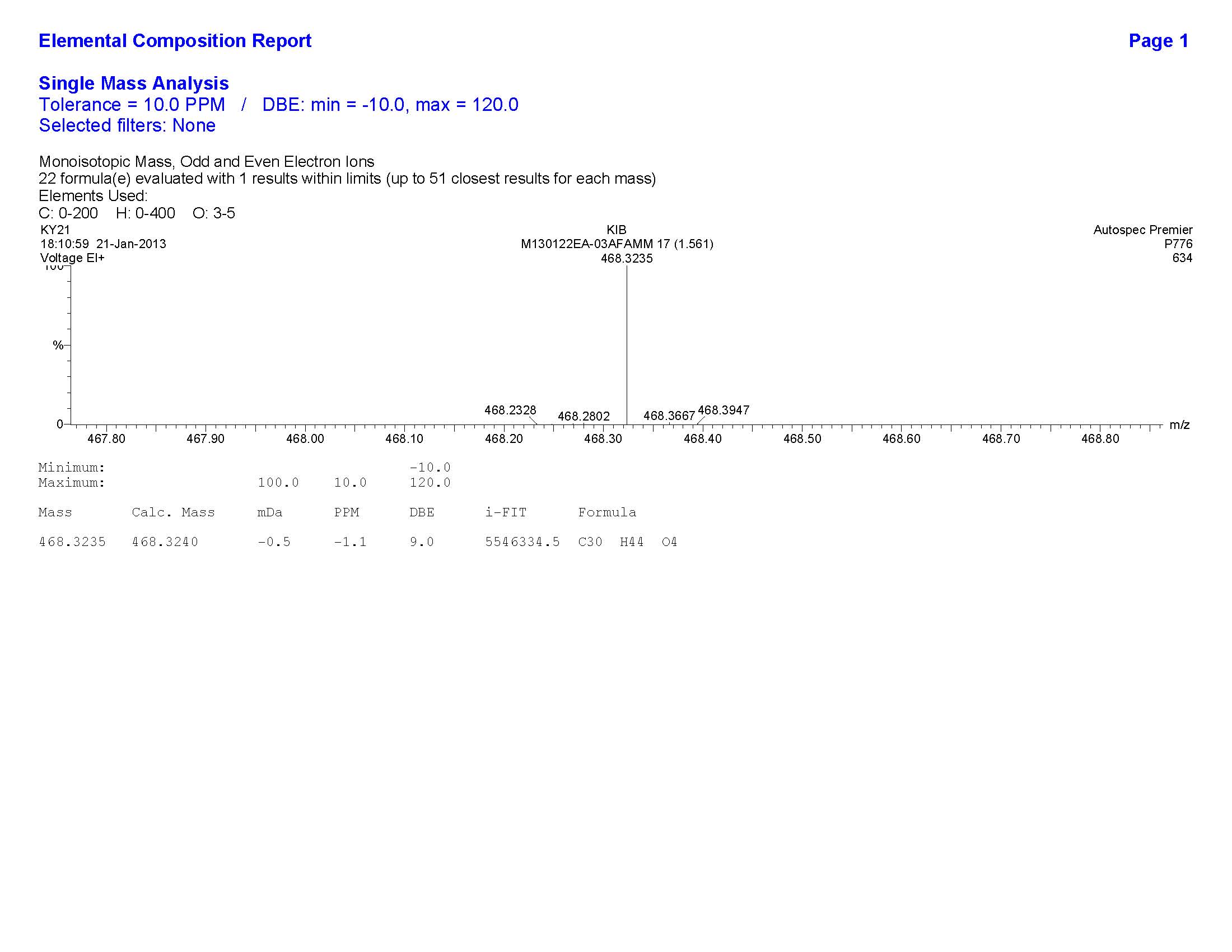


**Figure S20**. IR of cimyunnin **A** (**1**)

**Figure S21.** CD and UV of cimyunnin **A** (**1**)


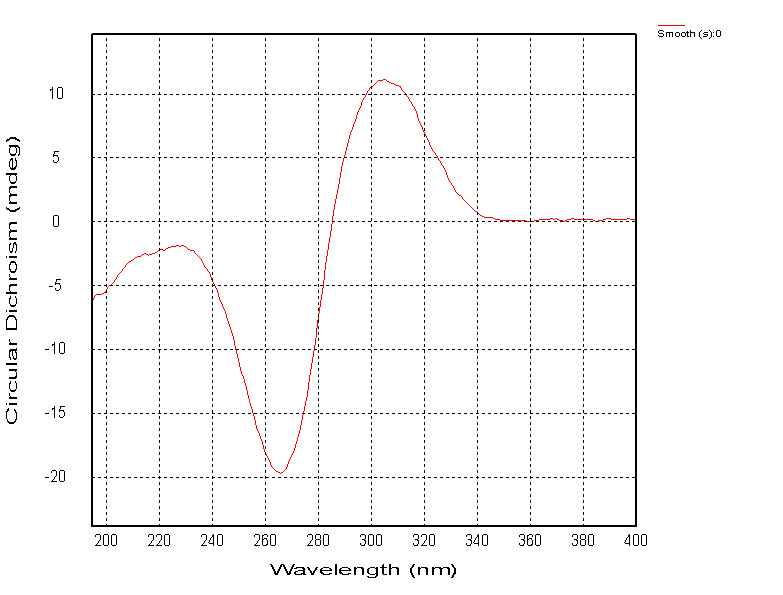

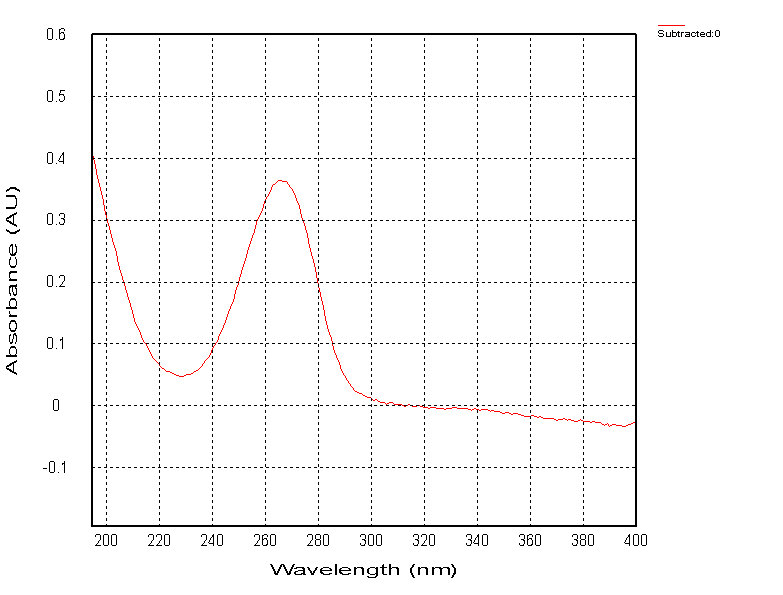


**Figure S22**. ^1^H (500 Hz) NMR Spectrum of cimyunnins **B** and **C** (**2** and **3**) in DMSO-*d*_6_


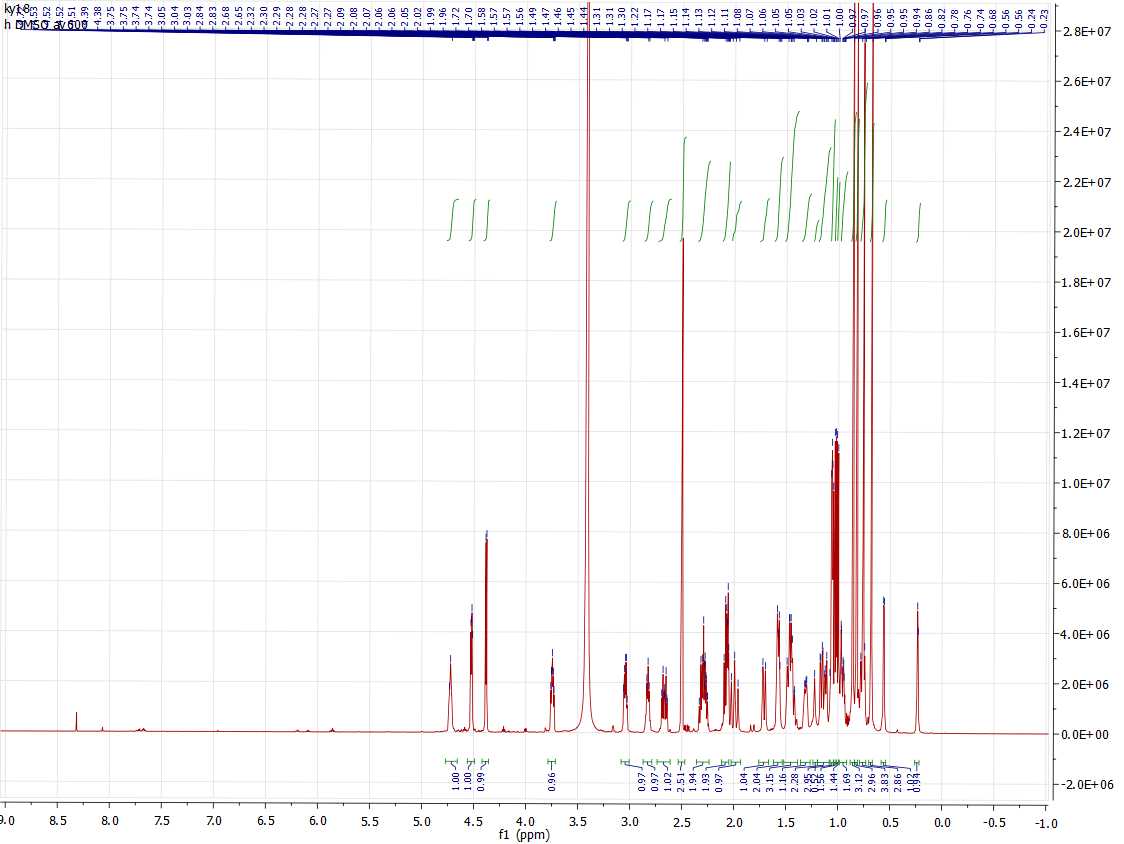


**Figure S23**. Expansions of ^1^H (500 Hz) NMR Spectrum of cimyunnins **B** and **C** (**2** and **3**) in DMSO-*d*_6_


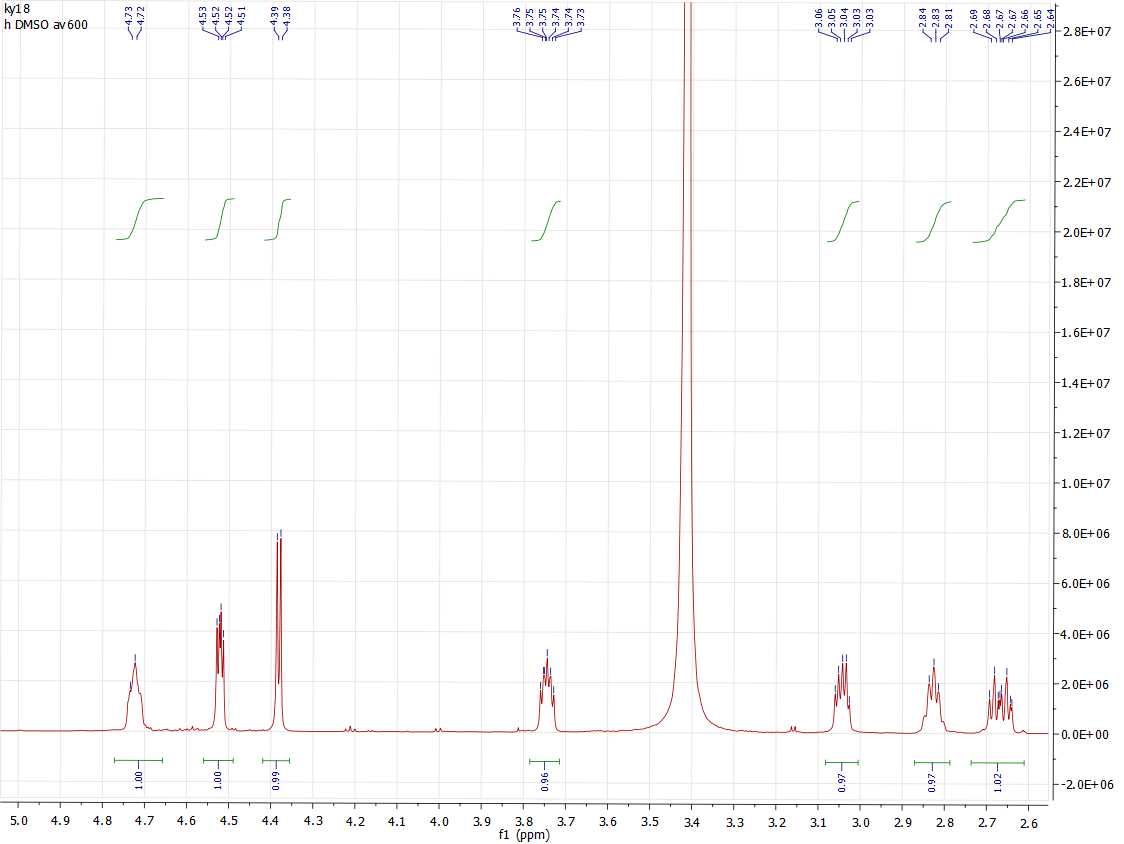


**Figure S24**. Expansions of ^1^H (500 Hz) NMR Spectrum of cimyunnins **B** and **C** (**2** and **3**) in DMSO-*d*_6_


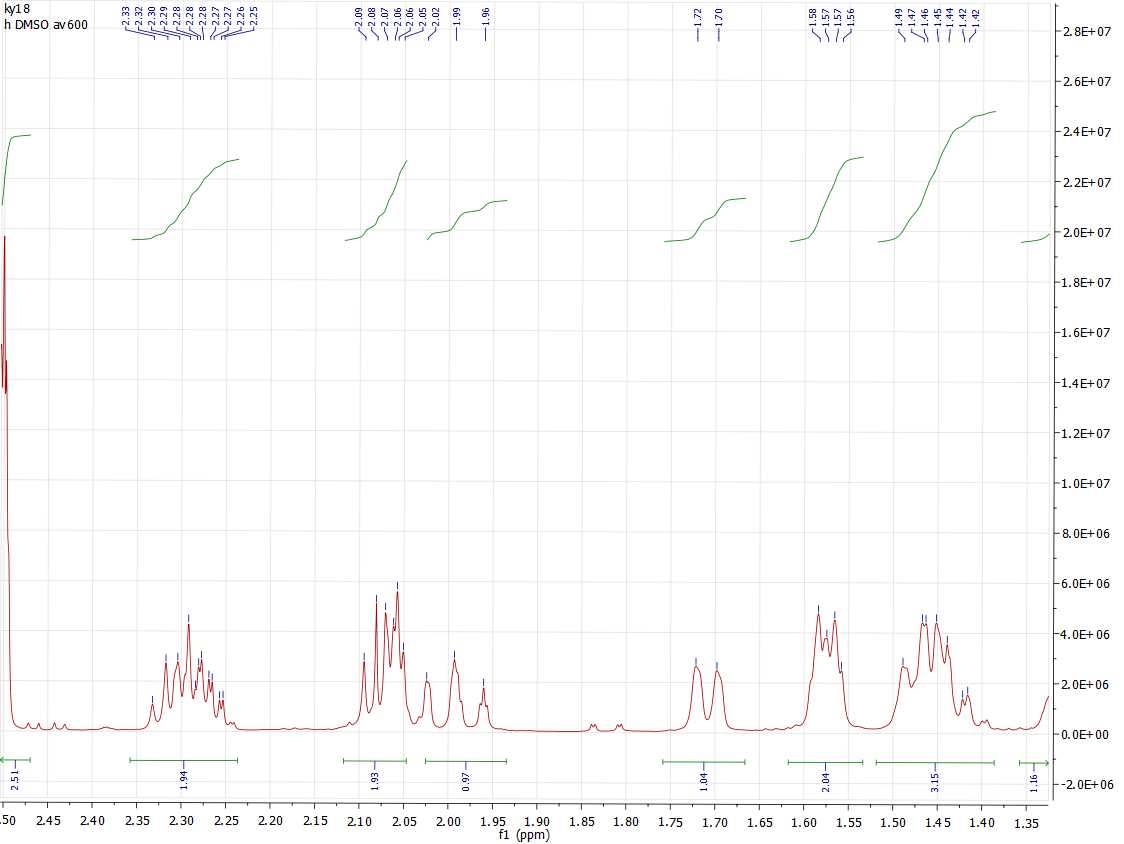


**Figure S25**. Expansions of ^1^H (500 Hz) NMR Spectrum of cimyunnins **B** and **C** (**2** and **3**) in DMSO-*d*_6_


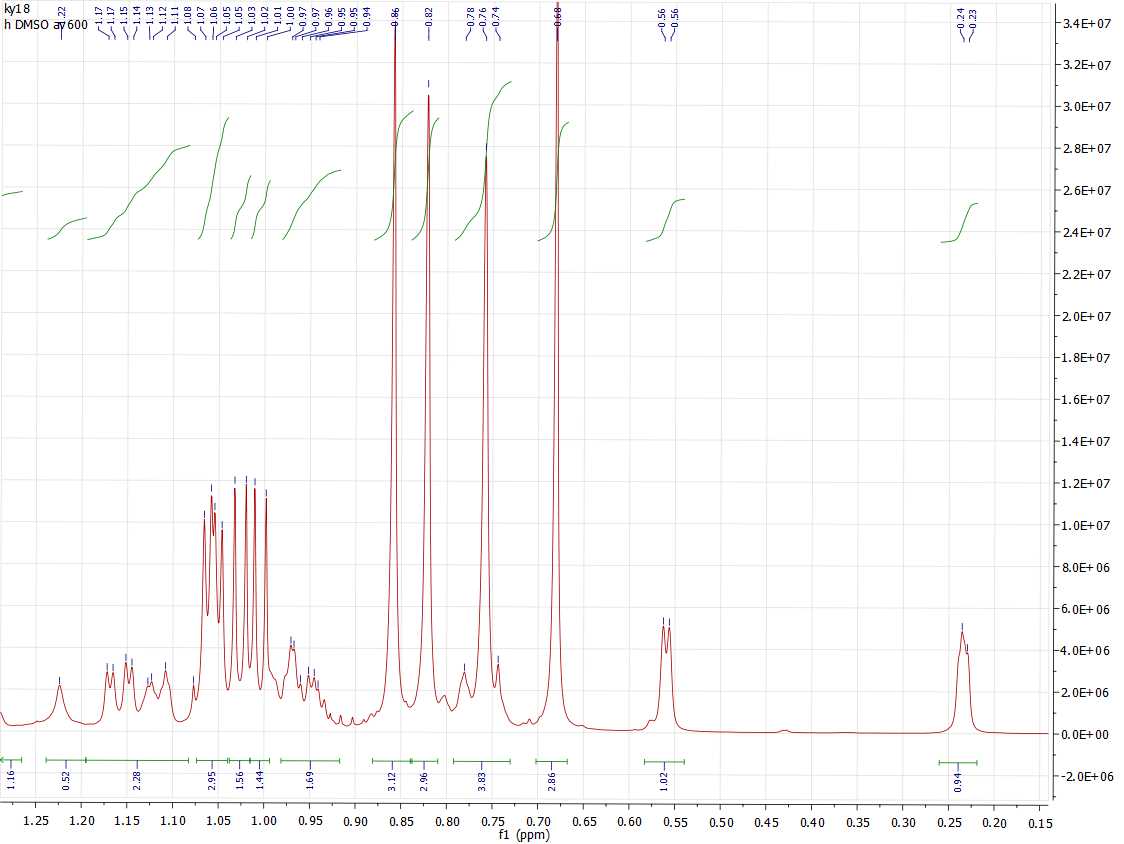


**Figure S26**. ^13^C (150 Hz) NMR Spectrum of cimyunnins **B** and **C** (**2** and **3**) in DMSO-*d*_6_


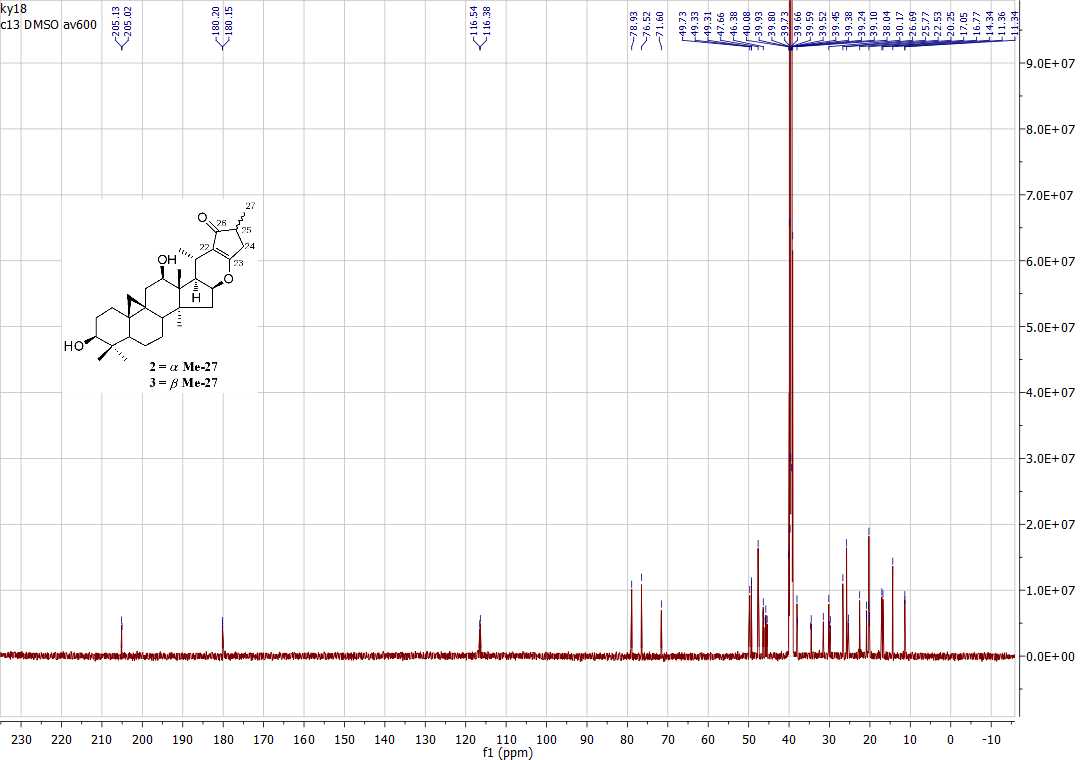


**Figure S27**. Expansions of ^13^C (150 Hz) NMR Spectrum of cimyunnins **B** and **C** (**2** and **3**) in DMSO-*d*_6_


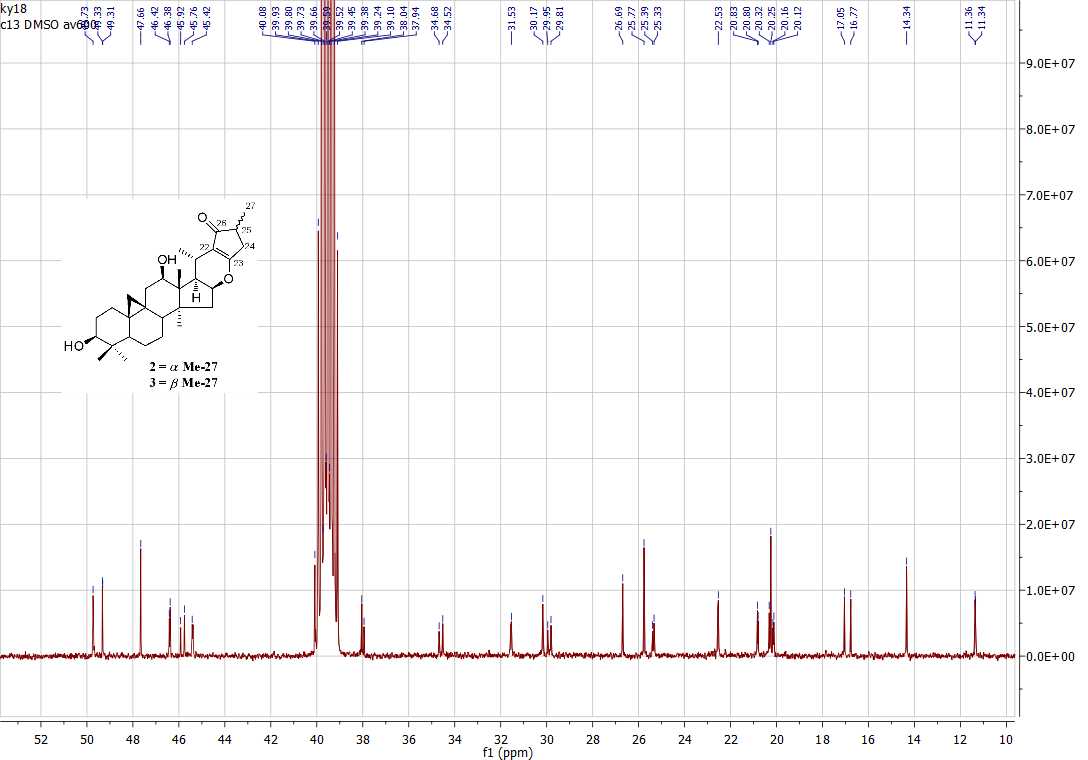


**Figure S28**. Expansions of ^13^C (150 Hz) NMR Spectrum of cimyunnins **B** and **C** (**2** and **3**) in DMSO-*d*_6_


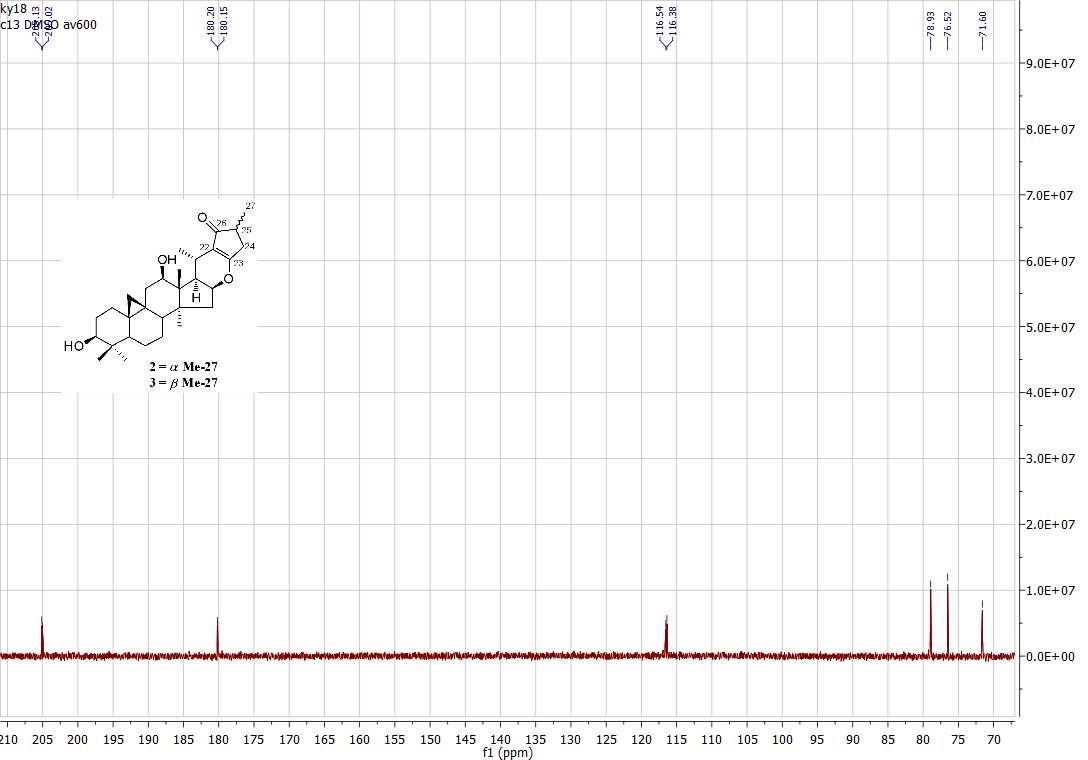


**Figure S29**. DEPT (90°) (150 Hz) NMR Spectrum of cimyunnins **B** and **C** (**2** and **3**) in DMSO-*d*_6_


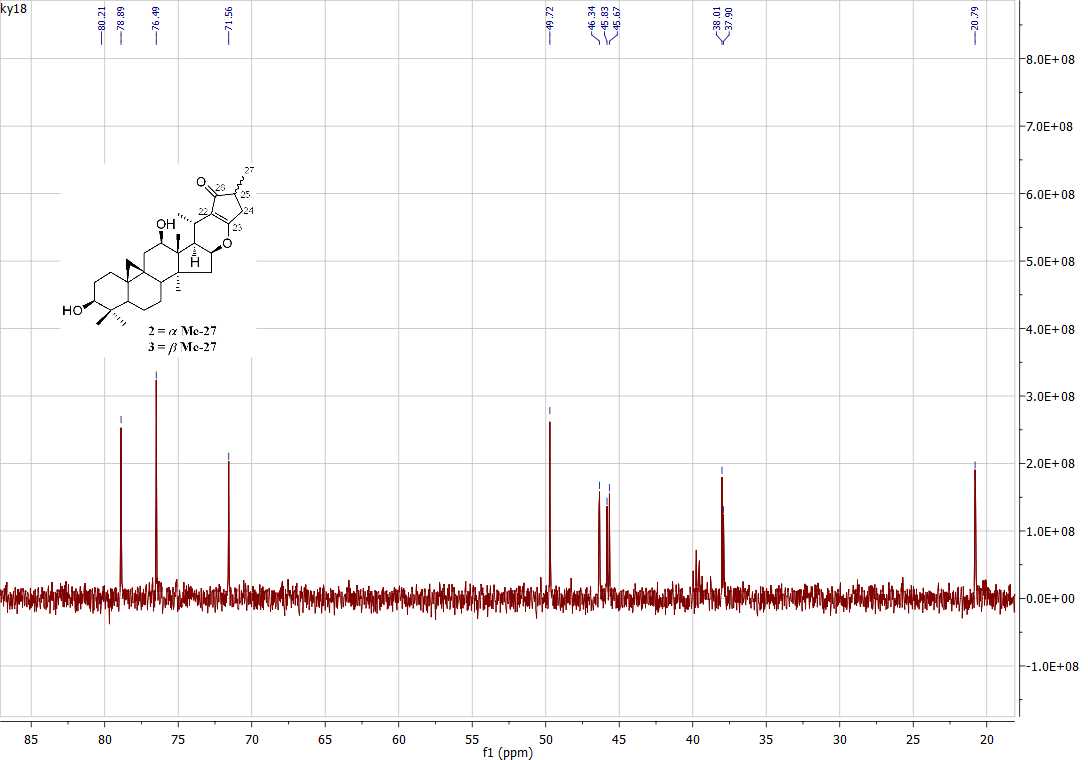


**Figure S30**. EDPT (135°) (150 Hz) NMR Spectrum of cimyunnins **B** and **C** (**2** and **3**) in DMSO-*d*_6_


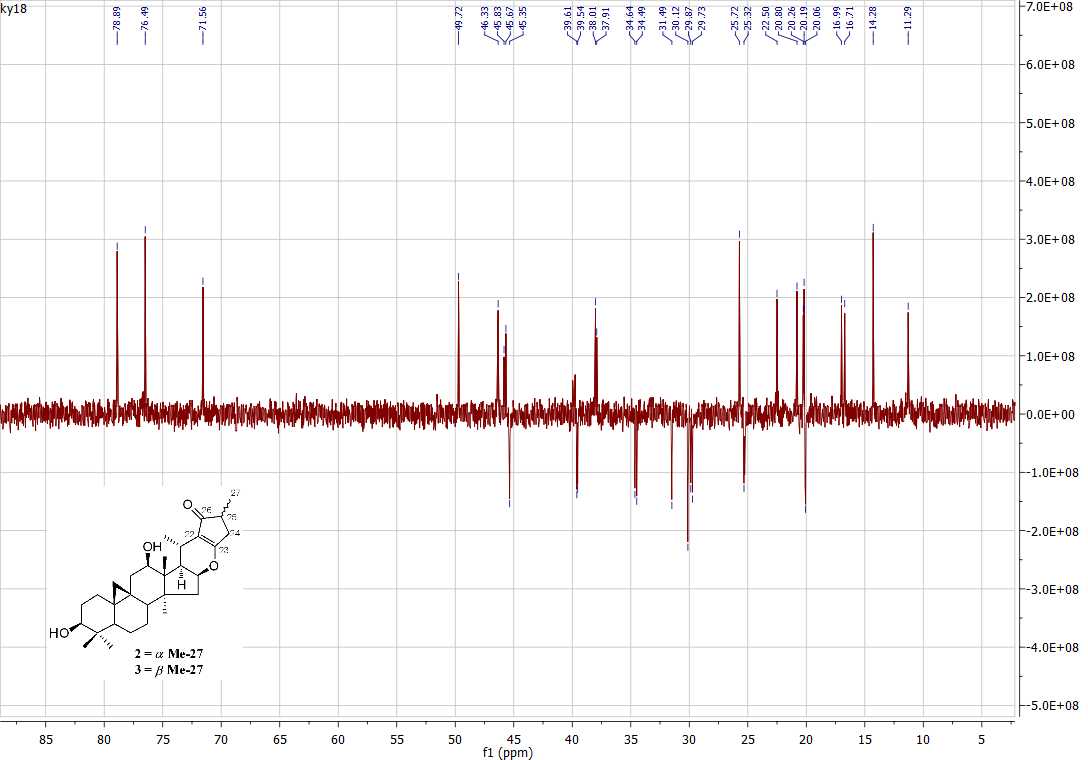


**Figure S31**. HSQC (600 Hz) NMR Spectrum of cimyunnins **B** and **C** (**2** and **3**) in DMSO-*d*_6_


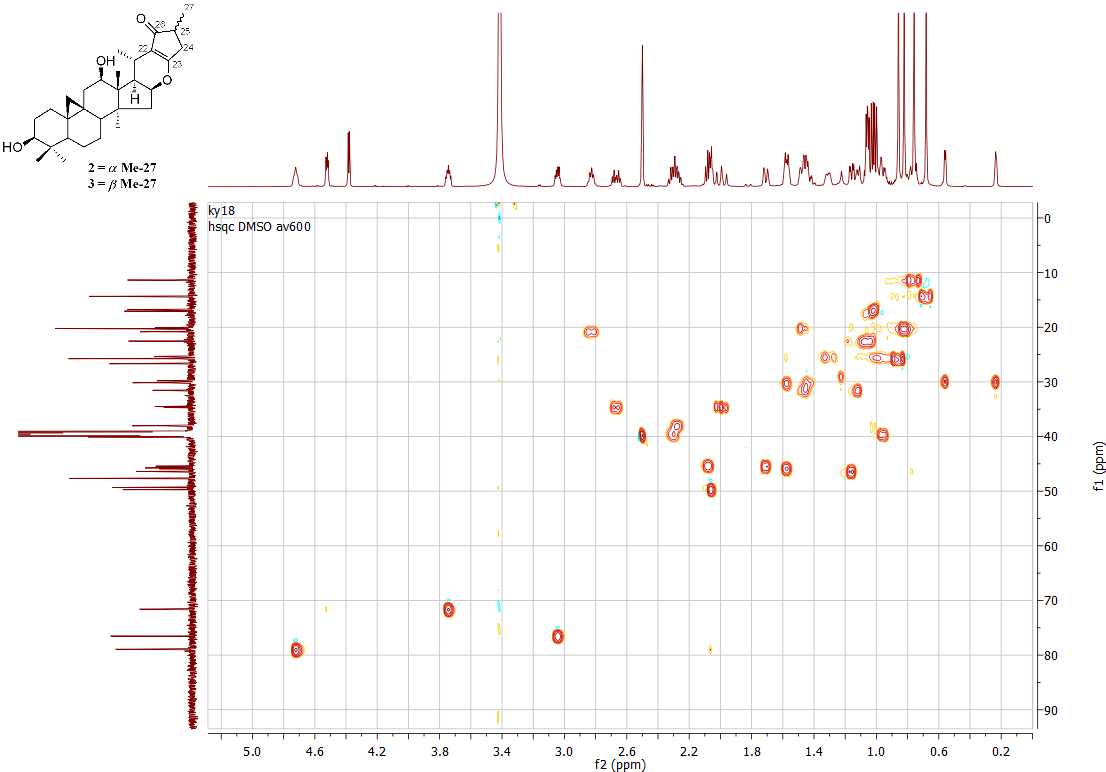


**Figure S32**. Expansions of HSQC (600 Hz) NMR Spectrum of cimyunnins **B** and **C** (**2** and **3**) in DMSO-*d*_6_


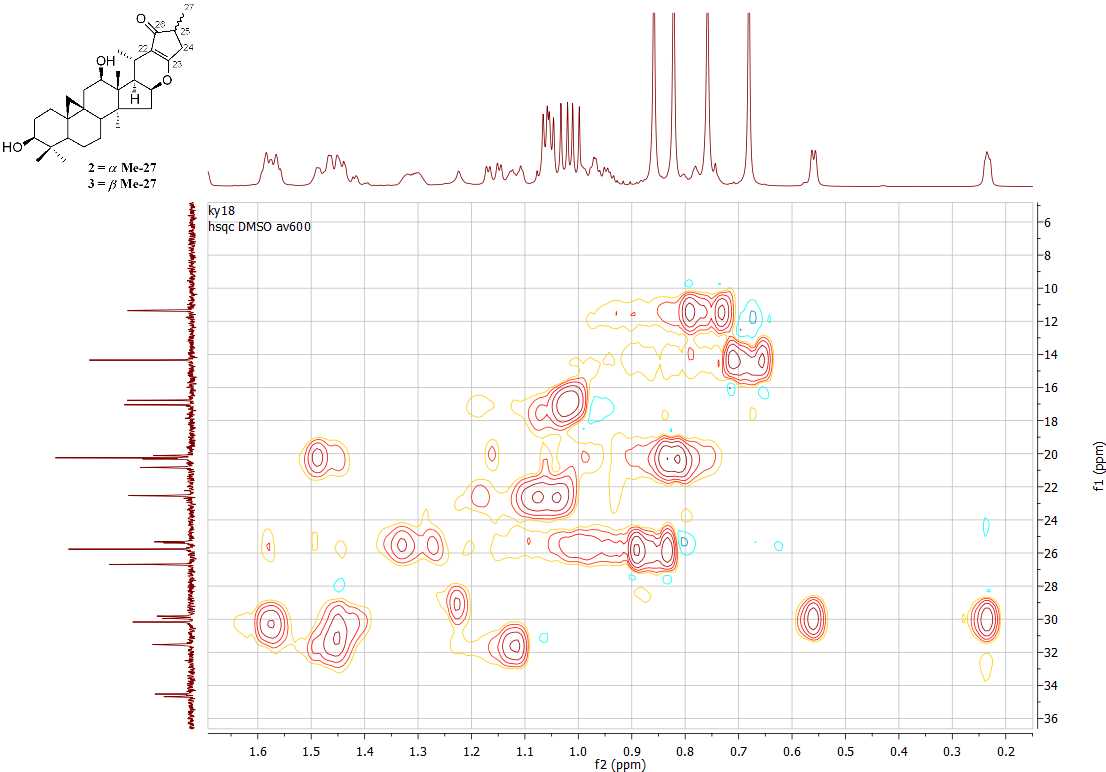


**Figure S33**. HMBC (600 Hz) NMR Spectrum of cimyunnins **B** and **C** (**2** and **3**) in DMSO-*d*_6_


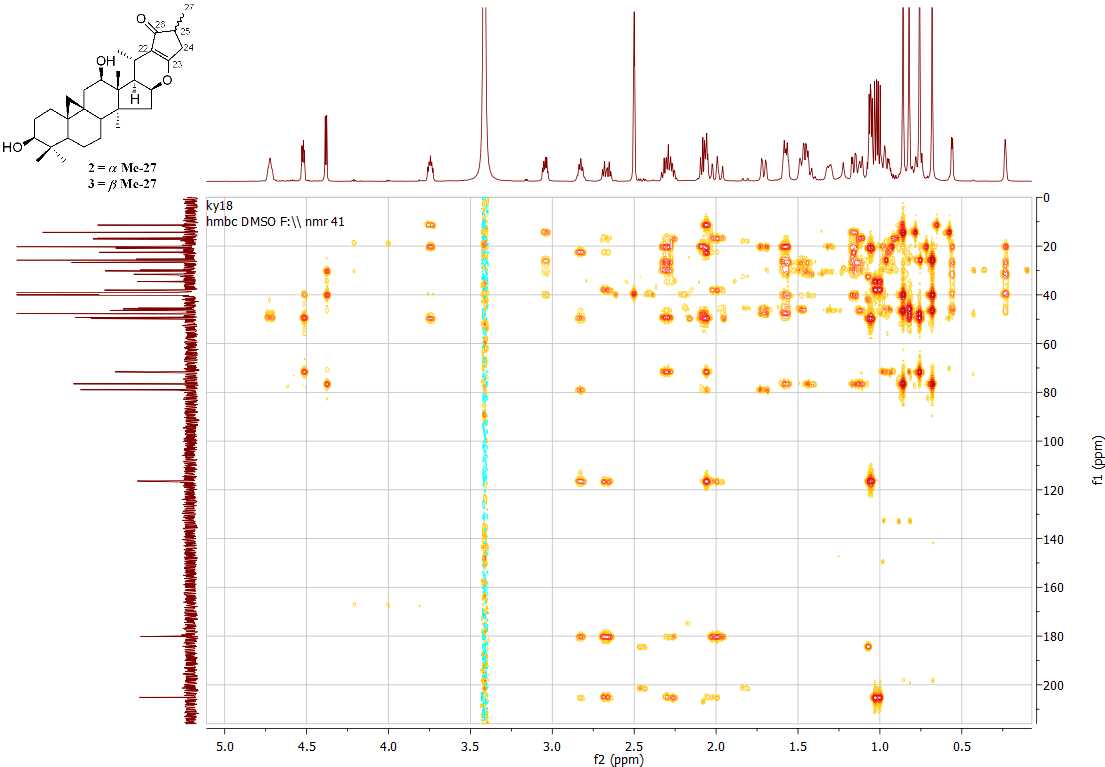


**Figure S34**. Expansions of HMBC (600 Hz) NMR Spectrum of cimyunnins **B** and **C** (**2** and **3**) in DMSO-*d*_6_


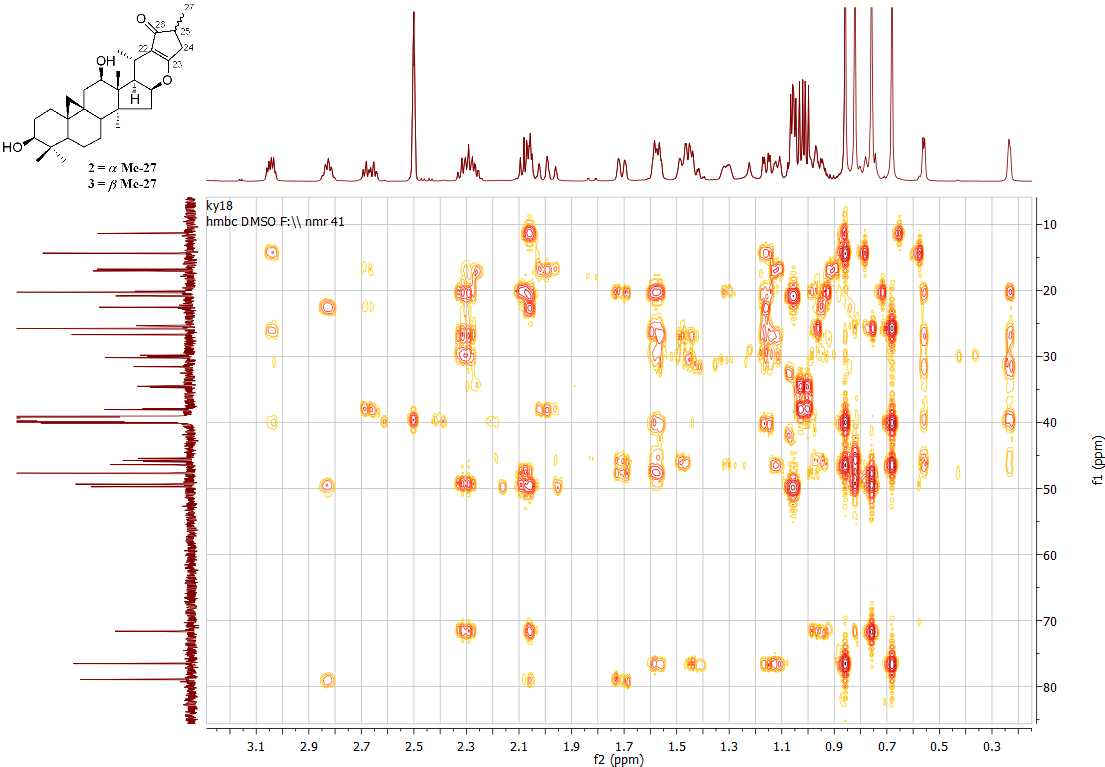


**Figure S35**. Expansions of HMBC (600 Hz) NMR Spectrum of cimyunnins **B** and **C** (**2** and **3**) in DMSO-*d*_6_


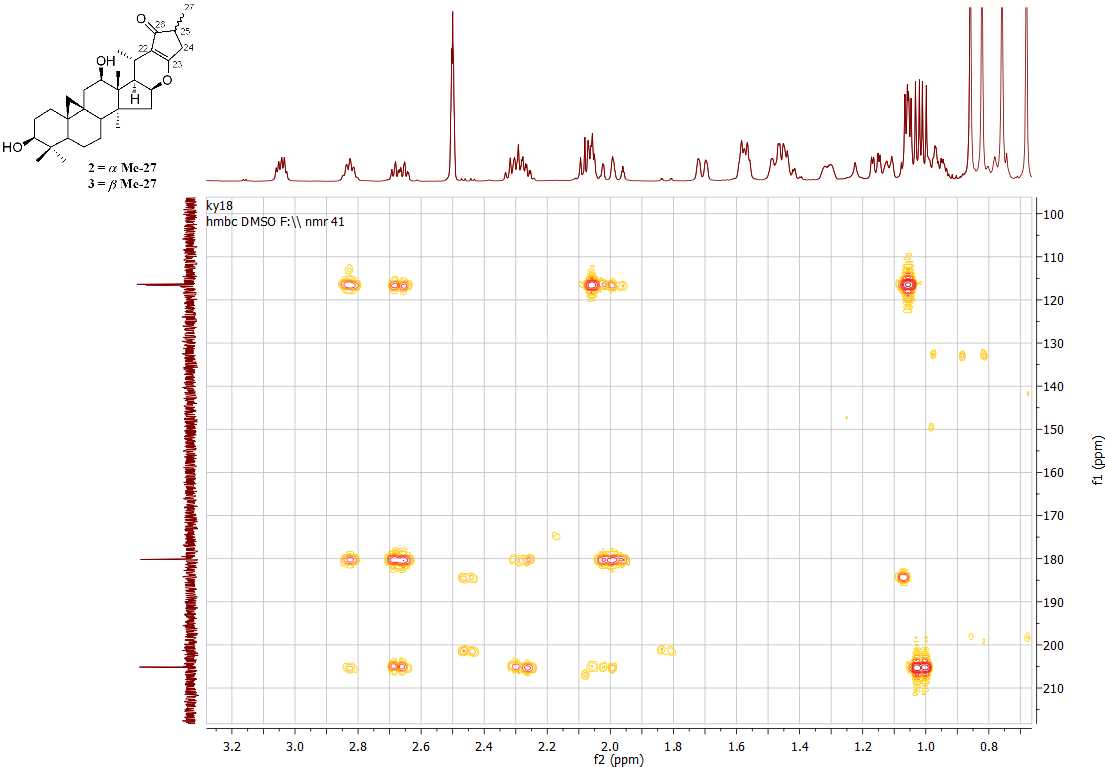


**Figure S36**. Expansions of HMBC (600 Hz) NMR Spectrum of cimyunnins **B** and **C** (**2** and **3**) in DMSO-*d*_6_


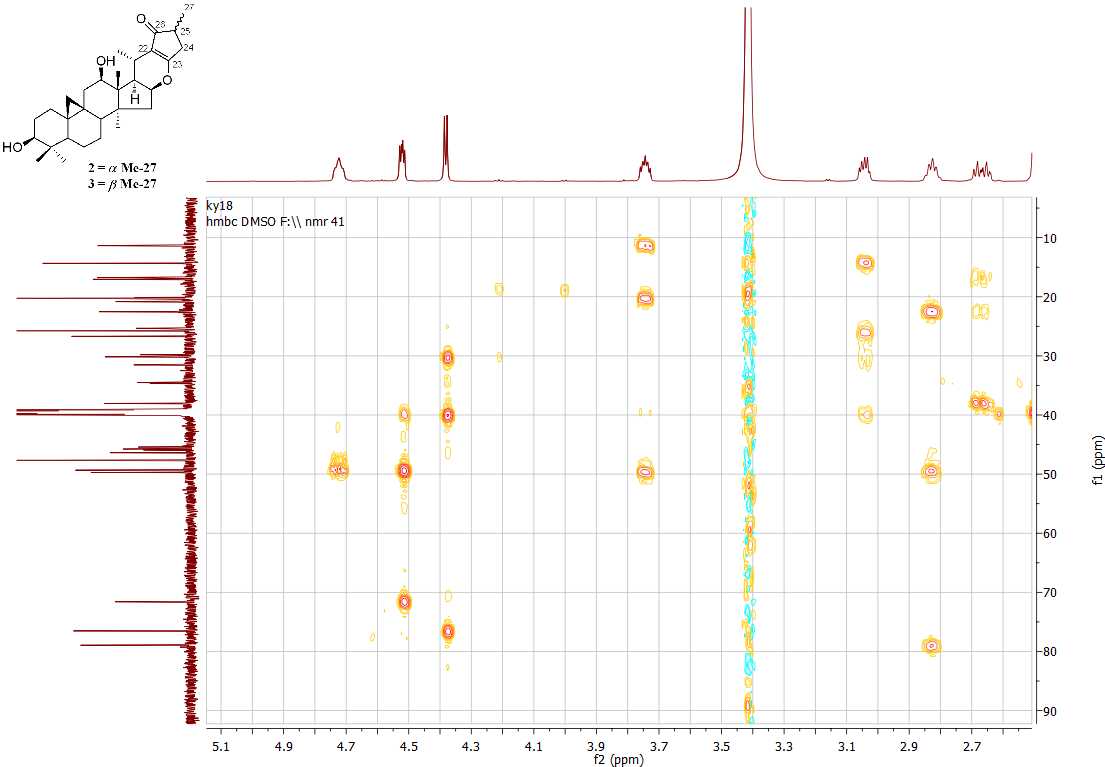


**Figure S37**. ^1^H-^1^H COSY (600 Hz) NMR Spectrum of cimyunnins **B** and **C** (**2** and **3**) in DMSO-*d*_6_


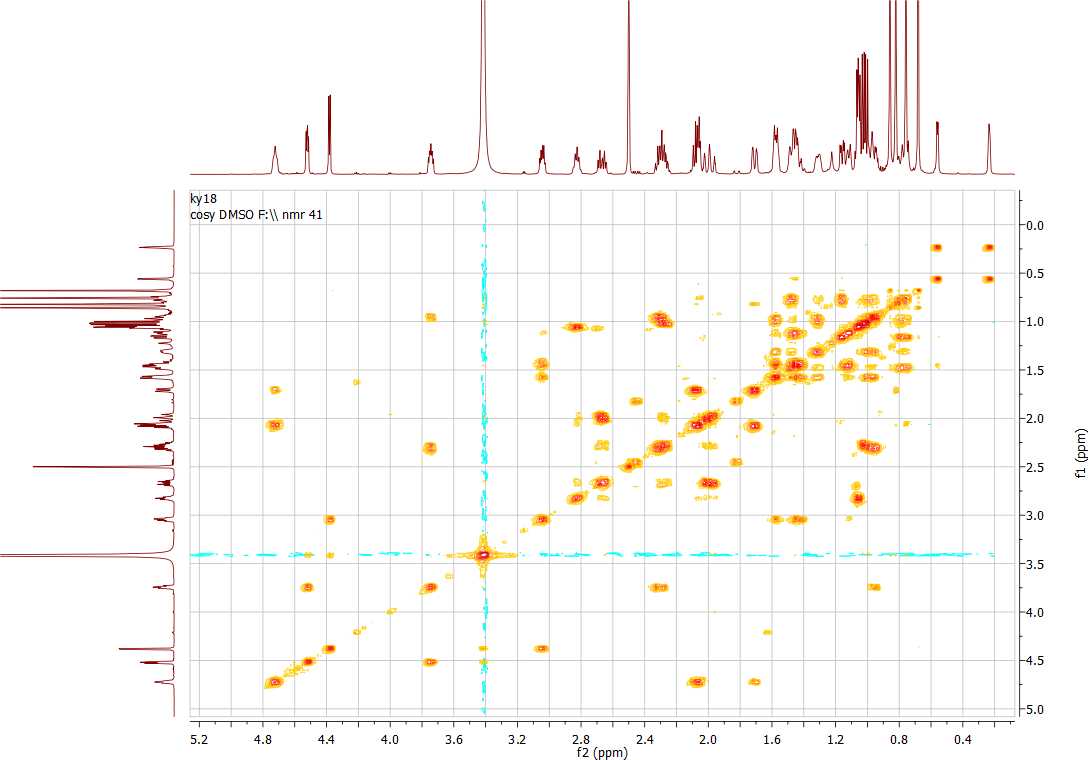


**Figure S38.** Expansions of ^1^H-^1^H COSY (600 Hz) NMR Spectrum of cimyunnins **B** and **C** (**2** and **3**) in DMSO-*d*_6_


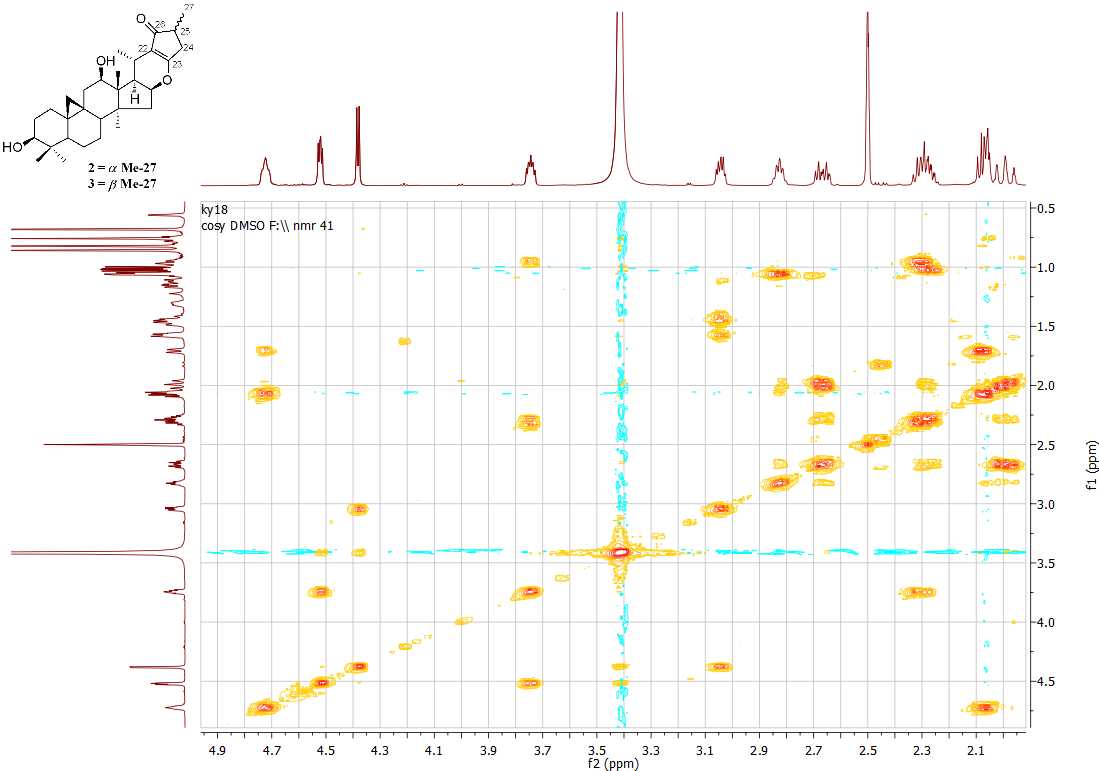


**Figure S39**. Expansions of ^1^H-^1^H COSY (600 Hz) NMR Spectrum of cimyunnins **B** and **C** (**2** and **3**) in DMSO-*d*_6_


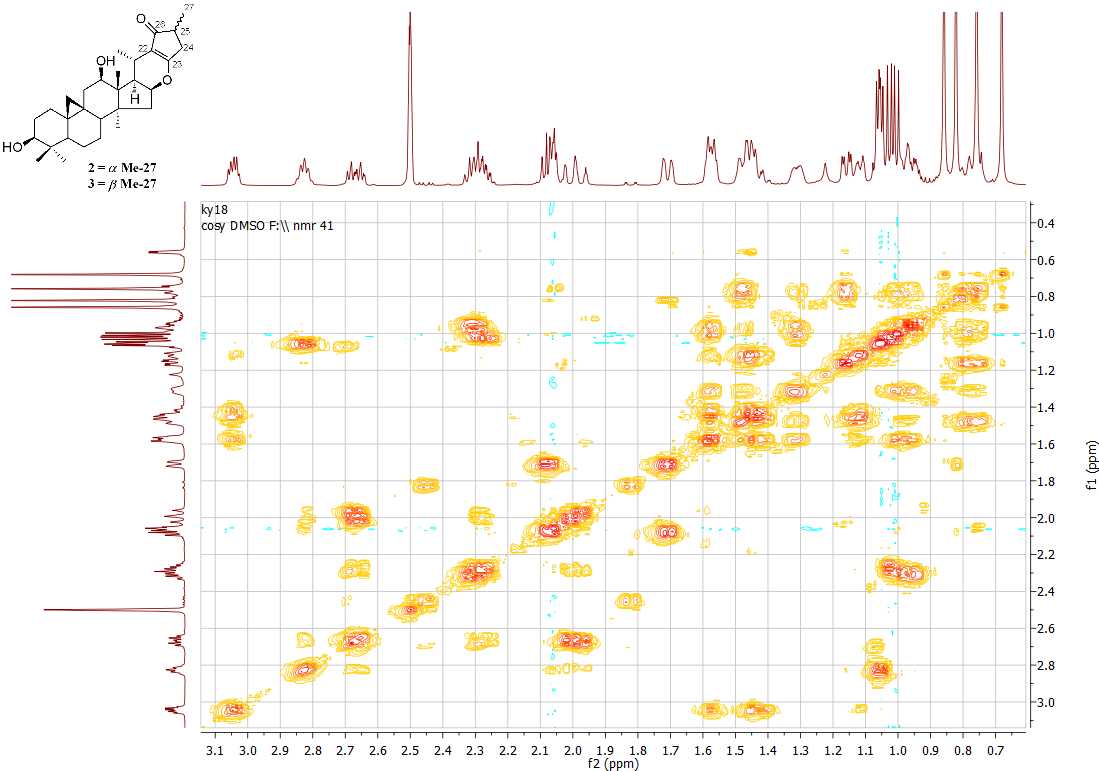


**Figure S40**. ROESY (600 Hz) NMR Spectrum of cimyunnins **B** and **C** (**2** and **3**) in DMSO-*d*_6_


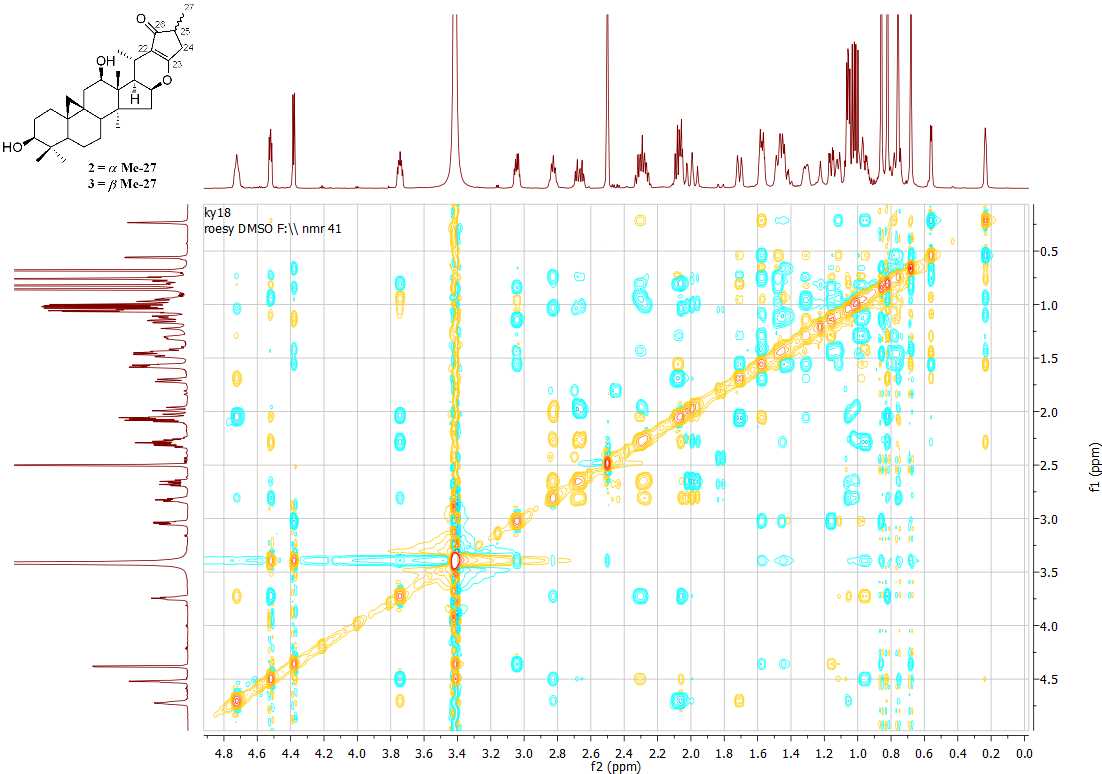


**Figure S41**. Expansions of ROESY (600 Hz) NMR Spectrum of cimyunnins **B** and **C** (**2** and **3**) in DMSO-*d*_6_


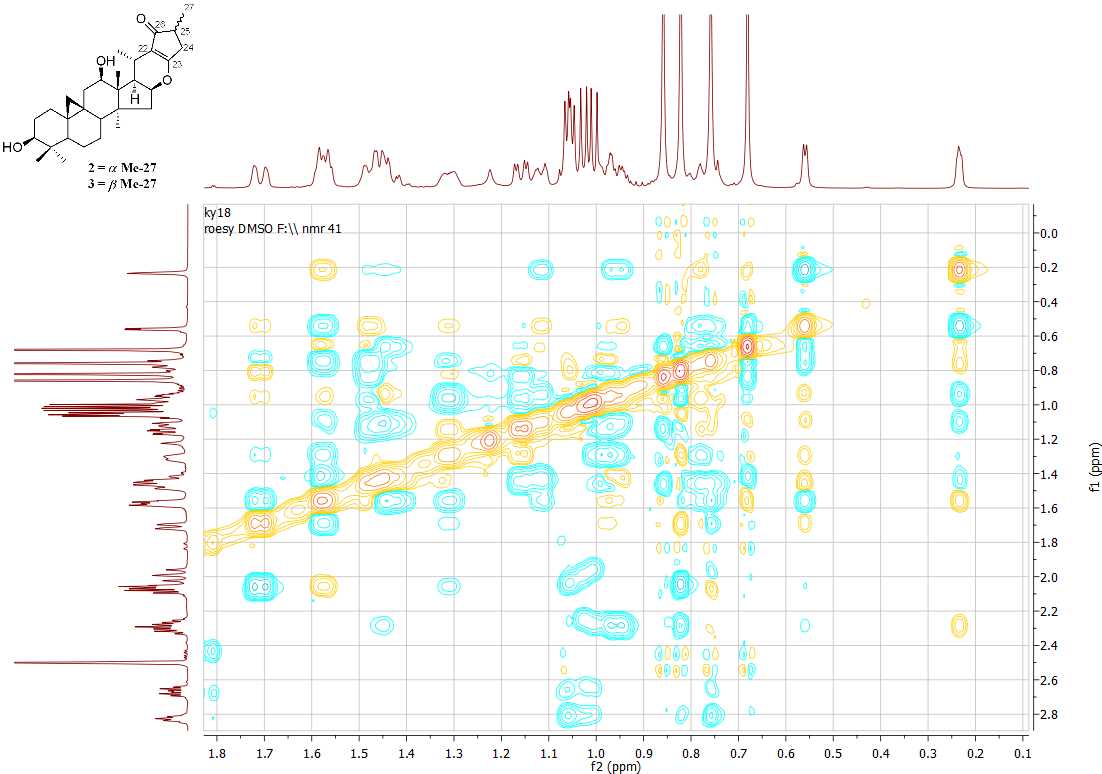


**Figure S42**. Expansions of ROESY (600 Hz) NMR Spectrum of cimyunnins **B** and **C** (**2** and **3**) in DMSO-*d*_6_


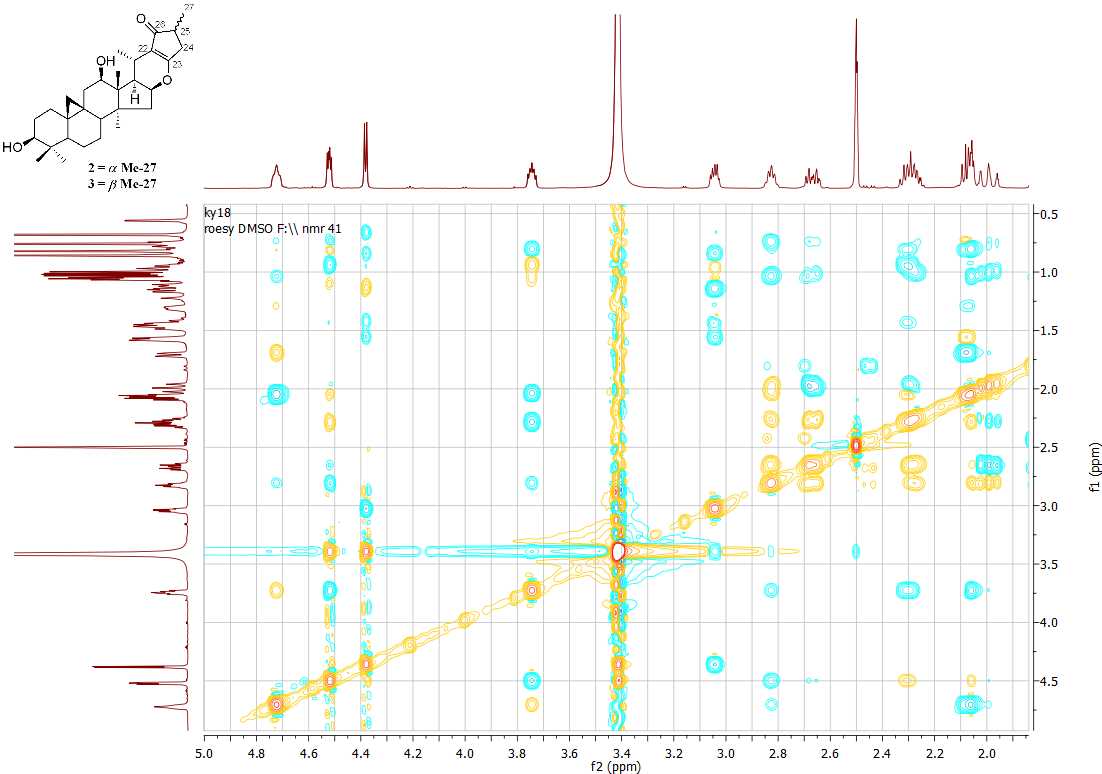


**Figure S43**. ^1^H (600 Hz) NMR Spectrum of cimyunnins **B** and **C** (**2** and **3**) in Pyridine-*d*_5_


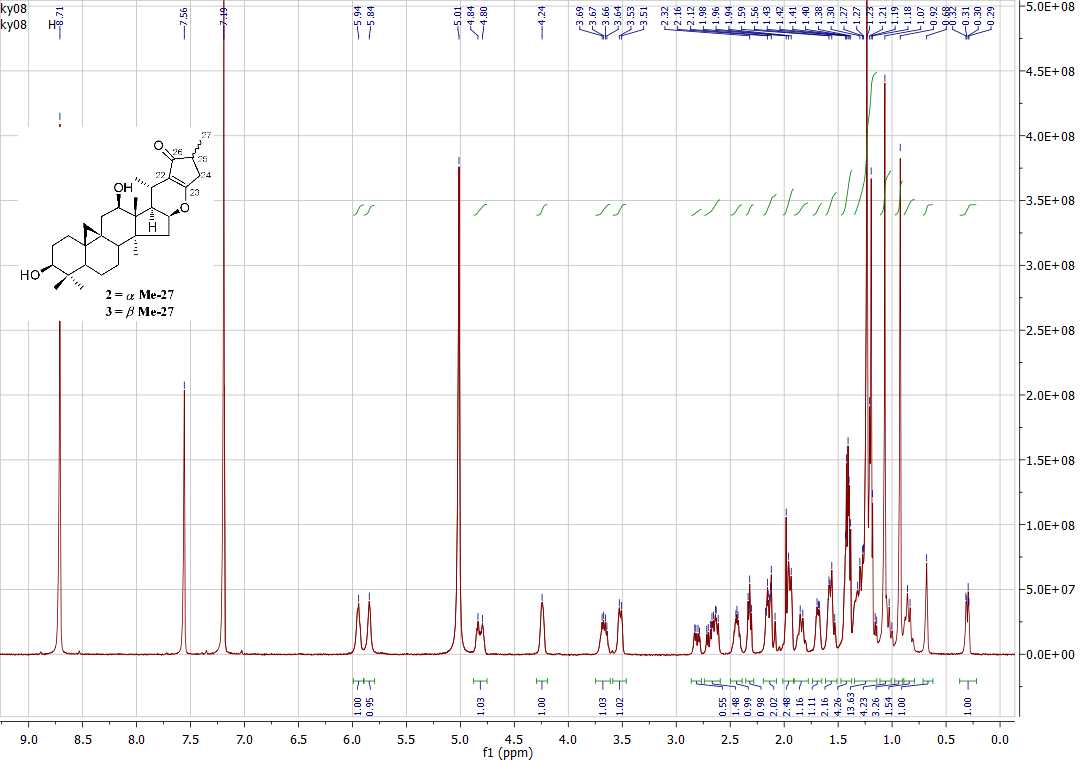


**Figure S44.** Expansions of 1H (600 Hz) NMR Spectrum of cimyunnins **B** and **C** (**2** and **3**) in Pyridine-d5


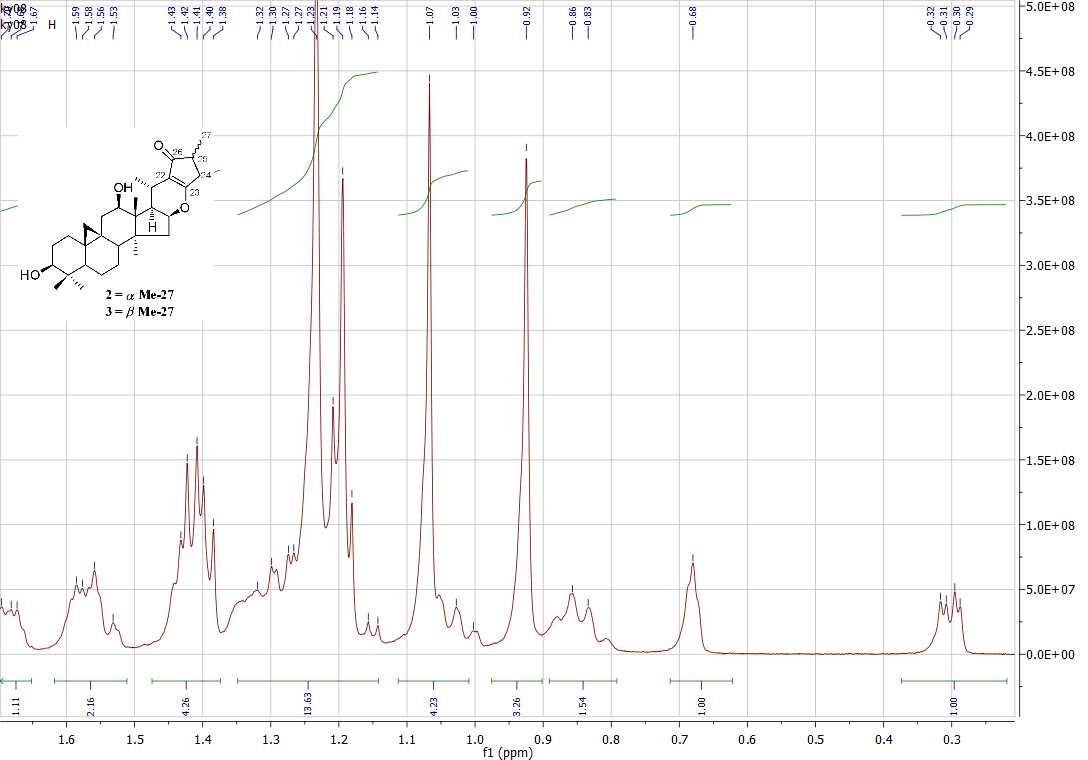


**Figure S45**. ^13^C (150 Hz) NMR Spectrum of cimyunnins **B** and **C** (**2** and **3**) in Pyridine-*d*_5_


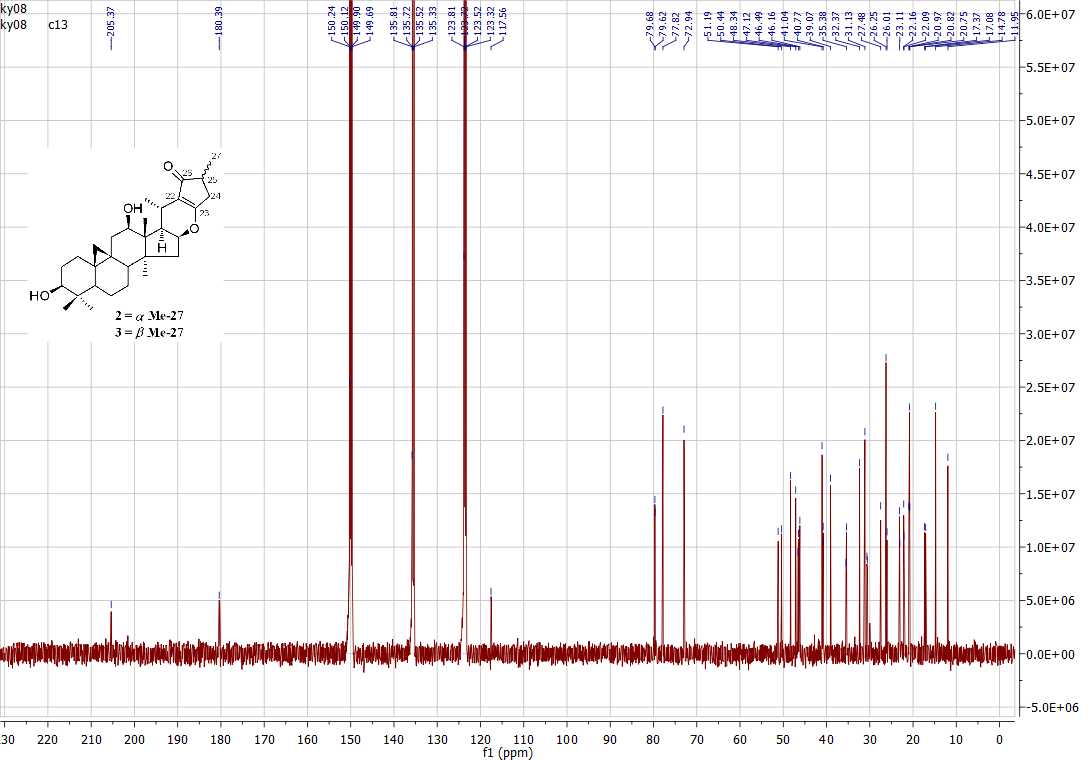


**Figure S46**. Expansions of ^13^C (150 Hz) NMR Spectrum of cimyunnins **B** and **C** (**2** and **3**) in Pyridine-*d*_5_


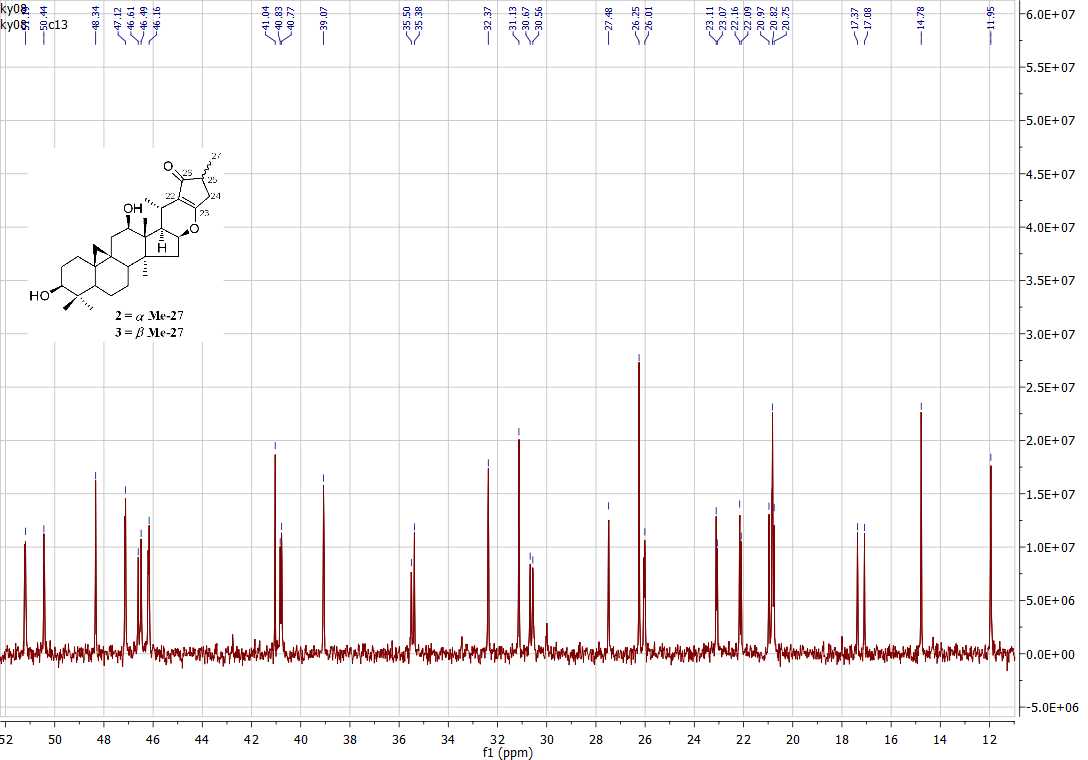


**Figure S47**. Expansions of ^13^C (150 Hz) NMR Spectrum of cimyunnins **B** and **C** (**2** and **3**) in Pyridine-*d*_5_


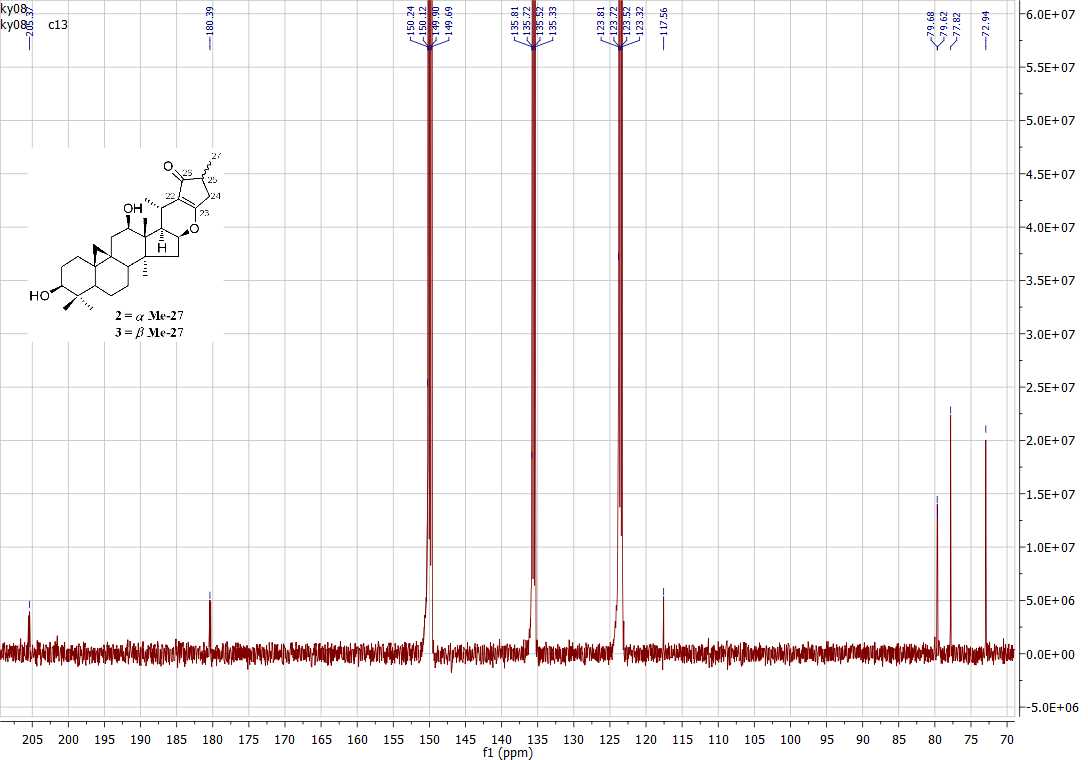


**Figure S48**. HSQC (600 Hz) NMR Spectrum of cimyunnins **B** and **C** (**2** and **3**) in Pyridine-*d*_5_


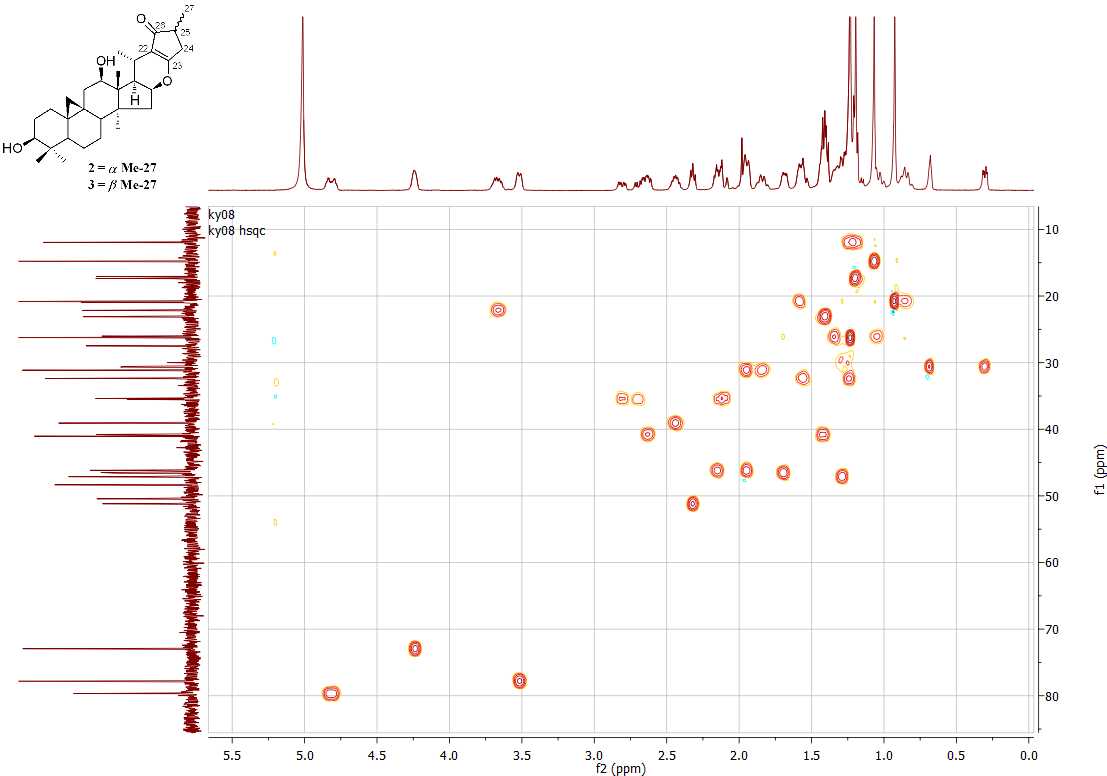


**Figure S49**. HMBC (600 Hz) NMR Spectrum of cimyunnins **B** and **C** (**2** and **3**) in Pyridine-*d*_5_


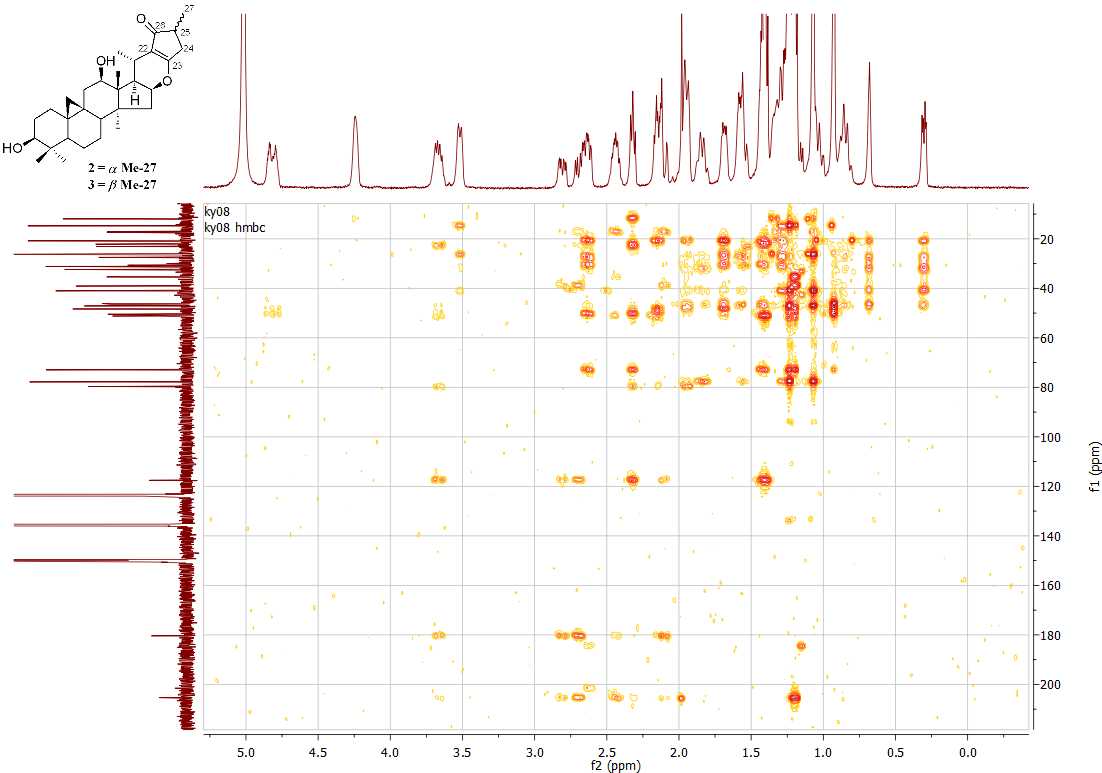


**Figure S50**. ^1^H-^1^H COSY (600 Hz) NMR Spectrum of cimyunnins **B** and **C** (**2** and **3**) in Pyridine-*d*_5_


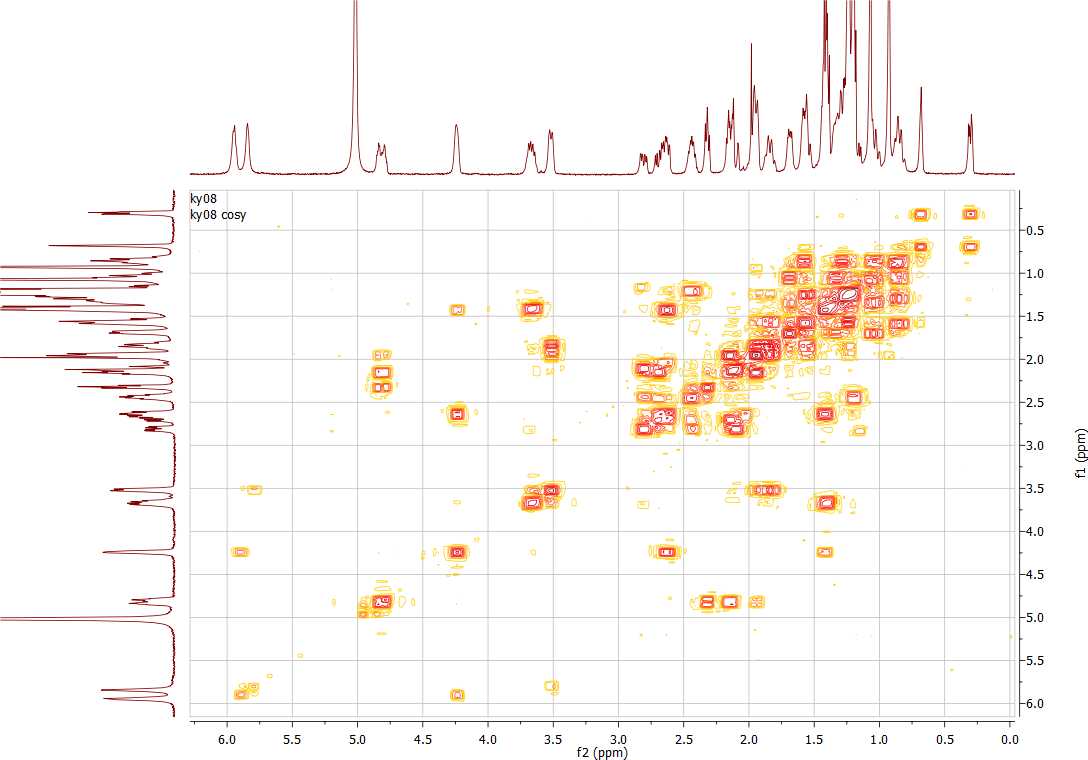


**Figure S51**. ROESY (600 Hz) NMR Spectrum of cimyunnins **B** and **C** (**2** and **3**) in Pyridine-*d*_5_


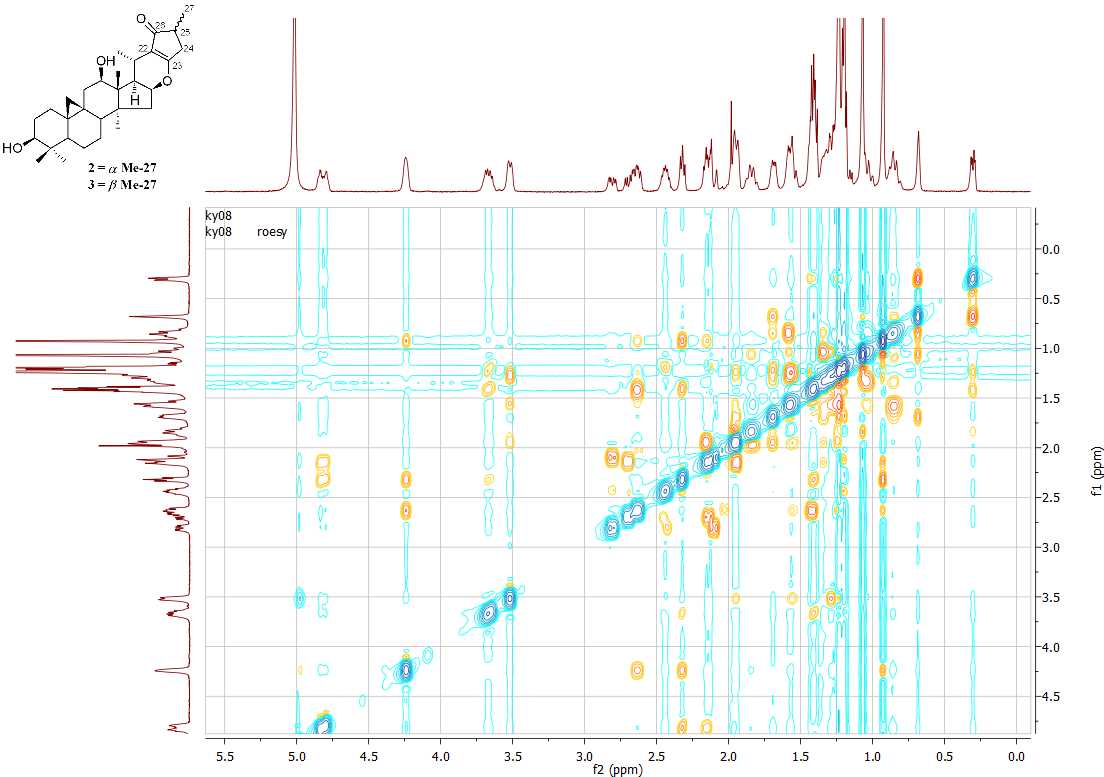


**Figure S52**. HREIMS of cimyunnins **B** and **C** (**2** and **3**)


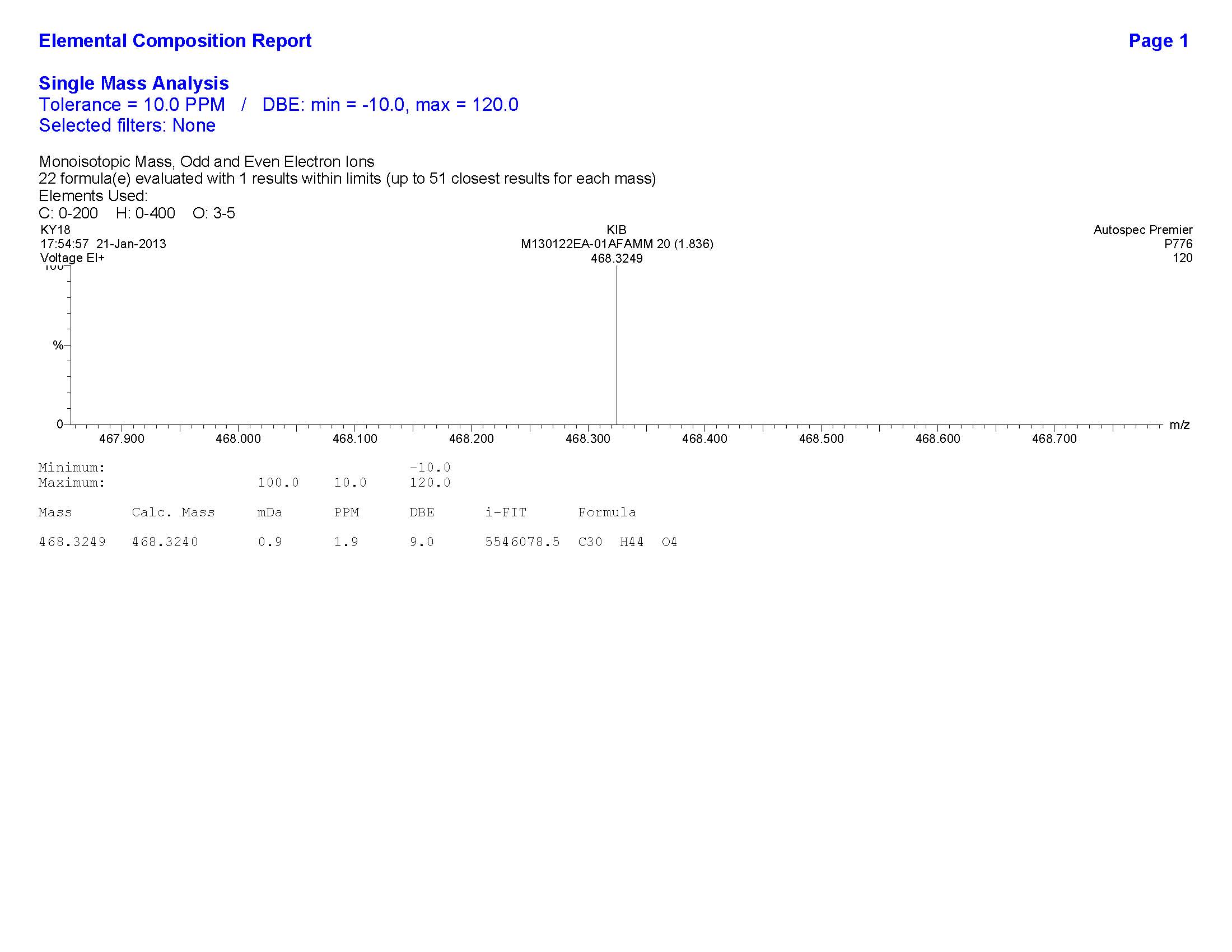


**Figure S53**. IR of cimyunnins **B** and **C** (**2** and **3**)

**Figure S54**. CD and UV of cimyunnins **B** and **C** (**2** and **3**)

##
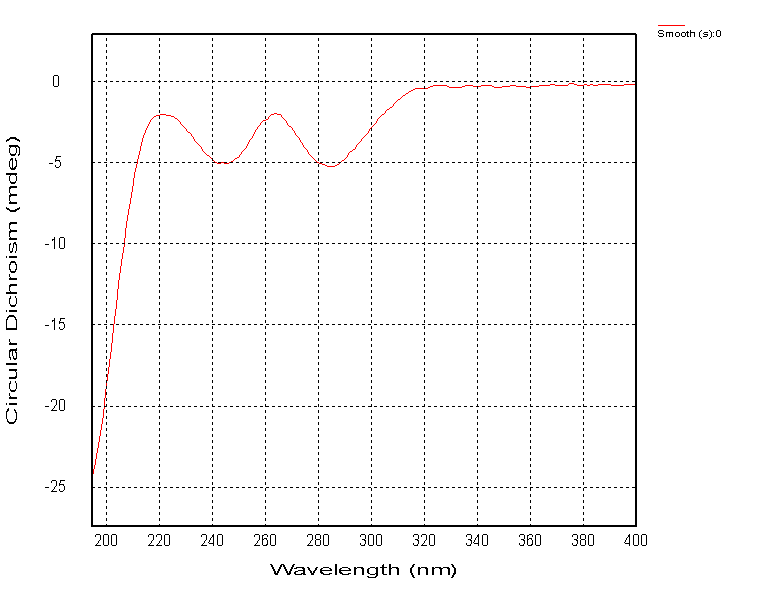

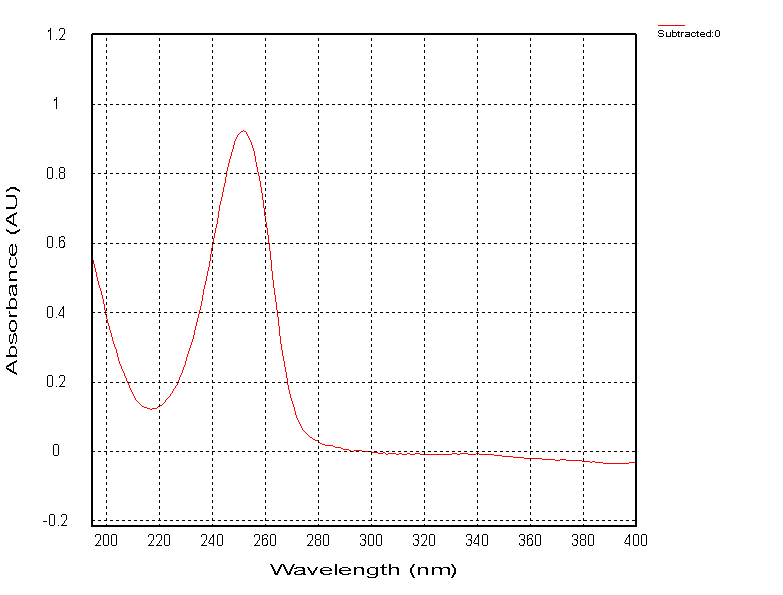


**Figure S55.** ^1^H (600 Hz) NMR Spectrum of cimyunnin **D** (**4**)


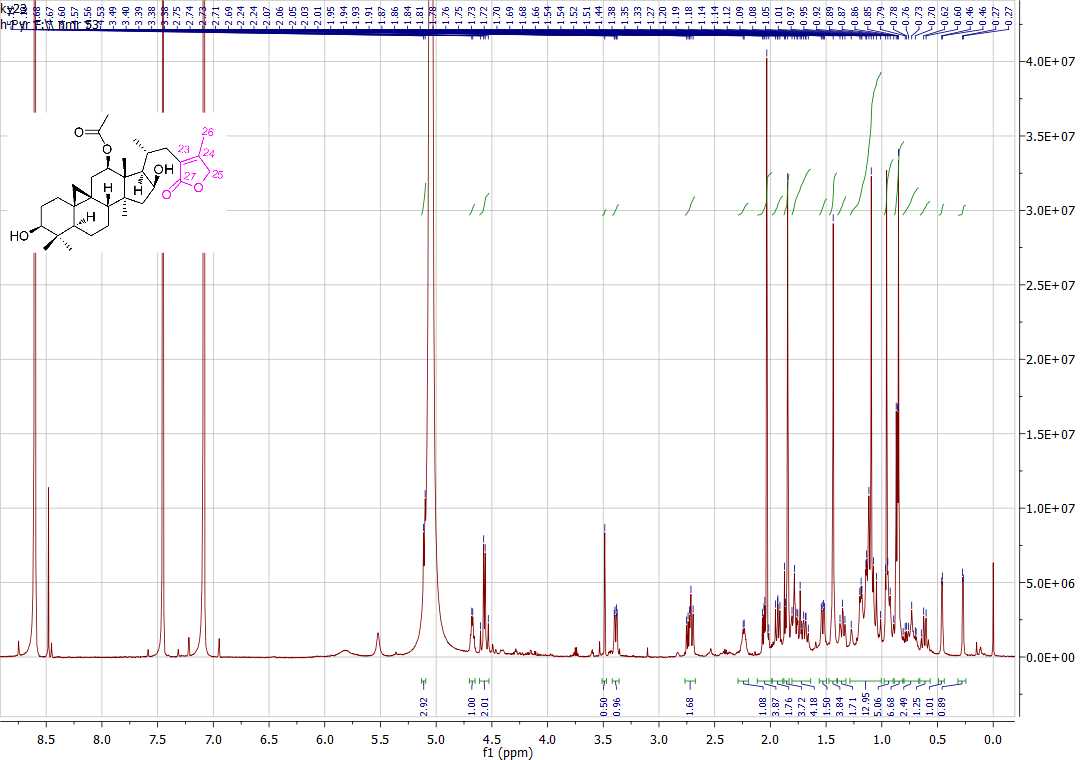


**Figure S56**. Expansions of ^1^H (600 Hz) NMR Spectrum of cimyunnin **D** (**4**)


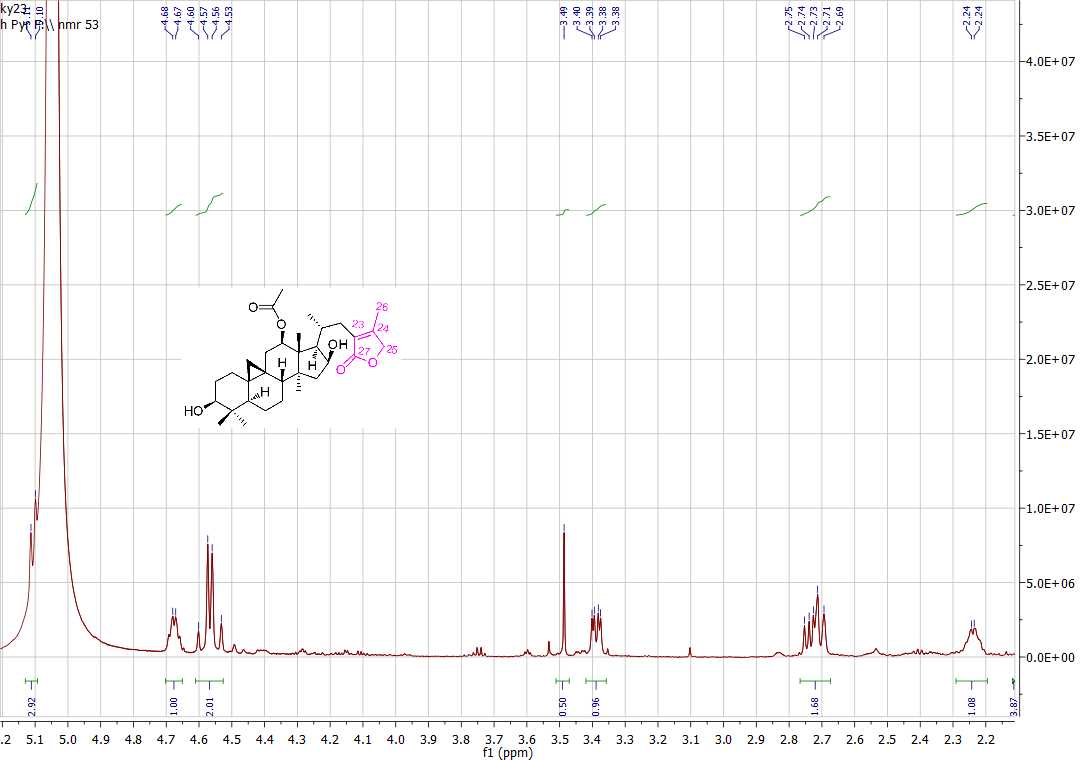


**Figure S57.** Expansions of ^1^H (600 Hz) NMR Spectrum of cimyunnin **D** (**4**)


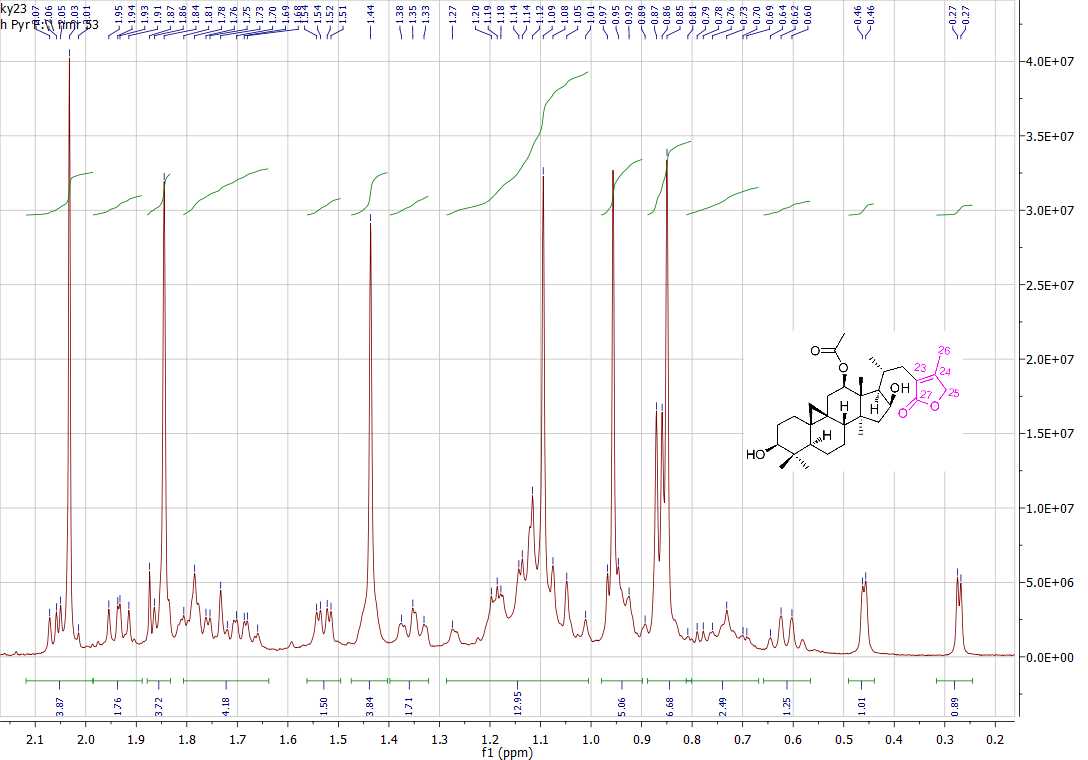


**Figure S58**. ^13^C (150 Hz) NMR Spectrum of cimyunnin **D** (**4**)


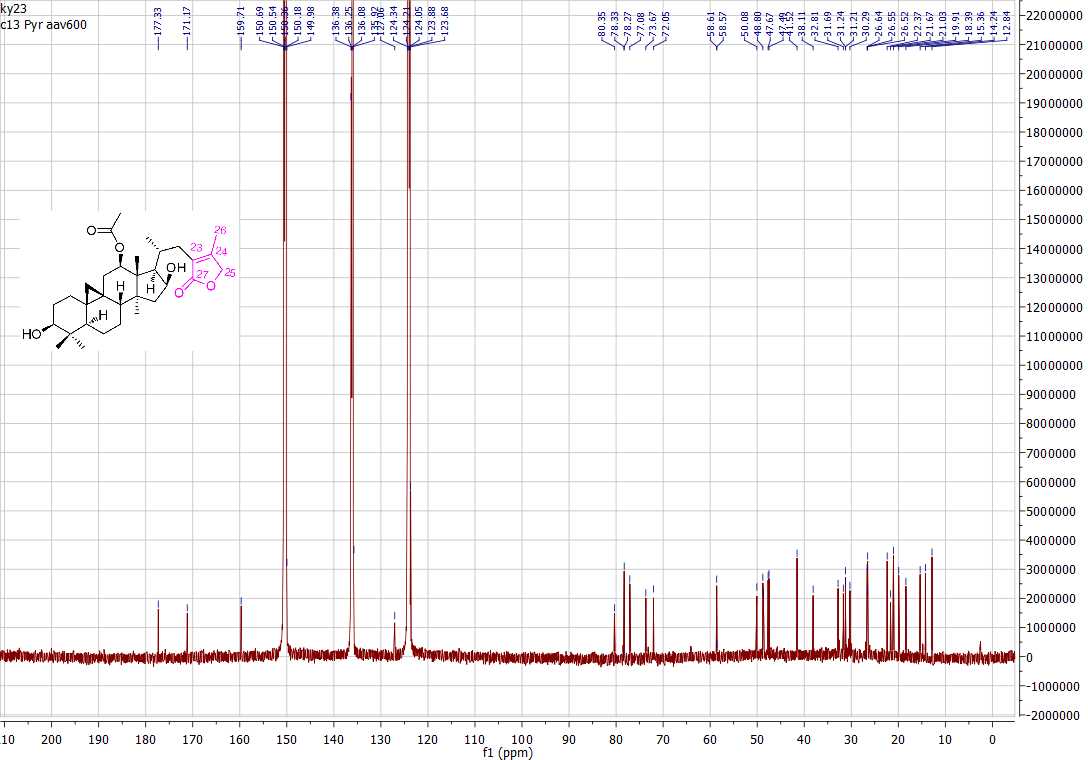


**Figure S59**. Expansions of ^13^C (150 Hz) NMR Spectrum of cimyunnin **D** (**4**)


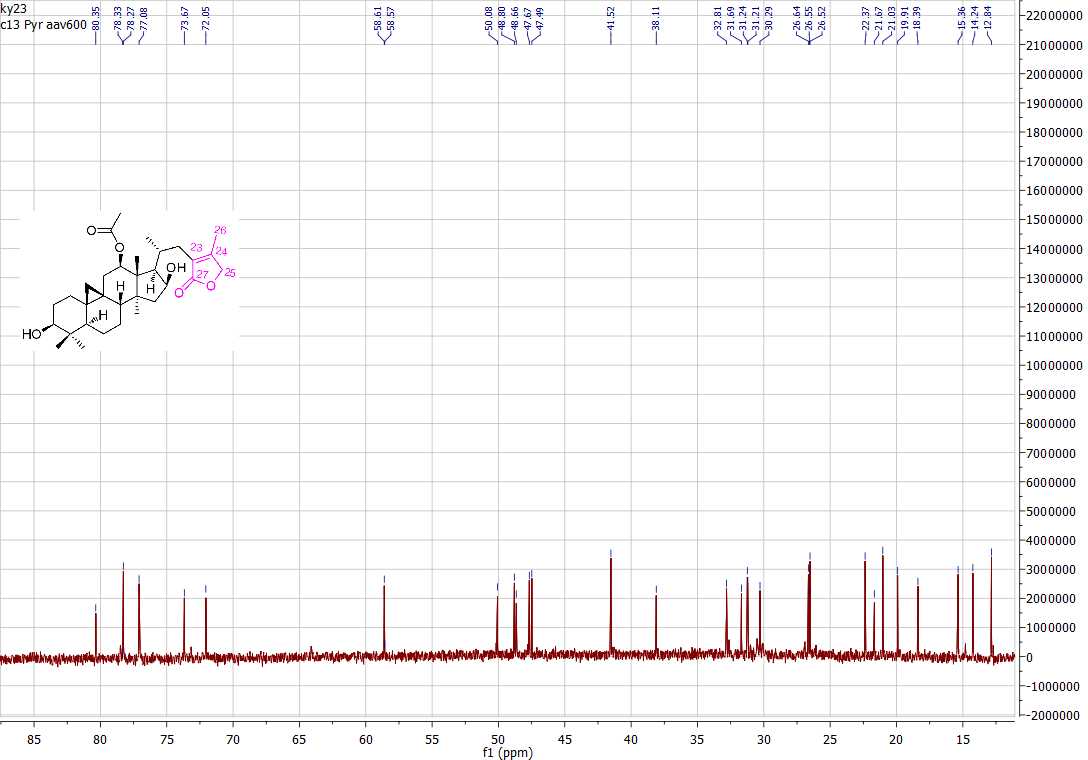


**Figure S60.** HSQC (600 Hz) Spectrum of cimyunnin **D** (**4**)


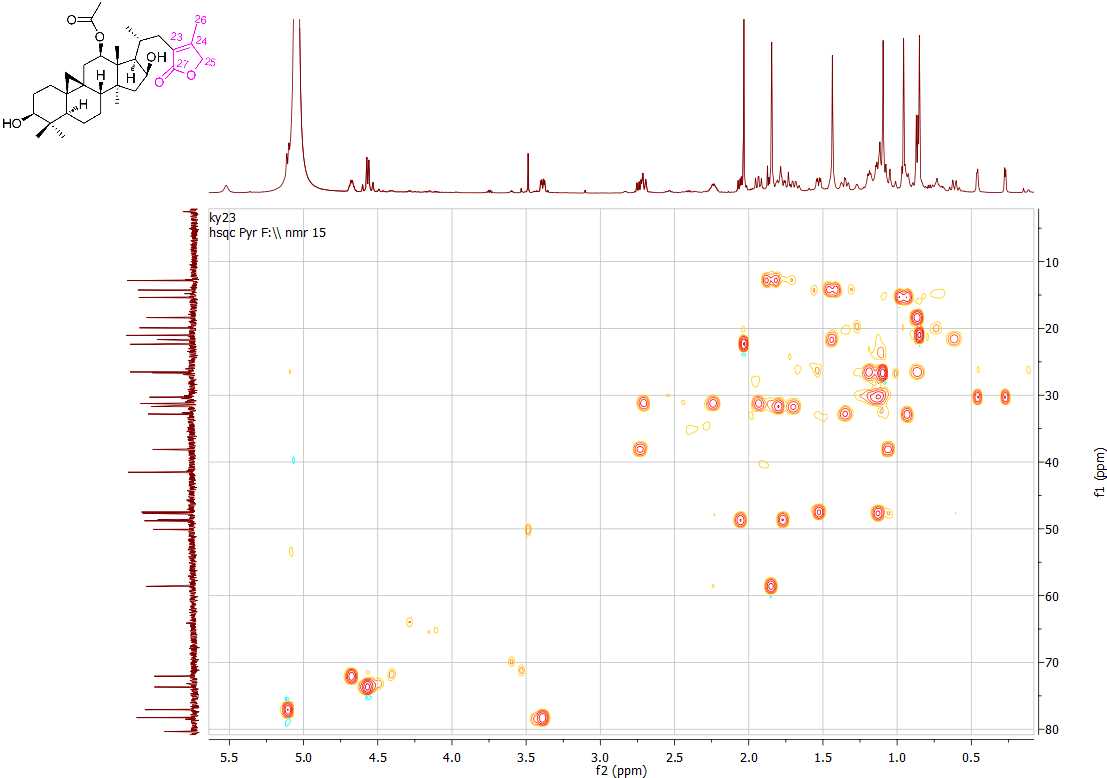


**Figure S61.** HMBC (600 Hz) Spectrum of cimyunnin **D** (**4**)


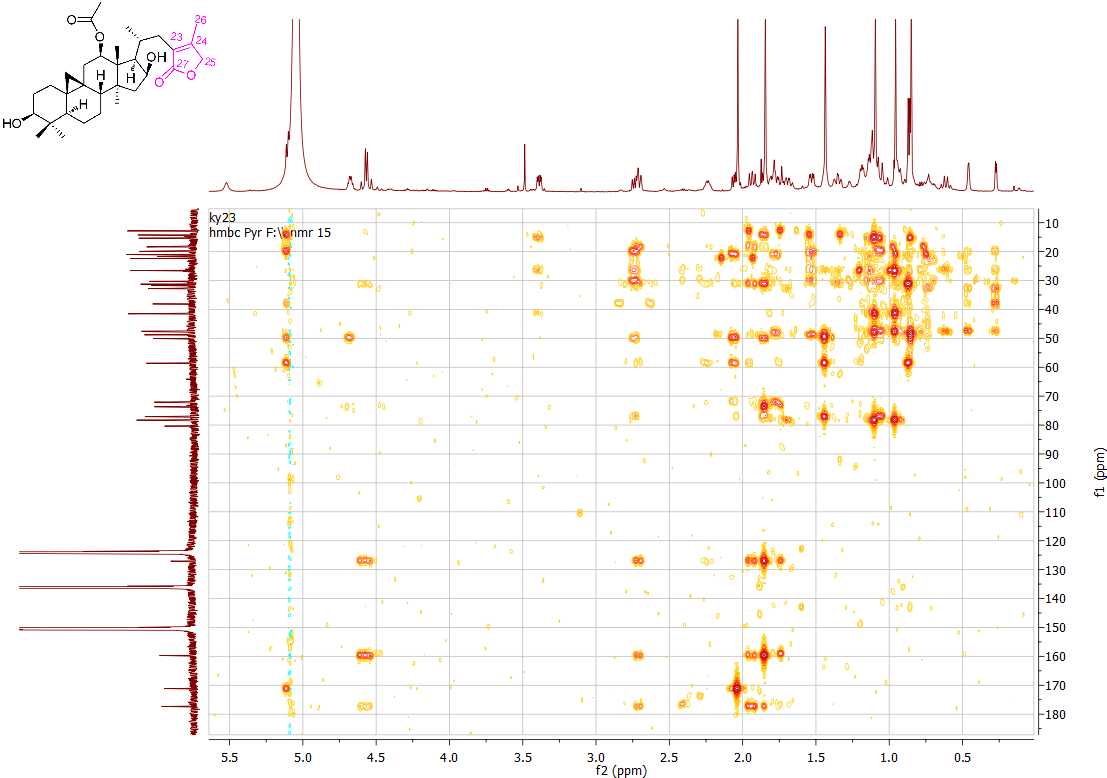


**Figure S62.** Expansions of HMBC (600 Hz) Spectrum of cimyunnin **D** (**4**)


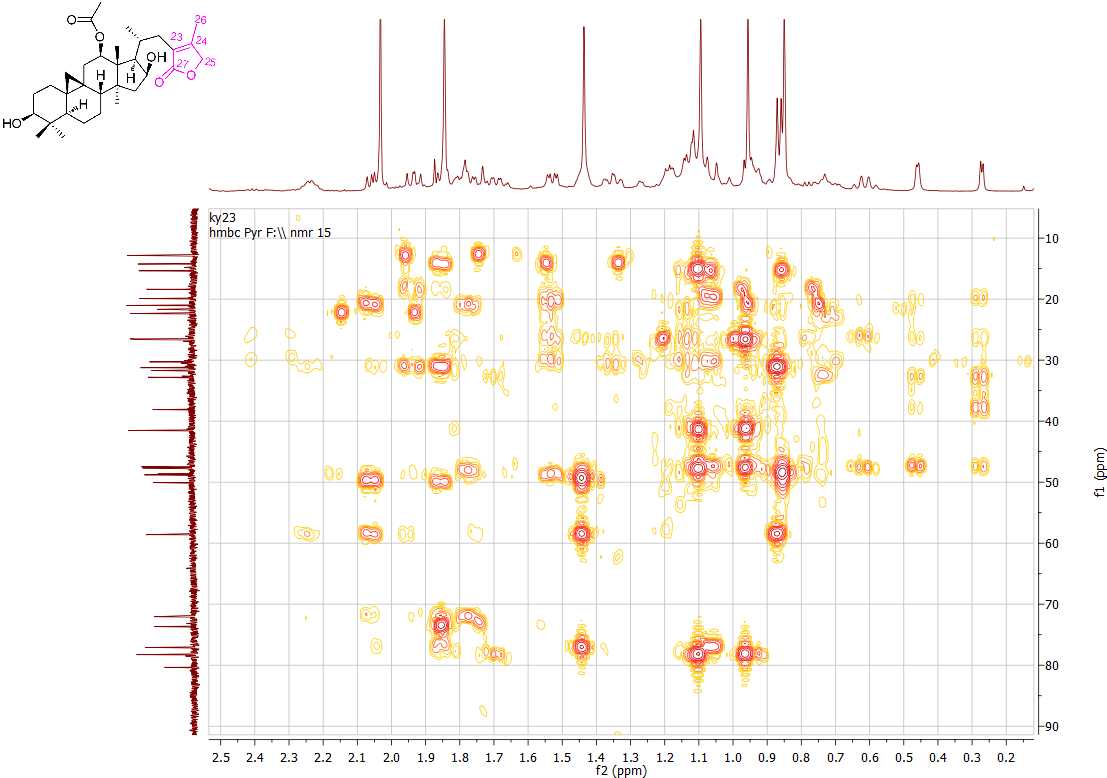


**Figure S63**. Expansions of HMBC (600 Hz) Spectrum of cimyunnin **D** (**4**)


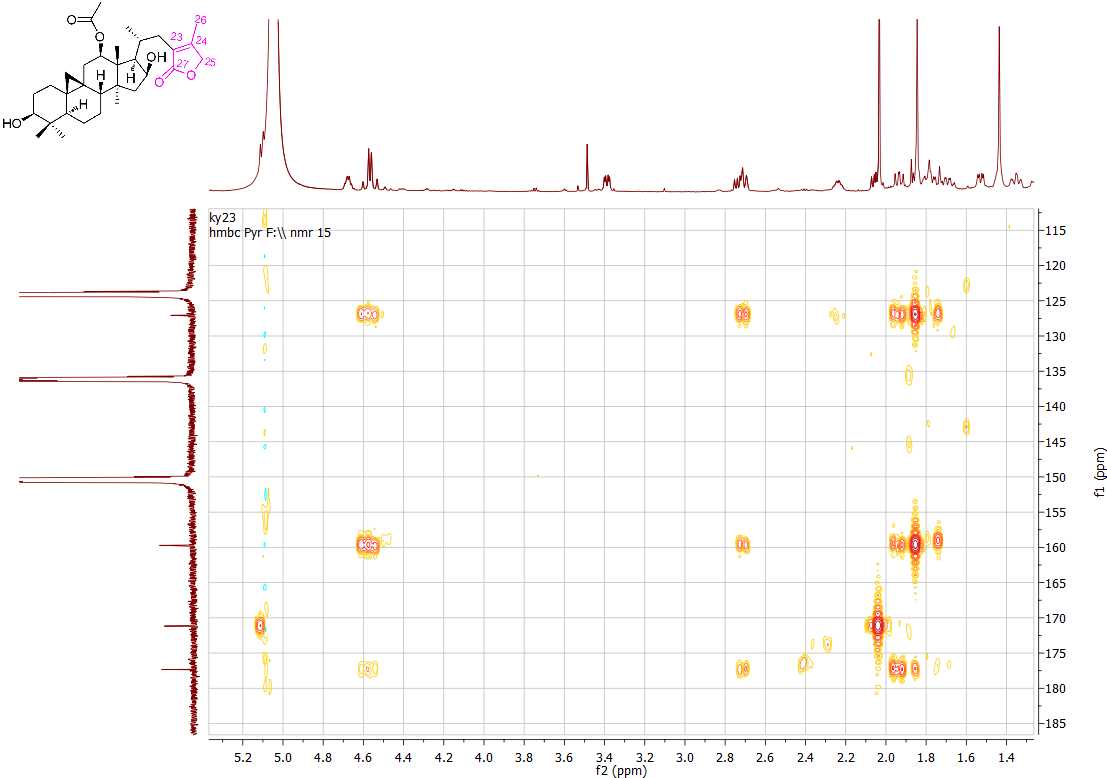


**Figure S64**. ^1^H-^1^H COSY (600 Hz) Spectrum of cimyunnin **D** (**4**)


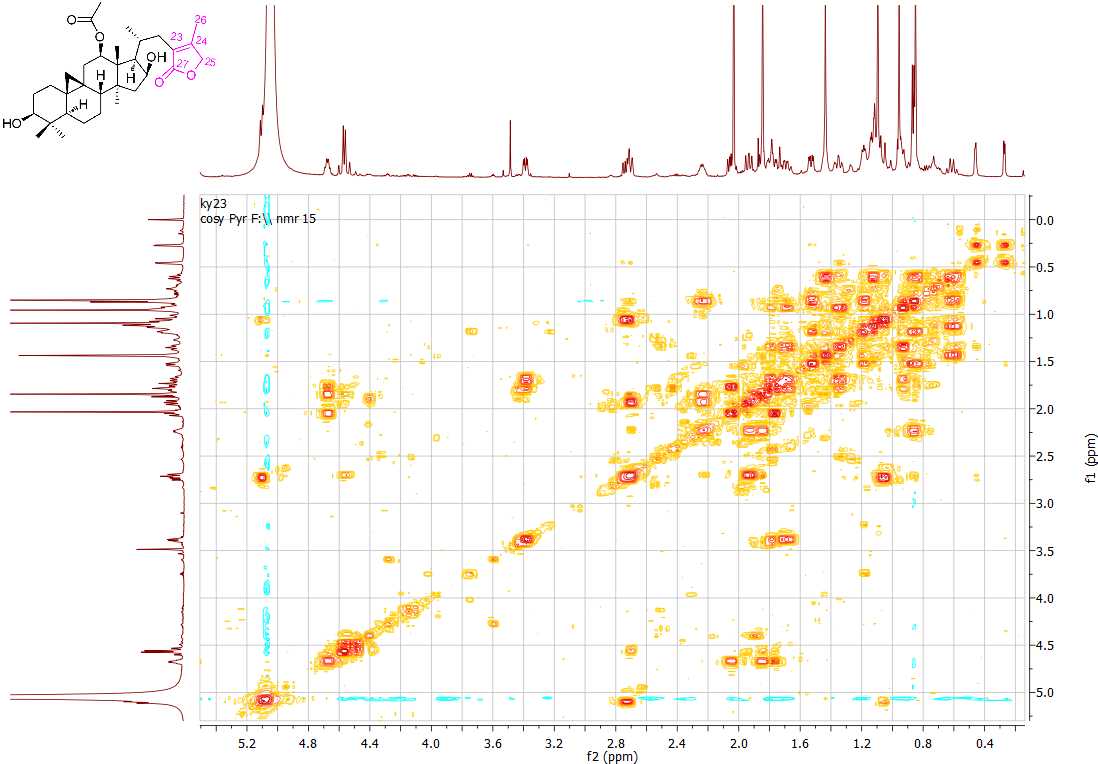


**Figure S65.** ROESY (600 Hz) Spectrum of cimyunnin **D** (**4**)


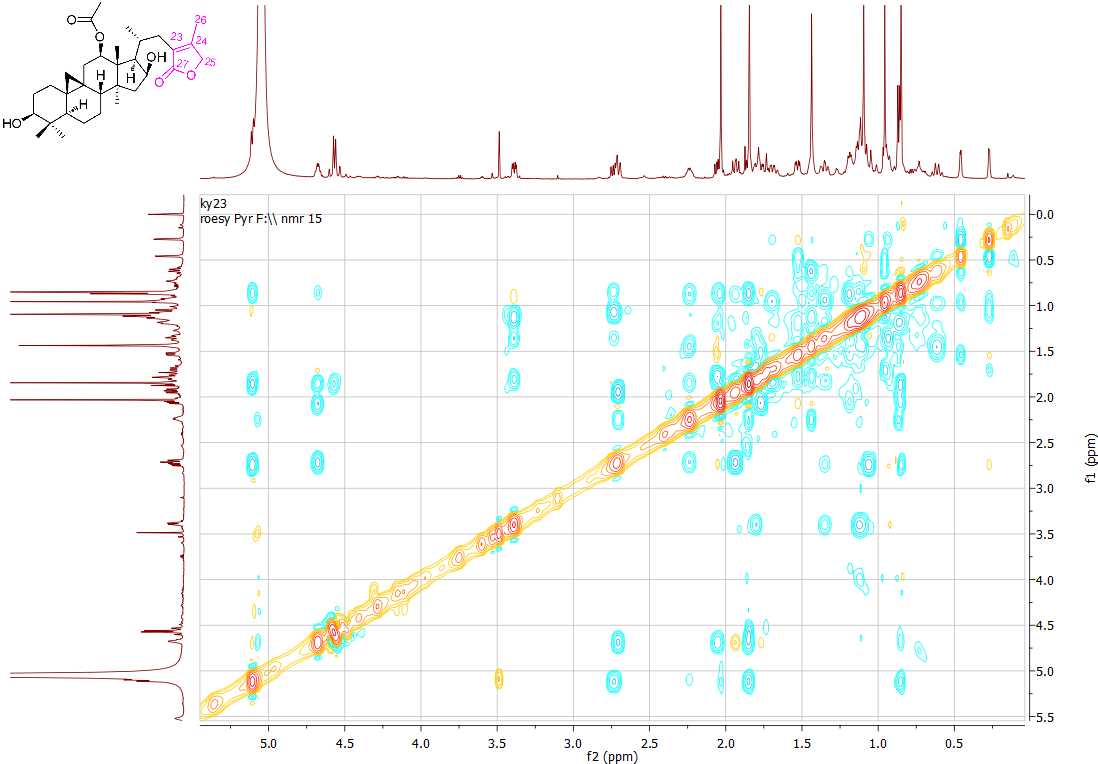


**Figure S66.** HREIMS of cimyunnin **D** (**4**)


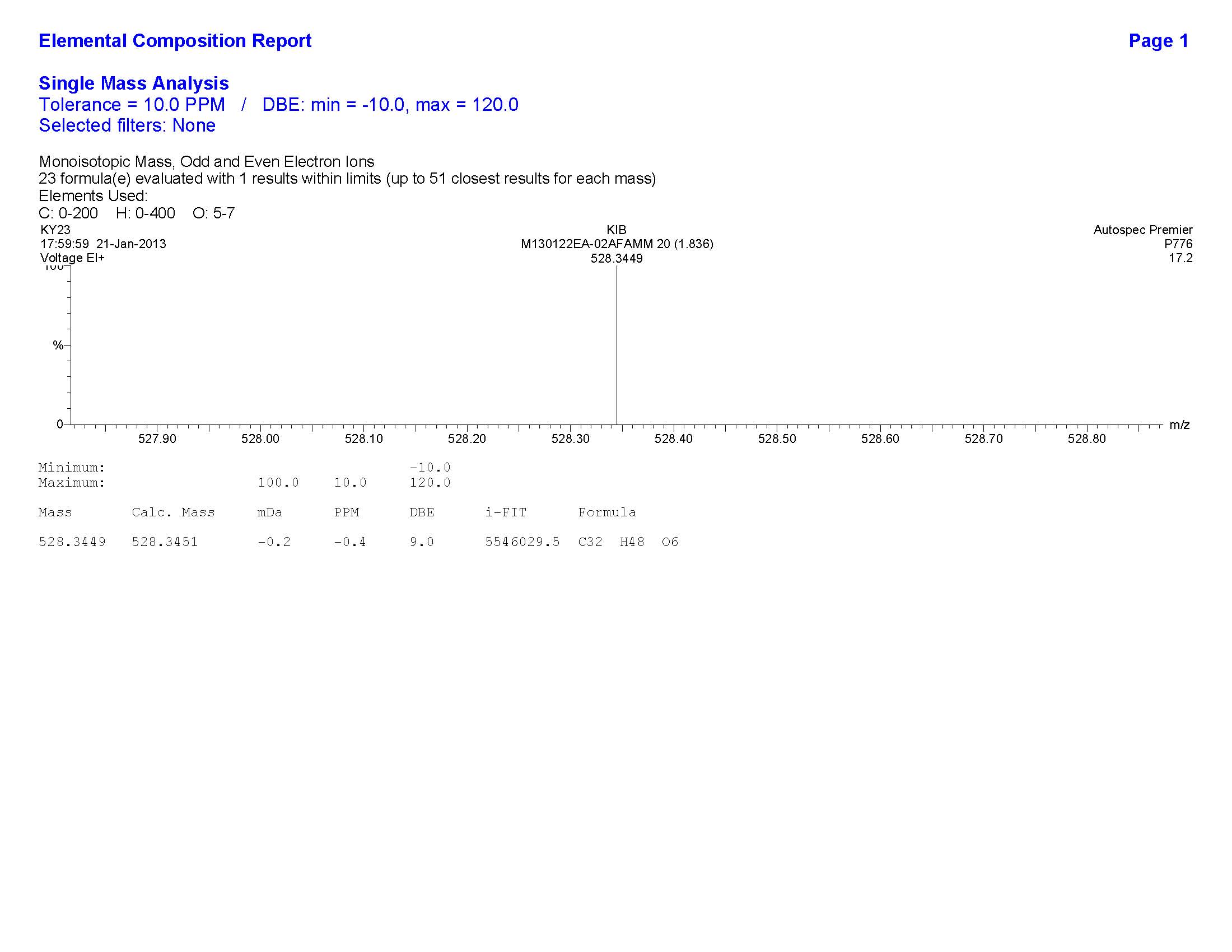


**Figure S67.** IR of cimyunnin **D** (**4**)

**Figure S68.** CD and UV of cimyunnin **D** (**4**)


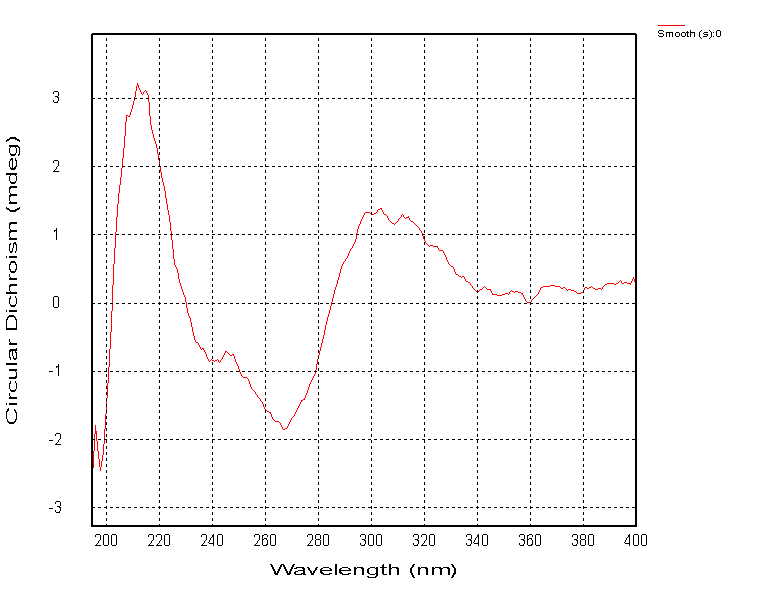

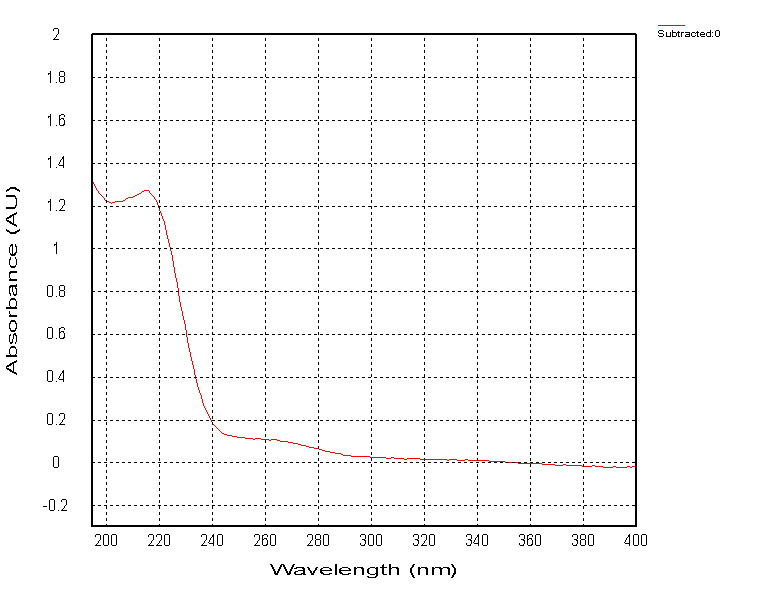


## 7. Full length gel and blots of VEGFR2 signalling pathway


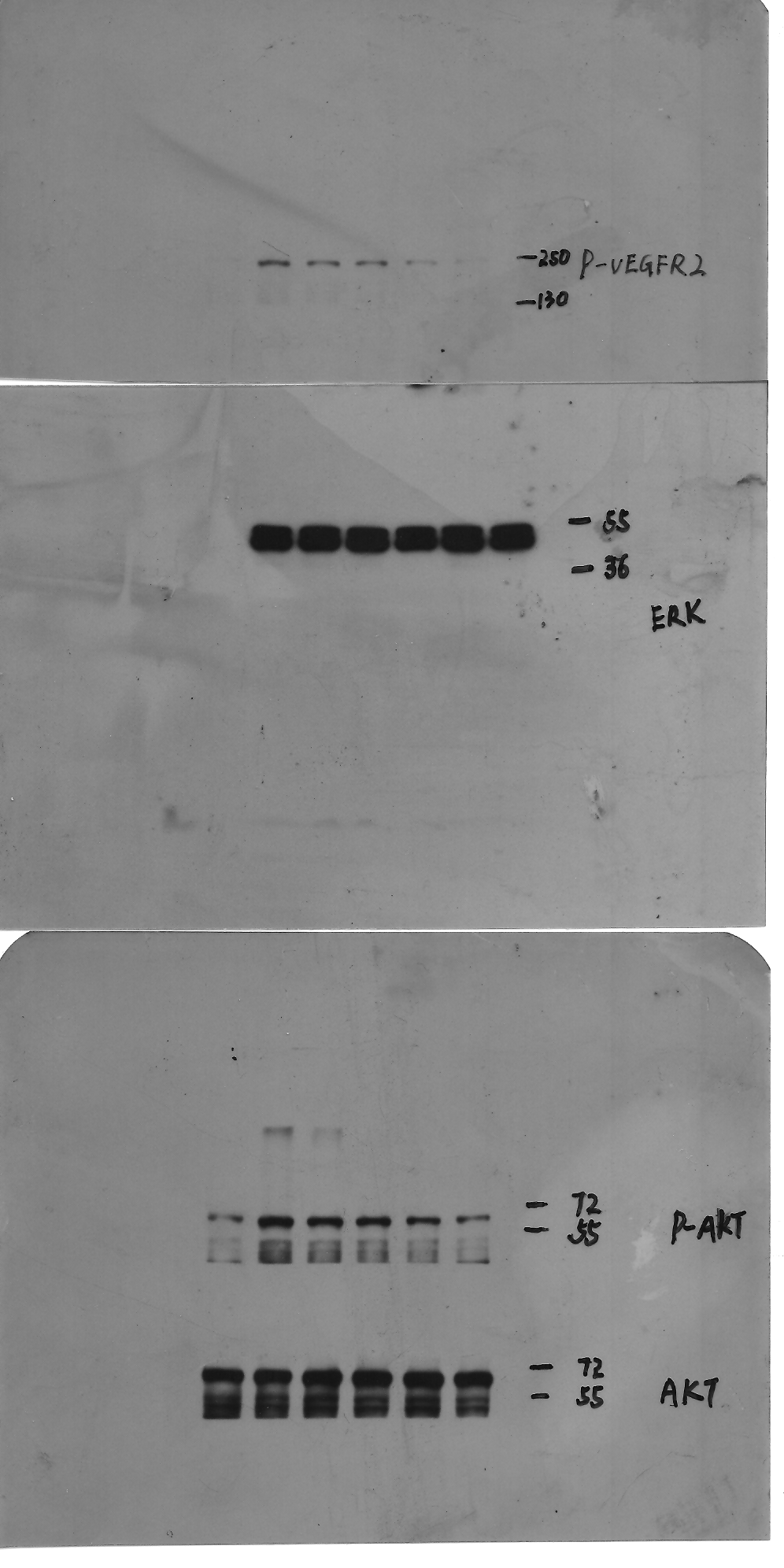

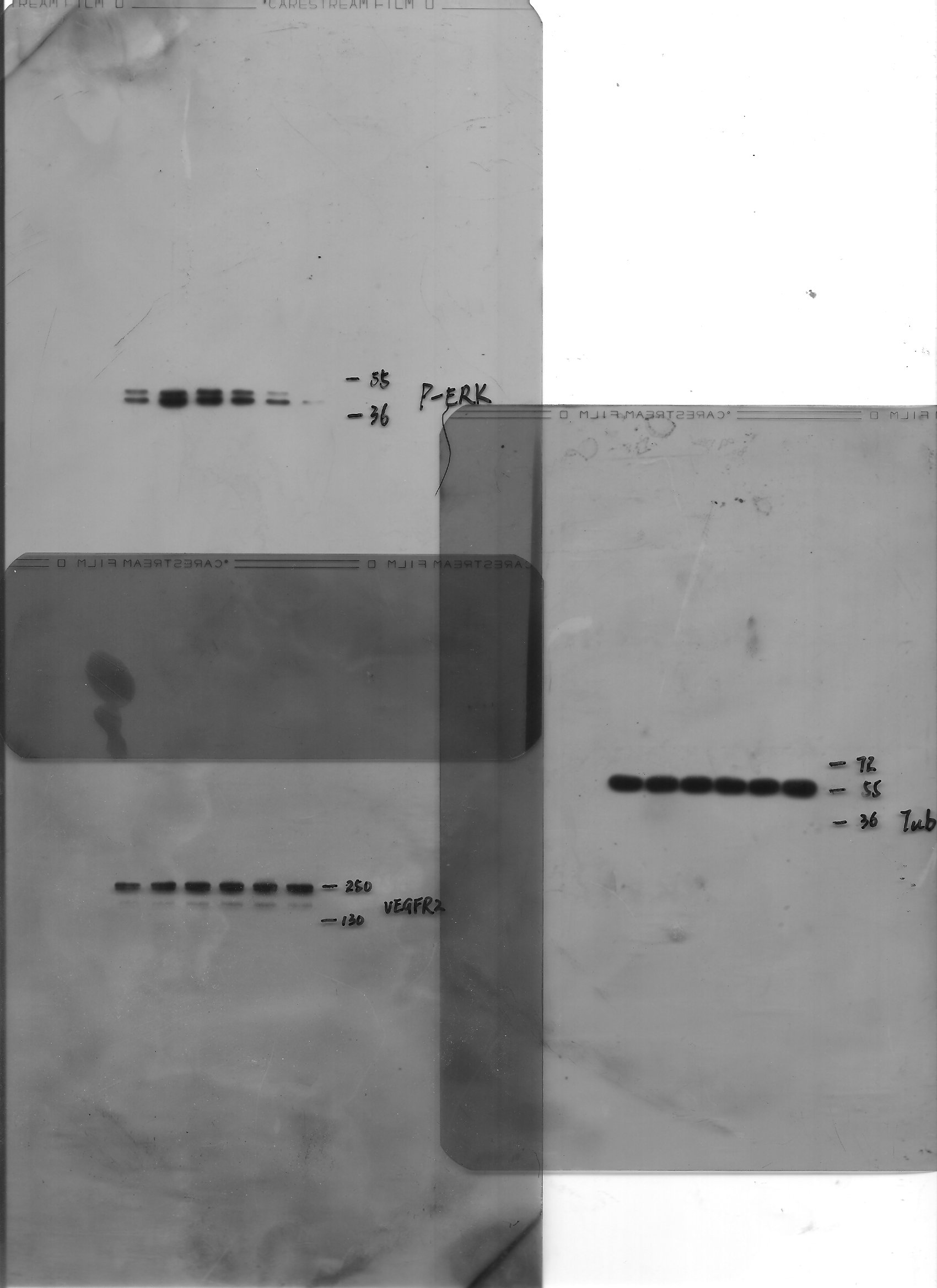

Supplement: Supplementary Information — for New Anti-angiogenic Leading Structure Discovered in the Fruit of Cimicifuga yunnanensis [file srep09026-s1.doc]
